# A fragment-based approach applied to a highly flexible target: Insights and challenges towards the inhibition of HSP70 isoforms

**DOI:** 10.1038/srep34701

**Published:** 2016-10-06

**Authors:** Alan M. Jones, Isaac M. Westwood, James D. Osborne, Thomas P. Matthews, Matthew D. Cheeseman, Martin G. Rowlands, Fiona Jeganathan, Rosemary Burke, Diane Lee, Nadia Kadi, Manjuan Liu, Meirion Richards, Craig McAndrew, Norhakim Yahya, Sarah E. Dobson, Keith Jones, Paul Workman, Ian Collins, Rob L. M. van Montfort

**Affiliations:** 1 Cancer Research UK Cancer Therapeutics Unit, Division of Cancer Therapeutics, The Institute of Cancer Research, London SM2 5NG, United Kingdom; 2 Division of Structural Biology, The Institute of Cancer Research, London SW3 6JB, United Kingdom

## Abstract

The heat shock protein 70s (HSP70s) are molecular chaperones implicated in many cancers and of significant interest as targets for novel cancer therapies. Several HSP70 inhibitors have been reported, but because the majority have poor physicochemical properties and for many the exact mode of action is poorly understood, more detailed mechanistic and structural insight into ligand-binding to HSP70s is urgently needed. Here we describe the first comprehensive fragment-based inhibitor exploration of an HSP70 enzyme, which yielded an amino-quinazoline fragment that was elaborated to a novel ATP binding site ligand with different physicochemical properties to known adenosine-based HSP70 inhibitors. Crystal structures of amino-quinazoline ligands bound to the different conformational states of the HSP70 nucleotide binding domain highlighted the challenges of a fragment-based approach when applied to this particular flexible enzyme class with an ATP-binding site that changes shape and size during its catalytic cycle. In these studies we showed that Ser275 is a key residue in the selective binding of ATP. Additionally, the structural data revealed a potential functional role for the ATP ribose moiety in priming the protein for the formation of the ATP-bound pre-hydrolysis complex by influencing the conformation of one of the phosphate binding loops.

The 70 kDa heat shock proteins (HSP70s) are an abundant family of ATP-dependent molecular chaperones, involved in many cellular processes including protein folding, prevention of protein aggregation, modulation of protein complexes, and protein transport between cellular compartments^1^. Because of their central role in cellular homeostasis, they have been implicated in several diseases including cancer, Alzheimer’s and Parkinson’s disease^1^. HSP70s bind to extended hydrophobic sequences in newly synthesised and partially folded proteins, in a manner dependent on a cycle of ATP hydrolysis and ADP/ATP exchange. This process is tightly controlled by co-chaperones such as the 40 kDa heat shock proteins (HSP40s) and nucleotide exchange factors (NEFs) including the Bcl2-associated athanogene (BAG) proteins^2^.

HSP70s consist of a conserved *N*-terminal nucleotide binding domain (NBD) with ATPase activity, a substrate binding domain (SBD), and a poorly conserved *C*-terminal domain which terminates with an EEVD tetratricopeptide repeat interaction motif^1^,^2^,^3^. The NBD and SBD are connected by a short inter-domain linker, which is crucial in allosteric communication between the domains. HSP70 enzymes are very flexible proteins that during their catalytic cycle adopt radically dissimilar conformations correlating with changes in binding affinity of substrate and nucleotide^4^.

The human HSP70 family has at least 17 members^3^,^5^. The two main cytosolic HSP70s are the ubiquitously expressed HSC70 (HSPA8), and the stress-induced HSP72 (HSPA1A)^3^. HSP72 is overexpressed in many tumours including breast, skin and oesophageal cancers, and haematological malignancies with overexpression often correlating with metastasis and poor outcome in cancer patients^6^,^7^,^8^,^9^. HSP72 is crucial for growth and survival of a range of different human tumour cell lines and elevated HSP72 expression reduces the cytotoxicity of pharmacological agents including HSP90 inhibitors^7^,^8^,^10^. Following our observation of the increased expression of HSC70 and HSP72 resulting from HSP90 inhibition^11^, we showed that dual siRNA silencing of HSC70 and HSP72 induced extensive apoptosis in human colon and ovarian cancer cell lines, but not in non-tumorigenic cell lines, demonstrating a therapeutic differential^10^. Thus, HSP70 inhibitors could be used as a single agent especially in cancers where HSP70 levels correlate with a poor prognosis^6^,^11^, or in combination with an HSP90 inhibitor or chemotherapy to achieve enhanced apoptosis in tumours.

Reported HSP70 inhibitors include 15-deoxyspergualin^12^, 2-phenylethynylsulfonamide (PES) or pifithrin-μ^13^, and the rhodacyanine dye MKT-077^14^ (Supplementary Fig. S1). Their mode of action remains unclear and their chemical properties render them unattractive as potential drugs^2^,^15^. However, the number of HSP70 inhibitors is increasing and includes a family of pyrimidinones^16^, the covalent inhibitor YK5 and its reversible analogues^17^,^18^, the natural product inhibitor oridonin^19^, HS-72^20^, the PES analogue PET-16^21^, the natural product inhibitor novolactone^22^, and the piperidine derivative HSP70-36^23^ (Supplementary Fig. S1). Most current HSP70 inhibitors act through covalent or non-covalent allosteric inhibition^21^,^22^. However, the adenosine-based inhibitor VER-155008 **1** (SPR *K*_d_ = 0.3 μM)^24^ demonstrates that, although designing ATP-binding site inhibitors is highly challenging^25^, it is possible to obtain new and cellularly-active ATP-competitive HSP70 inhibitors.

In this study, we explored ligand binding to the HSP70 ATP-binding site of HSC70 and HSP72. We focussed our studies on these two HSP70 isoforms because we previously discovered that the dual inhibition of HSC70 and HSP72 is essential for tumour selective apoptosis^10^. We reasoned that, although targeting the ATP-binding site was likely to be challenging, the high homology within the ATP-binding site of the two isoforms would allow us to achieve dual inhibition of the isoforms. In addition, it allowed us to carry out a fragment screen against the HSC70-NBD using surface plasmon resonance (SPR) followed by structural characterisation of the fragment binding modes in the HSC70/BAG1 crystal system^26^, which had already been confirmed to be suitable for crystallographic soaking experiments^24^. We discovered and characterised a fragment series based on an aminoquinazoline scaffold, which was elaborated to molecules with a higher affinity than the original fragment hit. These compounds provide a structural insight into the challenges of targeting sub-pockets within the flexible HSP70 ATP-binding site and may provide starting points for the design of ATP-binding site HSP70 inhibitors not containing the adenosine scaffold. A structural analysis of our ligand-bound structures and public domain structures shed further light on the functional role of residues in the ATP-binding site and identified Ser275 as a selectivity determinant for ATP-binding. Finally, in several HSC70-NBD/BAG1 structures we identified a distortion in the phosphate-binding loop 2, which is incompatible with phosphate binding and has a potential role in its ATPase function.

## Results and Discussion

### Exploring ligand binding in the HSC70 ATP-binding site

**Figure 1:**
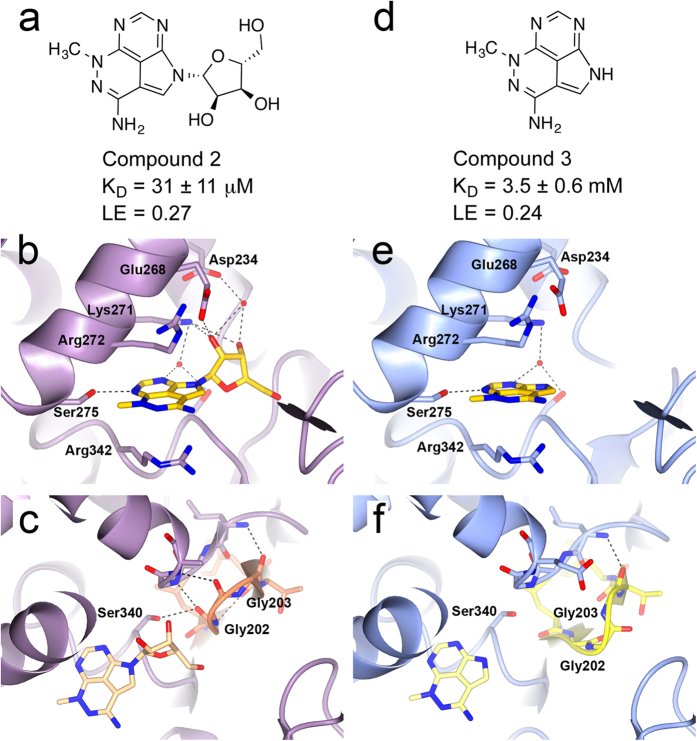
Binding mode of triciribine. (**a**) Chemical structure of the AKT inhibitor triciribine **2**. (**b**) Structure of **2** (yellow) bound to HCS70-NBD/BAG1 (purple). (**c**) Structure of HSC70-NBD/BAG1 (purple) bound to triciribine (beige) showing the regular conformation of the phosphate-binding loop 2 (orange). (**d**) Chemical structure of triciribine fragment **3**. (**e**) Structure of **3** (yellow) bound to HCS70-NBD/BAG1 (blue). (**f**) Structure of HSC70-NBD/BAG1 (blue) bound to **3** (light yellow) showing the flexibility in the phosphate-binding loop 2 (yellow) which disrupts the phosphate-binding pocket. LE = ligand efficiency^50^.

Preliminary exploration of the HSC70 ATP binding site using SPR and a truncated HSC70-NBD construct demonstrated binding of the AKT inhibitor triciribine **2** (Fig. 1a). A **2**-bound HSC70-NBD/BAG1 crystal structure (Fig. 1b) showed an overall binding mode similar to the adenosine of the bound ATP molecule in the published HSC70-NBD/BAG1 structure (PDB ID 3FZF, Supplementary Fig. S2)^24^. For example, the ribose 2′-hydroxyl forms hydrogen bonds with the side chains of both Glu268 and Lys271 and the 3′-hydroxyl interacts with Asp234 *via* the mediating water molecule, similar to the interactions of the ribose moiety in the ATP-bound HSC70-NBD/BAG1 structure. Likewise, the tricyclic adenine replacement is sandwiched between the aliphatic parts of the side chains of Arg342 and Arg272, similar to the adenine ring in the ATP-bound structure and both moieties form a hydrogen bond with the Ser275 hydroxyl (Fig. 1b, Supplementary Fig. S2). To probe the contribution of the tricyclic core, we synthesised the tricyclic triciribine fragment **3** (Supplementary Fig. S3) lacking the ribose group (Fig. 1d). The **3**-bound HSC70-NBD/BAG1 structure showed the same overall binding mode and confirmed the presence of the hydrogen bond with Ser275 (Fig. 1e).

A parallel deconstruction of 8-aminoadenosine **4** (PDB code 3 FHZ, Supplementary Fig. S2) from the inhibitor **1** series^24^, reinforced some of the findings described above. HSC70/BAG1 structures bound to adenine **5** and 8-aminoadenine **6** (Supplementary Fig. S2, SPR *K*_d_ values Supplementary Table S1) confirmed their binding in the adenine sub-pocket of the ATP-binding site in a similar orientation to the respective group in ATP and triciribine **2**, and both formed the anchoring hydrogen bond with Ser275.

Sequence conservation within the HSP70 family and the presence of the hydrogen bond interaction with the ligand in, to our knowledge, all available ATP-competitive ligand-bound HSP70 structures further highlights the importance of Ser275 and suggests that it might be a selectivity determinant for ATP-binding. We investigated this hypothesis by SPR, testing the binding of a series of purine-based nucleotides that can form the hydrogen bond with the Ser275 hydroxyl, and pyrimidine-based nucleotides not able to form the equivalent interaction (Supplementary Table S1). Of the purine-based nucleotides, ADP had the highest affinity with a *K*_d_ of 0.71 ± 0.32 μM, while GDP showed some evidence of binding to HSC70 (223 μM), albeit much weaker than ADP, and the pyrimidine-based nucleotides were all completely inactive (Supplementary Table S1). Binding of adenosine (*K*_d_ = 1.8 ± 0.52 mM, n = 3), ADP (*K*_d_ = 5.6 ± 2.4 μM, n = 3) and ATP (*K*_d_ = 2.1 μM ± 0.9 μM, n = 3) to a HSC70-NBD S275A mutant, unable to form the anchoring hydrogen bond, was between ~11 and 6-fold weaker compared to their affinities for the wild-type HSC70-NBD (WT HSC70-NBD K_d_s in Supplementary Table S1) and further confirmed the importance of the interaction.

Taken together, SPR analysis of the nucleotides and ATP-binding site ligands, and structural characterisation of 8-aminoadenosine and triciribine, encouraged us to embark on a fragment screening approach aimed to identify chemically diverse fragments binding in the HSC70 adenine sub-pocket, exploiting the Ser275 interaction hotspot, but with a desire to access other sub-pockets of the ATP-binding site.

### Fragment screening of HSC70

We carried out a SPR screen of our in-house library of 1962 fragments using truncated HSC70-NBD, enabling rapid structural characterisation of the fragment hits using the soakable HSC70-NBD/BAG1 crystal system^24^ and avoiding the need to deconvolute binding of hits to the SBD. To discriminate between fragments binding in the adenine sub-pocket, other sub-pockets in the ATP-binding site and/or potential secondary binding sites on the HSC70-NBD, we screened wild-type HSC70-NBD in parallel with two HSC70-NBD mutants (S275W and S275F), which block adenine binding to the HSC70 ATP-binding site. The relevance of these mutants was confirmed by their respective HSC70-NBD/BAG1 crystal structures, which showed that both mutations overlapped with the adenine ring of ATP in the ATP-bound HSC70-NBD/BAG1 complex (Supplementary Fig. S4). In addition, we confirmed by SPR that both mutants abolished binding of adenosine, ADP and **1**.

**Figure 2:**
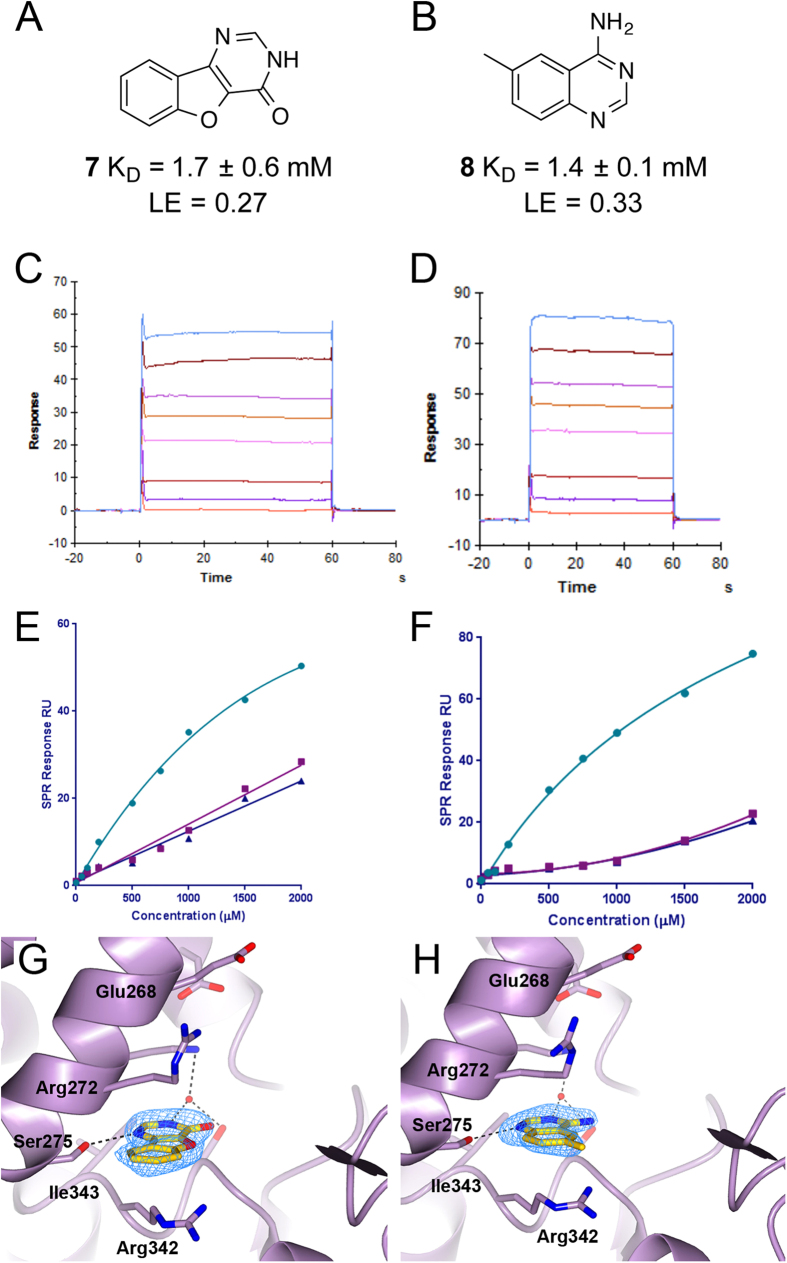
SPR fragment screening hits. (**A**) Chemical structure and HSC70-NBD SPR K_D_ of **7**. (**B**) Chemical structure and HSC70-NBD SPR K_D_ of **8**. (**C**) Concentration-response SPR experiment showing the binding of **7** to wild-type HSC70-NBD. (**D**) Concentration-response SPR experiment showing the binding of **8** to wild-type HSC70-NBD. (**E**) SPR binding curves for **7** binding to wild-type HSC70-NBD (light-blue) and to the S275W (purple) and S275F (blue) HSC70-NBD mutants. (**F**) SPR binding curves for **8** binding to wild-type HSC70-NBD (light-blue) and to the S275W (purple) and S275F (blue) HSC70-NBD mutants. (**G**) Structure of **7** (yellow) bound to the adenine sub-pocket in HSC70/BAG1. (**H**) Structure of **8** (yellow) bound to the adenine sub-pocket in HSC70/BAG1. In both panels the Fo-Fc electron density omit map is shown in blue and contoured at 3 σ.

The fragment screen yielded 3 fragments that bound to wild-type HSC70-NBD with lower *K*_d_ values than to the HSC70-NBD mutants and 7 fragments that bound with similar affinity to wild-type HSC70-NBD and the mutants. Binding of the latter fragments could not be confirmed in the HSC70-NBD/BAG1 crystal system, but fragment-bound HSC70-NBD/BAG1 structures were obtained for two of the three fragments showing preferential binding to wild-type HSC70. Both the tricyclic compound **7** (Fig. 2A,C,E) and the amino-quinazoline compound **8** (Fig. 2B,D,F) bind in the adenine sub-pocket of the HSC70 ATP-binding site (Fig. 2G,H), sandwiched between the Arg272 and Arg342 side chains, and form the anchoring hydrogen bond with the Ser275 hydroxyl. These findings are consistent with the SPR results, as the binding curves of both fragments showed reduced binding and a low degree of saturation for the adenine-blocked S275W and S275F HSC70-NBD mutants compared to wild-type HSC70-NBD (Fig. 2E,F).

### Structure-activity relationships (SAR)

We investigated functionalisation around the central cores of the two fragment hits. Based on a superposition of the **7**- and **8**-bound HSC70-NBD/BAG1 structures, we modified the carbonyl group of **7** to an amine moiety to incorporate a suitable attachment point to the tricyclic scaffold (Supplementary Fig. S3). Unfortunately, the resulting compound **9** lost all binding affinity, which, combined with the lower ligand efficiency of **7** compared to **8**, led us to deprioritise this chemotype.

**Figure 3:**
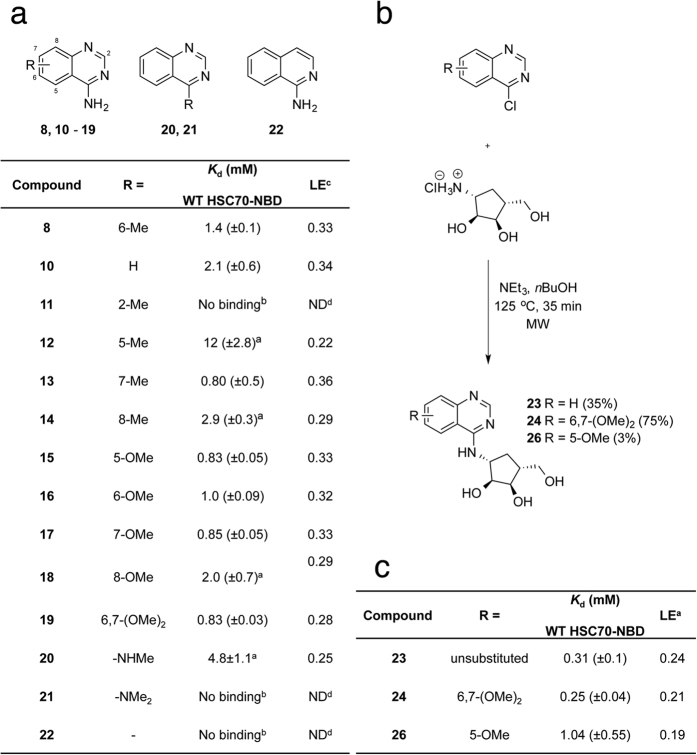
SAR of 4-aminoquinazoline fragments. (**a**) SPR measurements for compounds **8**, **10–22** on WT-HSC70-NBD were carried out in triplicate unless otherwise stated. Compounds did not bind to the S275W HSC70-NBD mutant. SPR *K*_d_ values are expressed as a mean ± standard deviation or standard error. ^a^Duplicate measurement. ^b^Single measurement. ^c^Ligand efficiency is calculated using the SPR *K*_d_. ^d^Not Determined. (**b**) Synthesis of cyclopentylaminotriol analogues **23**, **24** and **26** (**c**) SAR of cyclopentylaminotriol analogues. SPR measurements on WT-trHSC70 were carried out in triplicate. Compounds did not bind to the S275W HSC70-NBD mutant. SPR *K*_d_ values are expressed as a mean ± standard deviation or standard error. ^a^Ligand efficiency is calculated using the SPR *K*_d_.

**Figure 4:**
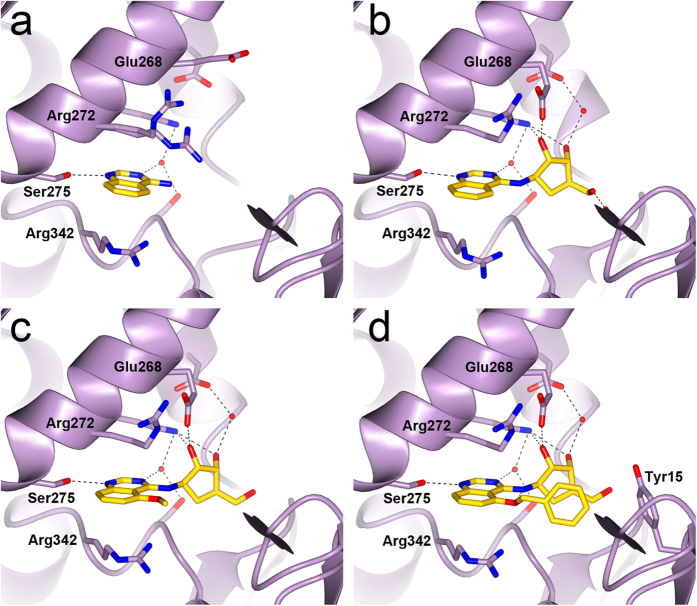
Elaboration of the aminoquinazoline scaffold. (**a**) Structure of **10** bound to HSC70-NBD/BAG1. (**b**) Structure of **23** bound to HSC70-NBD/BAG1. (**c**) Structure of **26** bound to HSC70-NBD/BAG1. (**d**) Structure of **28** bound to HSC70-NBD/BAG1. For corresponding SPR traces and binding curves see Supplementary Fig. S9.

Synthetic efforts concentrated on the aminoquinazoline **8**, which has a different binding mode compared to the core adenine heterocycle in adenosine and 8-aminoadenosine containing HSP70 ligands. The **8**-bound HSC70-NBD/BAG1 structure suggested that elaboration of the 6- and 7-positions of the quinazoline scaffold could be accommodated and the introduction of small methyl and methoxy groups would allow us to probe the binding mode stability of the scaffold. A set of quinazolines was prepared with methyl and methoxy groups incorporated in the 2-, 5-, 6-, 7-, and 8-positions and on the 4-amino group of **8** (Supplementary Fig. S5). SPR experiments showed that methyl and methoxy substitutions at the 5-, 6-, 7- and 8-positions (**12**–**18**) and a dual 6,7-dimethoxy substitution (**19**) were all tolerated with acceptable ligand efficiencies (Fig. 3a). However, introduction of a methyl substituent at the quinazoline 2-position, compound **11**, gave no detectable affinity. These results are consistent with the **8**-bound HSC70/BAG1 structure, which shows that the 5-, 6-, 7- and 8-positions are located on the more solvent exposed side of the quinazoline scaffold, whereas substitution at the 2-position would clash with Ile343 at the back of the adenine sub-pocket (Fig. 2H). Crystal structures of HSC70-NBD/BAG1 bound to the unsubstituted quinazoline scaffold **10** (Fig. 4a), the 7-methyl 4-aminoquinazoline **13** and the 6-methoxy 4-aminoquinazoline **16** all showed a similar binding mode to **8** (Supplementary Fig. S10), confirming the quinazoline scaffold as a stable and attractive start point for fragment elaboration. Critical to this binding mode is the interaction of the aminoquinazoline N1 atom with the Ser275 hydroxyl group, as was confirmed by the 1-aminoisoquinoline fragment **22** in which the N1 atom has been replaced by a methine group. This change resulted in a loss of binding affinity measured by SPR, but surprisingly a crystal structure of **22** bound to HSC70-NBD/BAG1 could still be obtained. The structure, which contained two independent HSC70-NBD/BAG1 complexes, revealed **22** interacting with the Ser275 hydroxyl through either its N2 atom, or its exocyclic amine group, consistent with its lack of activity (Supplementary Fig. S6).

### Growing into the ribose pocket

The difference in affinity between **3** and **2** (Fig. 1), between adenine and adenosine, and between 8-aminoadenine and 8-aminoadenosine (Supplementary Table S1) illustrates the importance of the ribose group in nucleotide binding. Structurally, this can be rationalised because the ribose 2′- and 3′-hydroxyls form an extensive interaction network with ATP-binding site residues as described above. In addition, we found an intriguing difference between the HSC70-NBD conformations of the adenine/adenosine, 8-aminoadenine/8-aminoadenosine and triciribine fragment/triciribine structural pairs. In the three structures with the ligands without the ribose, the phosphate-binding loop 2, which protrudes from the IIA NBD-subdomain and is one of two phosphate-binding loops characteristic for the actin ATPase family^27^, adopts a distorted β-hairpin conformation similar to that in the nucleotide-free HSC70-NBD/BAG1 structure (Fig. 1f). In this conformation, the peptide groups between Gly201 and Gly202 and between Gly202 and Gly203 are flipped relative to the nucleotide-bound conformation of phosphate-binding loop 2. By contrast, in the structures of the ribose-linked ligands, this loop is in a more open conformation in which the carbonyl groups of Gly201 and Gly202 interact with the α-helix at the back of the ATP-binding site (Fig. 1c). This conformation is similar to the one observed in the ATP-bound HSC70-NBD/BAG1 structure, in which it is required to provide space for the phosphate groups of the bound ATP molecule.

An overlay of **10** and ATP-bound HSC70-NBD/BAG1 structures showed that the adenine *N*-9 atom in ATP providing the linkage to the ribose ring, overlaps with the exocyclic 4-amino group in the fragment hit, suggesting a vector to grow the fragment into the ribose sub-pocket of the ATP-binding site (Supplementary Fig. S6). Modification of the 4-amino group with a single methyl group was tolerated and yielded **20** (Fig. 3a), strengthening this hypothesis. Synthetic attempts to directly link a ribose core to the 4-amino position of the quinazoline fragment were unsuccessful, but a less polar cyclopentylaminotriol ribose mimic − present in adenosine-like compounds with antiviral properties such as aristeromycin^28^ − could be readily introduced (Fig. 3b). Compared to parent fragment **10**, elaboration with the carbocyclic ribose mimic **23** resulted in a 7-fold increase in affinity (Fig. 3c). Likewise, introduction of the cyclopentylaminotriol to fragment analogue **19** resulted in a three-fold increase in affinity as exemplified by compound **24** (Fig. 3c). Compound **23** has a binding affinity similar to adenosine, with *K*_d_ values of 310 ± 100 μM and 150 ± 40 μM, respectively. However, the smaller number of polar atoms result in less hydrophilic calculated physical chemical properties (**23**; cLogP: 0.21, tPSA: 97.44) as compared to adenosine (cLogP: −1.17, tPSA: 136.26). Because the intrinsic high polarity of adenosine is a potential barrier to cellular permeability for molecules incorporating this core it suggests that the quinazoline scaffold may have more balanced physicochemical properties as a starting point for inhibitor discovery.

Elucidation of the **23**-bound HSC70-NBD/BAG1 structure confirmed the stability of the quinazoline binding mode and the preservation of hydrogen bonds by the carbocycle as compared to the ribose in the adenosine-bound HSC70-NBD/BAG1 structure (Fig. 4b). However, in structural terms addition of the carbocycle group does not completely mimic adenosine as Arg272 adopts an extended conformation in the adenosine-bound structure, but is found in the ‘up’-conformation in the **23**-bound structure. In addition, the phosphate-binding loop 2 adopts the distorted conformation observed in the nucleotide-free and quinazoline fragment-bound HSC70-NBD/BAG1 structures.

### Further elaboration of the quinazoline scaffold

To further improve the binding affinity of **23** we explored potential interactions between the Arg272 side chain and a suitably positioned functional group as is present in the **1**-bound HSC70-NBD/BAG1 structure. Addition of a methyl group at the *N*-4 position of **23**, yielding compound **25** (Supplementary Fig. S3) ablated affinity, indicating that modification at this position was not tolerated. However, compound **26** (Fig. 3c) showed that methoxylation at the 5-position of the quinazoline core was tolerated and its binding mode (Fig. 4c) suggested that elaboration at this position could orient a functional group close to Arg272. Compound **27**, bearing the 3,4-dichlorobenzyl substituent found in **1** (Supplementary Fig. S7) was equipotent with the 5-methoxy parent **26** which further confirmed this idea, but implied this specific substituent was sub-optimal (Table 1). Removal of the two chlorine atoms resulted in a modest increase in potency for compound **28** (Supplementary Fig. S7). However, compared to **26** the effect on ligand efficiency was minimal due to the increase in molecular weight of **28**.

The apparent difference in structure-activity relationships between the quinazoline scaffold and adenine-based inhibitors^24^ was probed further by modification of the 5′-hydroxyl position of the quinazoline-carbocycle scaffold with the 4-cyanobenzyl substituent found in **1**. This yielded **29** (Supplementary Fig. S7) with a reduced affinity compared to **28**, a result that could be readily explained by the **28**-bound HSC70-NBD/BAG1 structure (Fig. 4d). As expected, the aminoquinazoline carbocycle binds in a similar mode as observed for **23** and **26**, but instead of forming a π-stacking interaction with Arg272, the aryl group of the 5-substituent packs against the carbocycle, above Asp366, and forms an edge-face stacking interaction with Tyr15. In this binding mode the conformation of the 5-substituent of **29** would be incompatible with a π-stacking interaction with its 4-cyanobenzyl substituent, similar to the interaction observed for the aromatic substituents of **1**. Consequently, the π-stacking interactions of the two substituents with Arg272 and Tyr15 on respective sides of the binding cleft would also not be possible. To improve the affinity of **28** we replaced its phenyl group with a pyridine moiety (Supplementary Fig. S7), which yielded compound **30** with a binding affinity of 75 μM and shown to bind in the ATP-binding site by NMR (Supplementary Fig. S8).

### Ligand binding and NBD plasticity

HSP70 enzymes are extremely flexible proteins and the publicly available structural data sample a variety of conformational states. Structures of the HSP70 NBD can be broadly grouped in structures in complex with a nucleotide exchange factor such as BAG1, representing the open conformation of the NBD, and nucleotide-bound structures in absence of a nucleotide exchange factor, representing the closed conformation of the NBD. To our knowledge, only three crystal structures of non-nucleotide ATP-site ligands have been reported to bind to HSP70 isoforms that are not in complex with a nucleotide exchange factor: **1** bound to HSP72, and two different 8-*N*-quinoline aminoadenosine compounds bound to GRP78^29^ and HSP72^30^ respectively. However, all three compounds are based on an adenosine core scaffold and their ribose substituents form similar stacking interactions with Arg272. To increase our understanding of the binding of the quinazoline-based ligands to NBD conformational states different from the BAG1-bound form, we solved the structures of two key compounds, **23** and **28** bound to the HSP72-NBD in the absence of a nucleotide exchange factor.

**Figure 5:**
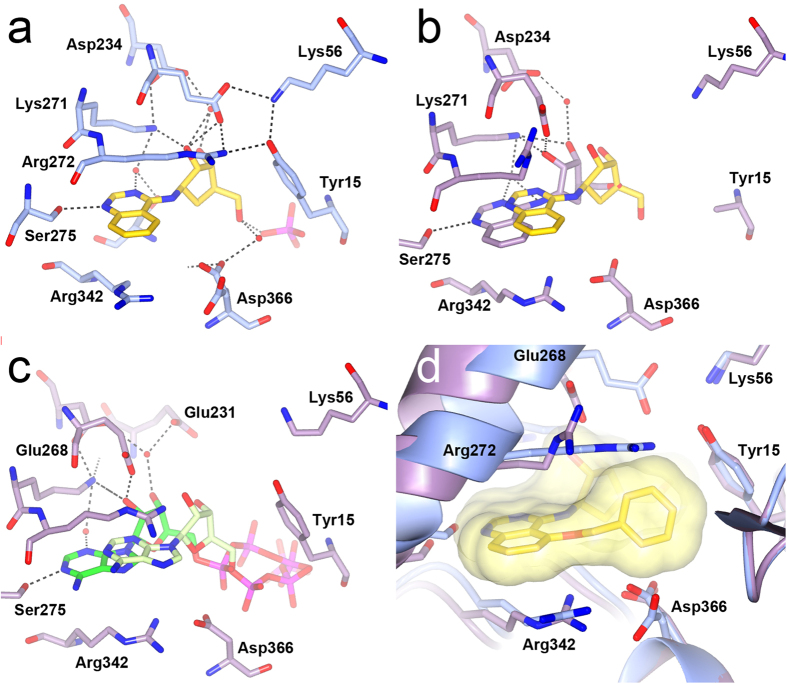
NBD flexibility and aminoquinazoline binding. (**a**) Structure of **23** (yellow) bound to the HSP72-NBD (light blue). (**b**) Superposition of open HSC70-NBD/BAG1 and closed HSP72-NBD **23**-bound structures showing the shift in compound position. Key HSC70-NBD protein residues and the HSC70-bound compound **23** are displayed in purple and superimposed HSP72-NBD bound compound **23** in yellow. (**c**) Superposition of the ATP-bound HSC70-BAG1 structure (PDB ID 3FZF) in the open conformation with the ATP-bound HSP72-NBD in the closed conformation showing the shift in the nucleotide. The bound ATP molecule is displayed in green and light green in the respective structures. HSP72-NBD residues are omitted for clarity in panel b and c. (**d**) Superposition of the HSP72-NBD structures bound to **23** and **28** showing the effects of the 5-*O*-benzyl substituent. The **28**-bound HSP72-NBD is shown in purple and the **23**-bound HSP72-NBD in light blue. Compound **28** is shown in yellow with a transparent molecular surface superimposed. Compound **23** is omitted for clarity.

As expected, the HSP72-NBD in the **23**-bound structure adopts a closed conformation (Fig. 5a) nearly identical to the one observed in the reported ADP-bound HSP72-NBD structure^31^ (PDB ID 1S3X) and the ligand superimposes well with the adenosine moiety of ADP in this structure. Comparing the **23**-bound HSP72-NBD and HSC70-NBD/BAG1 structures revealed a shift of approximately 2–3 Å in the position of **23** consistent with the difference in conformation of the NBD domain (Fig. 5b) and with the shifts reported for nucleotide-bound open and closed NBD conformations (Fig. 5c). The key hydrogen bonds between the carbocycle and the enzyme are all maintained in the **23**-bound HSP72-NBD structure (Fig. 5a,b). In addition, the side chain of Arg272 changed from its ‘up’ conformation in the HSC70/BAG1 structure to an extended conformation covering the amino quinazoline ring and completing the hydrogen-bond network between the side chains of Glu268, Lys56, Tyr15 that stabilised the closed NBD conformation^32^ (Fig. 5a).

The **28**-bound HSP72-NBD structure (Fig. 5d) adopts an intermediate conformation between the open and closed NBD conformations as observed in the **23**-bound structures. The ligand **28** also shifts to an intermediate location with respect to the positions of **23** in the open and closed structures. This is probably a result of the 5-*O*-benzyl substituent forming an edge-face stacking interaction with Tyr15, which prevents full closure of the NBD. Additionally, in the **28**-bound HSP72-NBD structure Arg272 adopts a conformation close to the ‘up’ conformation in the **28**-bound HSC70-NBD/BAG1, because an extended conformation would clash with the 5-*O*-benzyl group of **28** (Fig. 5d). Glu268 maintains the interaction with the 2′-hydroxyl of the carbocycle and forms a salt-bridge with Arg272, but does so in a conformation incompatible with the formation of the salt-bridge with Lys56, resulting in further disruption of the hydrogen bond network.

**Figure 6:**
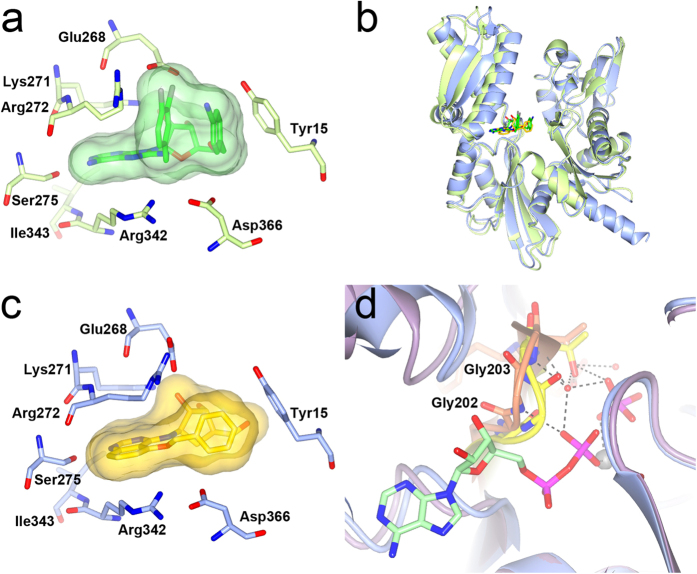
Comparison with VER-155008 (**1**) and conformational flexibility in the phosphate binding loop 2. (**a**) Close-up of VER-155008 (**1**, green) bound to the HSP72 NBD (light-green PDB ID: 4IO8). The compound surface is shown as a semi-transparent green surface. (**b**) Superposition of the **1**-bound (light green) and **28**-bound (blue) HSC72-NBD structures showing that the latter is in a more, but not completely, closed conformation. The ligands are displayed in green and yellow. (**c**) Close-up of the **28** (yellow) bound to the HSP72-NBD structure showing its 5-*O*-benzyl substituent occupies a similar area as the substituents of **1**, but interacts in a different way. The compound surface is shown as a semi-transparent yellow surface. (**d**) Superposition of the nucleotide-free HSC70-NBD/BAG1 structure (PDB ID 1HX1, purple) and the ADP/Pi bound HSP72 structure (PDB ID 1S3X, light blue) showing the effect of the phosphate-binding loop 2 conformations on its interactions with ADP (green). The respective conformations of the phosphate-loop 2 are shown in yellow and orange.

Schlecht *et al*. reported that **1** bound to the HSP70 NBD (Fig. 6a) in absence of BAG1 resulted in a more closed conformation of the NBD as compared to the ternary complex of **1** bound to HSC70-NBD/BAG1^15^. However, superposition of the respective structures shows that the position of **1** hardly changes. Interestingly, despite the 5-*O*-benzyl substituent of **28** occupying the same location as the two aromatic substituents of **1** (Fig. 6a,c), the differences in stacking interactions result in a further, but still incomplete, closure of the NBD in the **28**-bound HSP72-NBD structure (Fig. 6b). Therefore, potentially higher affinity aminoquinazoline ligands might be obtained if compounds could be functionalised to more effectively stabilise the open conformation of the HSP70 the NBD conformation, as represented by the HSC70/BAG1-bound structures.

### Conformational flexibility of phosphate binding loop 2

In the HSP70 ATP-hydrolysis cycle, ADP release is facilitated by the binding of NEFs such as the BAG proteins to the HSP70-NBD domain, which pulls apart the adenosine and phosphate-binding sub-pockets within the ATP-binding^26^,^33^,^34^. In the case of BAG2 binding to the HSC70-NBD, ADP-binding is further weakened by steric clashes^33^. These are absent in the HSC70-BAG1 complex^33^; however, we found differences in the phosphate-binding loop 2 conformation in HSC70-NBD/BAG1 structures, which may play a role in ADP-release and the rebinding of ATP (Figs 1f and 6d). Intriguingly, in all HSC70-NBD/BAG1 structures with ligands containing a ribose moiety that we solved as part of our fragment-based campaign, the phosphate-binding loop 2 adopts a regular β-hairpin conformation interacting with α-helix α6 (Fig. 1c) and is compatible with phosphate binding as observed in the ATP-bound open HSC70-NBD/BAG1 structure. By contrast, and to our surprise, in all HSC70-NBD/BAG1 structures bound to carbocycle-containing ligands and small fragments, phosphate-binding loop 2 adopts the distorted conformation also present in the nucleotide-free HSC70-NBD/BAG1 (Fig. 1f). Superposition of structures with the distorted phosphate-binding loop conformation and the ADP/Pi-bound post-hydrolysis HSP72-NBD structure (PDB ID 1S3X), shows that the amide groups of Gly202 and Gly203 in the distorted P-loop conformation cannot interact with the β-phosphate of ADP and the water molecule bridging the ADP β-phosphate and hydrolysed γ-phosphate ion (Fig. 6d). Therefore, this conformation would destabilise the binding of ADP in the HSC70/BAG1 complex and one could hypothesise that it is part of the mechanism of BAG1-mediated ADP-release. Although we do not understand the structural driver for the change in the conformation of phosphate-binding loop 2, it appears that the ribose unit, possibly in combination with the anchoring heterocycle binding in the adenine sub-pocket, is important in obtaining the phosphate-compatible conformation of the loop and therefore might play a role in ADP release and/or priming the enzyme for the binding of ATP.

## Conclusions

We have carried out a screen of 1962 fragments by SPR against HSC70 and confirmed two fragment hits binding in the adenine sub-pocket of the HSC70 ATP-binding site, representing a low hit rate. We have taken an amino-quinazoline hit and elaborated it into a compound with a different binding mode from the analogues of **1** and an alternative heterocyclic scaffold than adenosine. We showed that the closely related cyclopentylaminotriol group was not able to mimic the role of the ribose moiety completely and did not result in increased affinity compared to adenosine. Elaboration of the aminoquinazoline scaffold at the 5-position yielded compound **28**, which positions a benzyl group in the same general location as the substituents of the inhibitor **1**, but does so in a different manner. The **28**-bound HSP72-NBD structure, which cannot fully close due to a steric clash of the 5-*O*-benzyl group with Arg272, clearly illustrates that the ATP binding site in HSC70 and HSP72 closed states is extremely difficult to target with compounds significantly different from a nucleotide due to the enclosed nature of the binding site in its closed conformation. Possibly the most feasible way to exploit the ATP-binding site for the dual inhibition of HSC70 and HSP72 with a more drug-like scaffold than adenosine is through stabilisation of the open state of the conformationally flexible HSP70 NBD. Our aminoquinazoline ATP binding site ligands show that this approach remains extremely challenging, but is possible in principle.

Our studies also provide further insight into the functional role of key HSP70 ATP-binding site residues, including Ser275, which is an anchoring hotspot and an important selectivity determinant for initial ATP-binding. In addition, our findings identified a conformation of the phosphate-binding loop 2 in open HSP70-NBD/BAG1 structures that is incompatible with the position of the phosphates in the post-hydrolysis ADP/Pi complex (PDB ID 1S3X) and that might therefore be involved in ADP release in HSC70. The observed conformational plasticity upon the binding of ribose containing ligands could suggest a role for the ribose, possibly in combination with the adenine ring, in priming HSC70 for ATP-binding, Further work is required to establish the exact details of such a mechanism.

## Methods

### SPR fragment screening and follow-up experiments

SPR experiments were carried out using a Biacore T200 (GE Healthcare) at 25 °C. Amine coupling chemistry was used to immobilise the HSC70 NBD domain and S275W, S275F and S275A HSC70-NBD mutant proteins on a carboxymethyl-5′-dextran (CM5) sensor chip. The chip’s surface was activated with a 10 min injection of a 1:1 mixture of 100 mM *N*-hydroxysuccinimide and 400 mM 1-ethyl-3-(3-dimethylaminopropyl)-carbodiimide. The HSC70 proteins (70 μg/mL in 10 mM sodium acetate, 750 μM ADP, pH 5.0) were immobilised on the surface over a 10 min injection period. The remaining activated groups on the surface were blocked with a 7 min injection of 1 M ethanolamine at pH 8.5. For all the above procedures the flow rate was maintained at 10 μL/min and a 1x phosphate buffered saline running buffer (PBS running buffer: 10 mM NaHPO_4_/NaH_2_PO_4_ pH 7.4, 2.7 mM KCl, 137 mM NaCl) was used during immobilisation. Approximately ~13,000 response units (RU) of protein could be routinely captured on the CM5 chip on flow cells two to four. Flow cell one was left unmodified as the reference surface. Our in-house fragment library of 1962 fragments at a stock concentration of 100 mM in 100% (*v/v*) was screened. SPR screening plates were prepared by dispensing of 0.1 μL of each fragment in 384-well polypropylene V-bottomed plates (Greiner) using an ECHO 550 acoustic liquid dispenser (Labcyte) followed by addition of fresh DMSO and running buffer to give a final fragment concentration of 125 μM in 5% (*v/v*) DMSO in an assay volume of 80 μL.

A ‘clean screen’ against wild type HSC70-NBD to remove fragments exhibiting promiscuous binding^35^ identified 21 promiscuous binders. This equates to ~1% of the total fragment library, which is consistent with the <2% promiscuous binders generally observed in SPR fragment screening^36^. The clean screen was carried out at a flow rate of 30 μL/min and a sample injection time of 60 seconds. The dissociation was monitored for 60 seconds and the surface was not regenerated between sample injections.

The full fragment screen was carried out against freshly immobilised WT-HSC70 NBD and the S275W and S275F HSC70-NBD mutants, using the same experimental setting as for the clean screen and a final fragment concentration of 125 μM. Injections of running buffer and of 125 μM adenosine, which was used as a positive control to monitor the health of the protein, were carried out at intervals of 35 cycles throughout the screen. In addition, a DMSO correction curve, prepared as described in the GE manual, was used to correct the effects of the solvent on signal intensity. Raw SPR data from the single point screen was processed and analysed using Pipeline Pilot version 8.0.1.500^37^. Firstly, the reported binding level for each fragment was corrected by subtraction of the buffer blank. Secondly, the values were corrected for the molecular weight of each fragment and the binding was normalised to the binding level observed for the closest adenosine control. All fragments with a binding level higher than two standard deviations from the mean (equating to a ~27% greater response than adenosine) of the whole data set were classed as primary hits. However, fragments with a binding level exceeding two times the theoretical Rmax were excluded, as we considered this an indication of superstoichiometric binding. Finally, fragments showing preferred binding to wild-type HSC70-NBD *versus* the HSC70-NBD mutants with a blocked adenine sub pocket were identified by plotting the binding levels of the fragments to wild-type HSC70-NBD against the binding levels of the respective mutant HSC70-NBD variants and added to the primary hit list. In total this procedure yielded 54 initial fragment hits.

The initial fragment hits were further investigated in a three-point concentration-response experiment against freshly immobilised WT HSC70-NBD and the S275W HSC70-NBD mutants using the same experimental protocol as for the primary screen. Compound dilutions of 50, 100 and 200 μM were generated using the ECHO 550. A confirmed hit was defined as a fragment giving greater than 10% response compared to adenosine and these were prioritised on the basis of their concentration response, ratio of experimental and theoretical Rmax, and shape of the sensorgrams, and expected fast on/fast off binding kinetics. This yielded 36 confirmed hits, which were selected for compound integrity analysis by LC-MS and subsequent *K*_d_ determination by SPR using fresh samples. LC-MS experiments (see Supplementary Information) identified 8 fragments that showed evidence of compound degradation or unacceptably low purity, leaving 28 fragment hits for a full eight-point concentration-response experiment to determine their *K*_d_ values against wild-type HSC70-NBD and the S275W HSC70-NBD mutant. For *K*_d_ determinations of prioritised hits fresh 200 mM DMSO stocks were used in an eight-point concentration response range, from 50 μM to 2000 μM. For triciribine and adenosine a concentration range from 25 μM to 1000 μM was used, prepared from 100 mM DMSO stocks. Nucleotides were dissolved in double distilled water to give 10 mM stock solutions, then diluted 40X with DMSO and tested using a concentration range between 0.0625 μM and 2.5 μM.

### Crystallography

Crystallisation of the HSC70/BAG1 complex was based on a previously published method^32^ with some minor alterations. Apo crystals of the HSC70/BAG1 complex were grown at 18 °C in sitting drops by mixing equal volumes of protein solution (5–15 mg/mL) and precipitant solution containing 16–26% (*w*/*v*) aqueous PEG3350, 0.1 M Na-K tartrate, 0.1 M Tris/HCl pH 8.5 and 25% (*v*/*v*) glycerol. Rod-like crystals of approximate dimensions 20 × 20 × 200 μm typically grew overnight. To solve the protein-ligand structures, apo-crystals were soaked in solutions containing 5–10% (*w*/*v*) PEG3350, 0.1 M Na-K tartrate, 0.1 M Tris/HCl pH 8.5, 25% glycerol, 40–400 mM ligand and 0–20% (*v*/*v*) final concentration of DMSO for 4–24 h prior to flash cooling in liquid nitrogen. Co-crystals of the HSC70/BAG1 complex with adenosine, GDP and compound **3** were obtained as described above for the apo HSC70/BAG1 complex but with the following modification: the protein solution (10 mg/mL) was incubated with 5 mM adenosine, 100 mM GDP or 20 mM compound **3** (4% (*v*/*v*) final DMSO concentration) for 30 min on ice prior to crystallisation.

Purified HSP72 protein was thawed, buffer exchanged into fresh 100 mM HEPES pH 7.5, and then incubated with 5 mM adenosine for 30 min on ice prior to crystallisation. HSP72/adenosine co-crystals were grown at 18 °C in sitting drops by mixing equal volumes of protein solution (5–12 mg/mL) and precipitant solution containing 17–28% (*v*/*v*) PEG3350, 0.1 M HEPES pH 7.5, 2 mM MgCl_2_ and 2 mM NaH_2_PO_4_. Co-crystals of approximate dimensions 100 × 100 × 300 μm typically formed overnight. The HSP72/adenosine co-crystals were transferred into fresh solutions containing 15–20% (*w*/*v*) PEG3350, 0.1 M HEPES pH 7.5, 2 mM MgCl_2_, 2 mM NaH_2_PO_4_, 100 mM of compound **23** or **28** and 20% (*v*/*v*) DMSO and incubated for 16 h at 18 °C prior to flash cooling in liquid nitrogen.

X-ray diffraction data were collected at 100 K at Diamond Light Source (Oxfordshire, UK; beamlines I02, I04-1 and I24), ESRF (Grenoble, France; beamline BM16), or in-house on a Bruker Microstar with Pt^135^ CCD detector or a Rigaku FRX with Pilatus 300 K detector. Data were integrated with XDS^38^ or SAINT^39^. All data were imported to MTZ format with POINTLESS^40^, then scaled and merged with AIMLESS^40^ and the CCP4 suite^41^. The structures were solved by molecular replacement with PHASER^42^, with the PDB structures 1HX1 (HSC70/BAG1 complexes) or 1S3X (HSP72) as the search models after removal of all non-protein atoms. Structures were refined in iterative cycles of model building with COOT^43^ and refinement with BUSTER^44^. TLS groups were selected with PHENIX phenix.find_tls_groups^45^. Ligand restraints were generated with GRADE^46^ and MOGUL^47^. The final structure quality was checked with MOLPROBITY^48^. The data collection and refinement statistics are presented in Supplementary Table S2 and for Fo-Fc electron density figures for all structural data see Fig. S10. All structural figures were made with CCP4MG^49^.

### Structural alignments

All structural alignments were carried out in CCP4MG using the SSM superposition method. All superpositions of HSC70 and HSP72 NBD domains were aligned on their IA and IB domains using residue selection: A/and not A/185-364, except the superposition of the ATP and compound 26 bound HSC70-NBD/BAG1 structures, which were aligned on the IIA and IIB domain (residue selection A/185-364) in order to correctly identify the vector for elaboration into the ribose pocket.

### Chemistry

For a detailed description of synthetic chemistry methods see the Supplementary Information.

## Additional Information

**Accession codes**: Atomic coordinates and structure factors for the crystal structures of HSP70 with compounds be accessed using PDB codes: 5AQF, 5AQG, 5AQH, 5AQI, 5AQJ, 5AQK, 5AQL, 5AQM, 5AQN, 5AQO, 5AQP, 5AQQ, 5AQR, 5AQS, 5AQT, 5AQU, 5AQV, 5AQW, 5AQX and 5AQY.

**How to cite this article**: Jones, A. M. *et al*. A fragment-based approach applied to a highly flexible target: Insights and challenges towards the inhibition of HSP70 isoforms. *Sci. Rep.* **6**, 34701; doi: 10.1038/srep34701 (2016).

## Supplementary Material

Supplementary Information

## Figures and Tables

**Table 1:** Elaboration of the quinazoline-carbocycle scaffold.

Compound	R=	R′=	*K*_d_ (mM) WT HSC70-NBD	*K*_d_ (mM) S275W HSC70-NBD^a^	LE^b^
27	3,4-dichlorophenyl	H	0.85 (±0.26)	5	0.14
28	phenyl	H	0.37 (±0.03)	No binding	0.17
29	phenyl	4-cyanobenzyl	2.0 (±0.0)	2	0.10
30	2-pyridine	H	0.075 (±0.13)	No binding	0.20

SPR measurements on WT HSC70-NBD were carried out in triplicate. SPR *K*_d_ values are expressed as a mean ± standard deviation unless otherwise stated. ^a^Single measurement. ^b^Ligand efficiency is calculated using the SPR *K*_d_.
