# Supplementary material for: A fragment-based approach applied to a highly flexible target: Insights and challenges towards the inhibition of HSP70 isoforms
Source: Sci Rep. 2016 Oct 6;6:34701. doi: 10.1038/srep34701 (PMC5052559; doi:10.1038/srep34701)
Supplement: Supplementary Information [file srep34701-s1.pdf]

Supporting Information

Fragment-based screening applied to a highly flexible target: Insights and challenges towards the inhibition of HSP70 isoforms

Alan M. Jones[1], Isaac M. Westwood[1,2], James D. Osborne[1], Thomas P. Matthews[1], Matthew D. Cheeseman[1], Martin G. Rowlands[1], Fiona Jeganathan[1], Rosemary Burke[1], Diane Lee[1], Nadia Kadi[1], Manjuan Liu[1], Meirion Richards[1], Craig McAndrew[1], Norhakim Yahya[1], Sarah E. Dobson[1,2], Keith Jones[1], Paul Workman[1], Ian Collins[1], Rob L. M. van Montfort[1,2].

[1] Cancer Research UK Cancer Therapeutics Unit, Division of Cancer Therapeutics, The Institute of Cancer Research, London SM2 5NG, United Kingdom.

[2] Division of Structural Biology, The Institute of Cancer Research, London SW3 6JB, United Kingdom.

KEYWORDS. HSP70, SPR, Fragment screening, ATP competitive inhibition, X-ray crystallography

Contents:

1) Figure S1: Reported HSP70 inhibitors
2) Figure S2: Nucleotide binding and deconstruction of 8-aminoadenosine.
3) Figure S3: Synthetic routes to: A) the triciribine fragment **3**, B) fragment **9**, and C) compound **25**
4) Table S1: Deconstruction of 8-aminoadenosine and binding of nucleotides
5) Figure S4: HSC70 inactive mutants
6) Figure S5: Synthesis of 4-aminoquinazoline analogues.
7) Figure S6: Fragment binding mode stability and growing vector
8) Figure S7: Synthetic route to 5-*O*-substituted 4-aminopquinazolines
9) Figure S8: Ligand observed NMR data for compound binding of **30** to HSP72
10) Figure S9: SPR data for key compounds
11) Figure S10: Fo-Fc electron density figures for ligand-bound structures and S275W/S275F HSC70-NBD mutants
12) Materials and Methods
    - Chemistry, general experimental and preparation of compounds **3**, **9**, **11-18**, **20**, **21** and **23-30**
    - LC-MS compound quality control methodology
    - Protein Production and site-directed mutagenesis
    - Protein intact mass spectrometry
    - Ligand observed NMR experiments
13) Table S2: Data collection and refinement statistics for crystal structures of trHSC70/BAG1 complex or trHSP72 ATPases with ligands.
14) Supplementary references

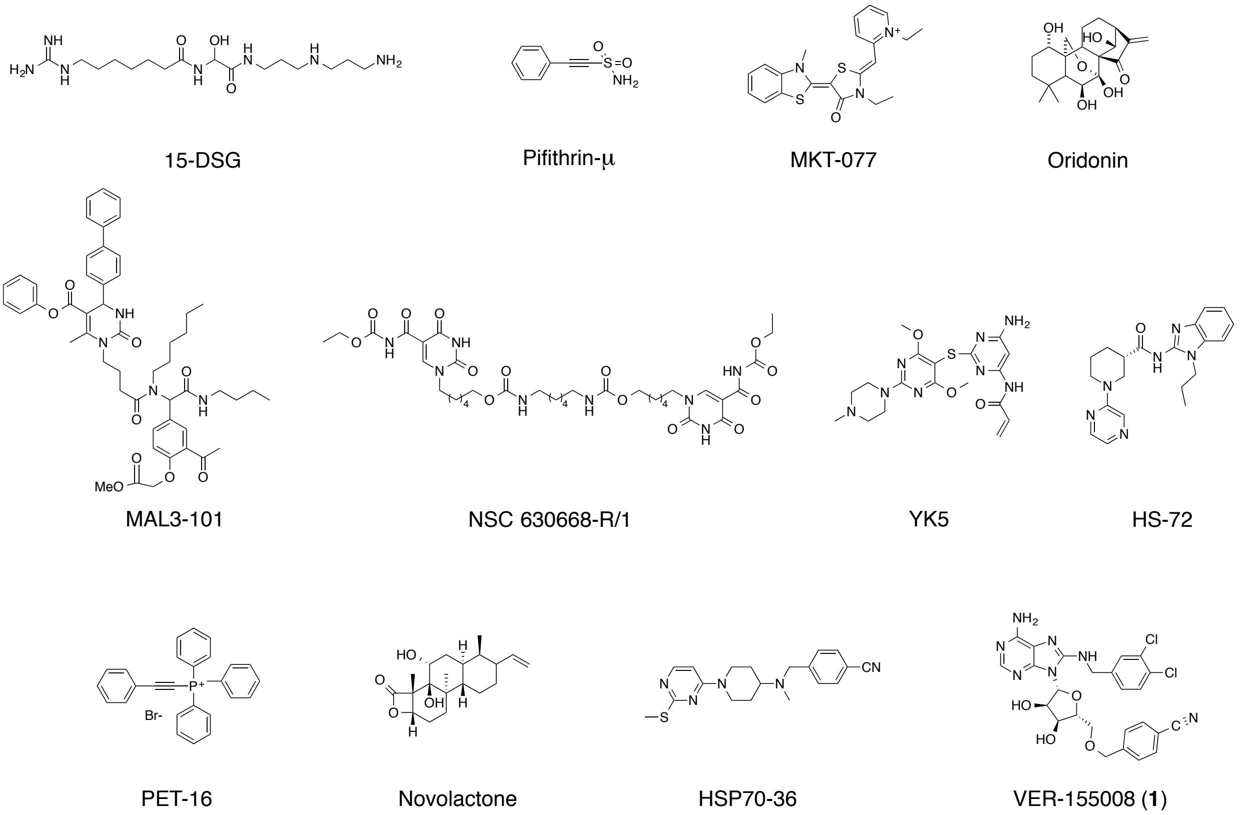

Figure S1. Reported HSP70 inhibitors. Panel of known HSP70 inhibitors with a variety of mode of actions. From left to right, top row: 15-DSG, PES/pifithrin-μ, MKT-077 and oridonin. Middle row: MAL3-101, NSC 630668-R/1, YK5 and HS-72. Bottom row: PET-16, novolactone, HSP70-36 and VER-155008 (**1**).

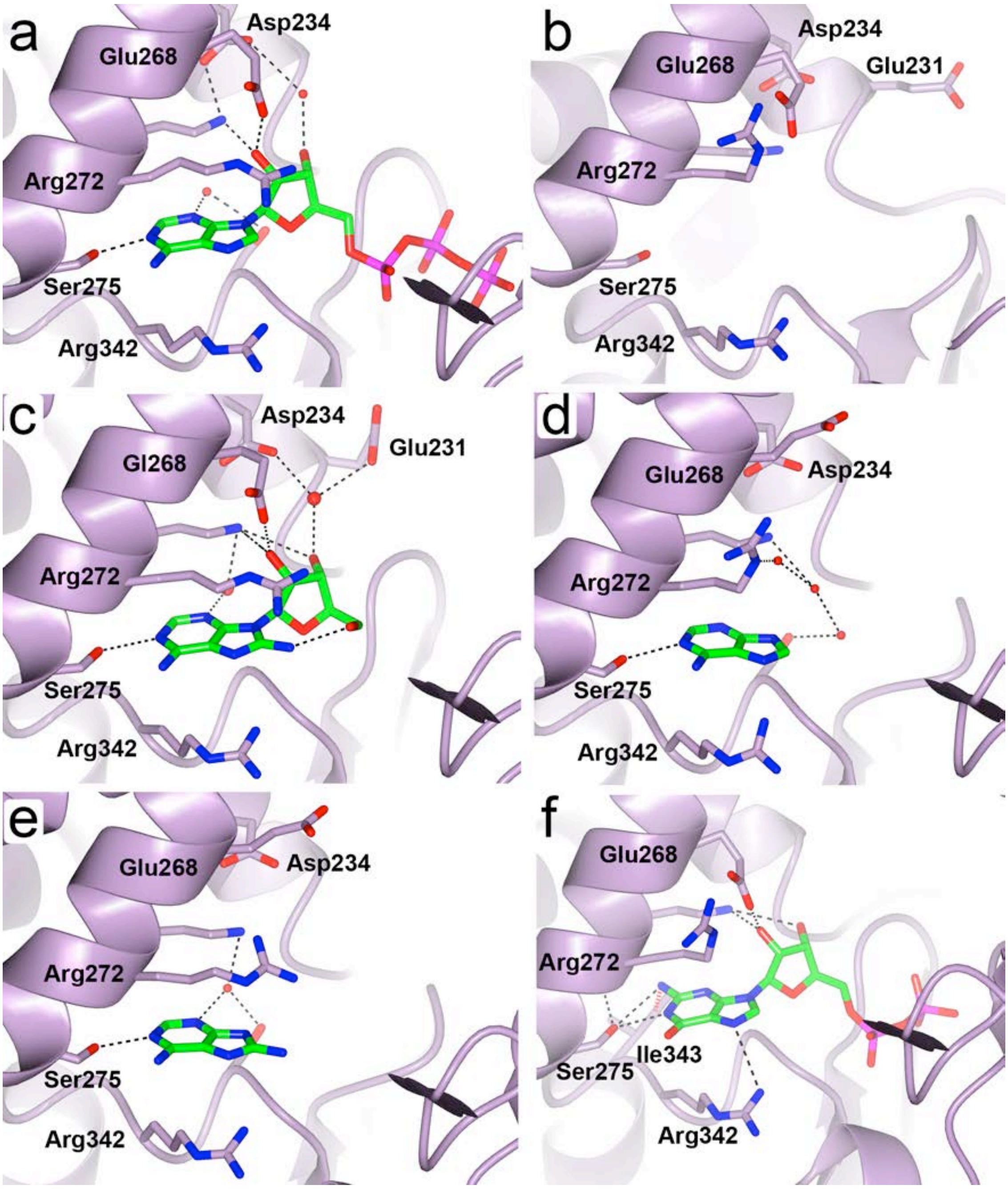

Figure S2. Nucleotide binding and deconstruction of 8-aminoadenosine. a) Crystal structure of ATP bound to HSC70/BAG1 (PDB ID 3FZF). b) Crystal structure of apo-HSC70/BAG1 (PDB ID 1HX1). c) Crystal structure of 8-aminoadenosine bound to HSC70/BAG1 (PDB ID 3FZH). d) Crystal structure of adenine bound to HSC70/BAG1. e) Crystal structure of 8-aminoadenine bound to HSC70/BAG1. f) Crystal structure of GDP bound to HSC70/BAG1 confirming the presence of the Ser275-N1 hydrogen bond. The weak binding of GDP can be explained by a steric clash of its exocyclic amino group with Ile343 at the back of the ATP-binding site. In all panels HSC70 is shown in purple and the respective ligands in green. Hydrogen bonds are shown as black dotted lines and selected residues are labeled. Water molecules are shown as red spheres.

a

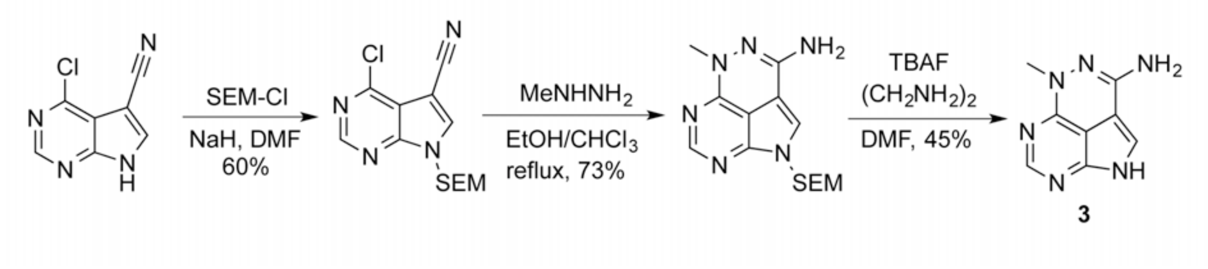

b

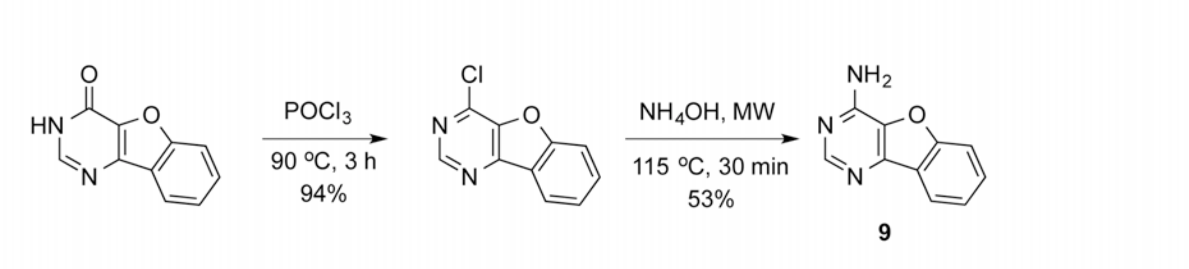

c

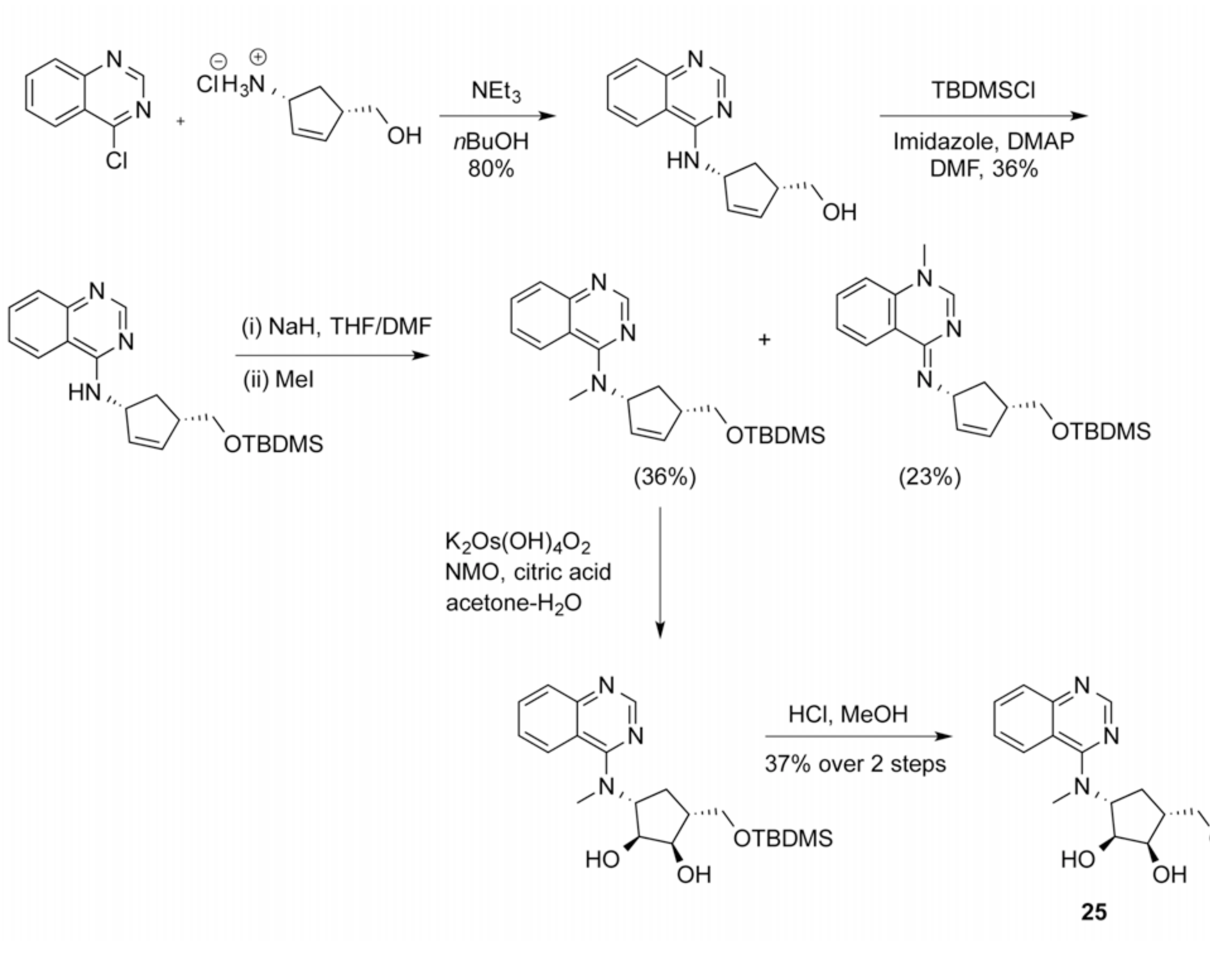

Figure S3. Synthetic routes to: a) the triciribine fragment **3**, b) fragment **9**, and c) compound **25**

**Compound**	**K_d (μM)** **WT HSC70-NBD**	**LE[a]**
8-aminoadenosine (**4**)	8 (±2)	0.35
Adenine (**5**)	No binding[b]	ND[c]
8-amino adenine (**6**)	2100 (±170)	0.34
Adenosine	165 (±37)[d]	0.28
ATP	0.33 (±0.10)[d]	0.29
ADP	0. 71 (±0.32)[d]	0.31
GDP	223[e]	0.18
TDP	No binding	ND
CDP	No binding	ND
UDP	No binding	ND

Table S1. Deconstruction of 8-aminoadenosine (**4)** and binding of nucleotides. SPR measurements were carried out in triplicate and are expressed as a mean ± standard deviation unless otherwise stated. [a]Ligand efficiency is calculated based on the SPR K_d. [b]No binding is defined as a binding level ≤10RU at 1mM. [c]ND = not determined. [d]Number of replicates is 4. [e]Single measurement.

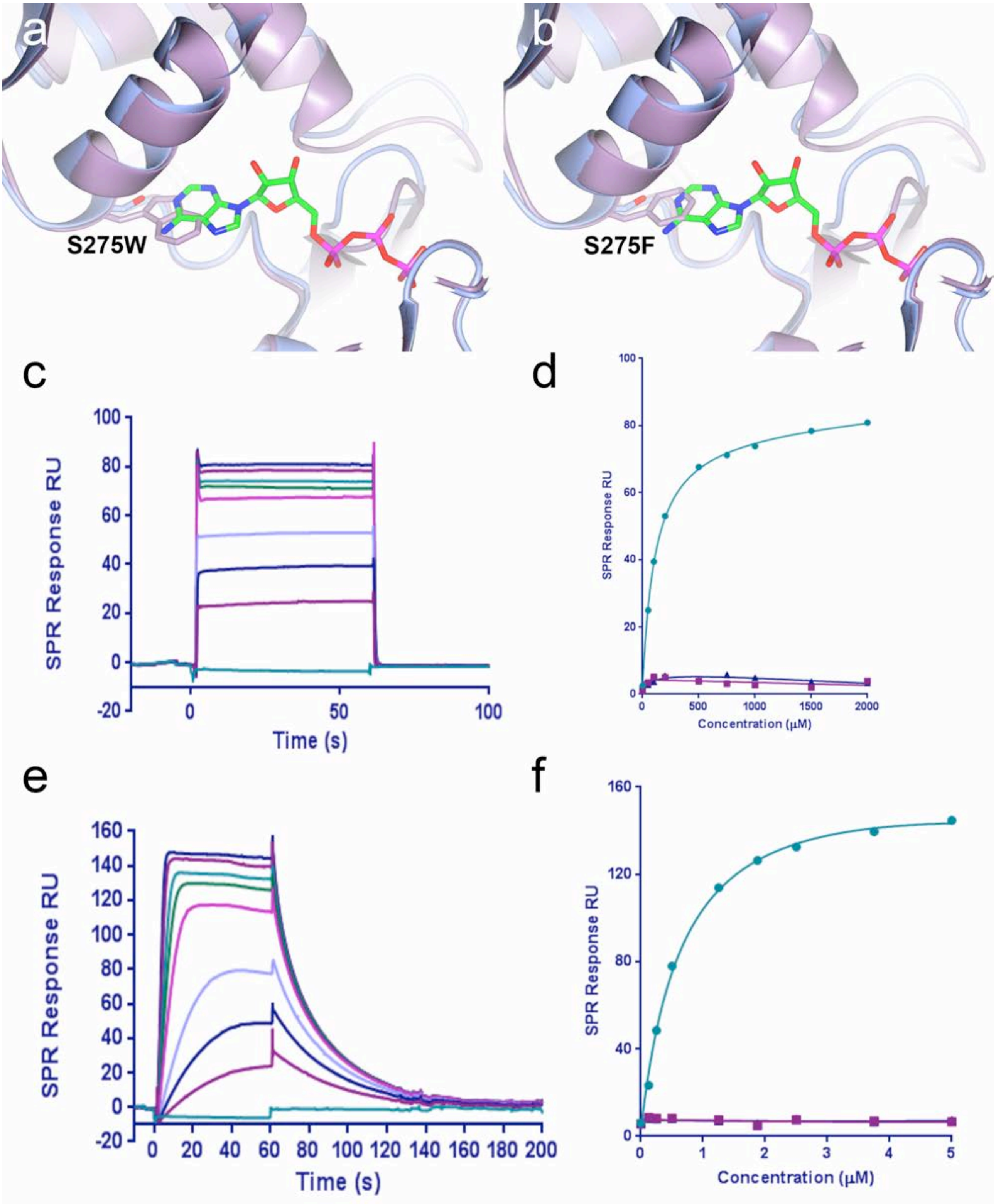

Figure S4. HSC70 inactive mutants. a) Superposition of the S275W HSC70-NBD/BAG1 and ATP-bound HSC70-NBD/BAG1 (PDB ID 3FZF) structures. b) Superposition of the S275F HSC70-NBD/BAG1 and ATP-bound HSC70-NBD/BAG1 structure. Both structures demonstrate the clash with the adenine ring of ATP. c) SPR traces showing the binding of adenosine to wild-type HSC70-NBD. d) SPR binding curves showing binding of adenosine binding to wild-type HSC70-NBD (turquoise) and not to the S275W (purple) and S275F (blue) mutants. e) SPR traces showing the binding of **1** to wild-type HSC70-NBD. f)

Binding curves showing binding of **1** to wild-type HSC70-NBD (turquoise) and not to the S275W (purple) and S275F (blue) mutants.

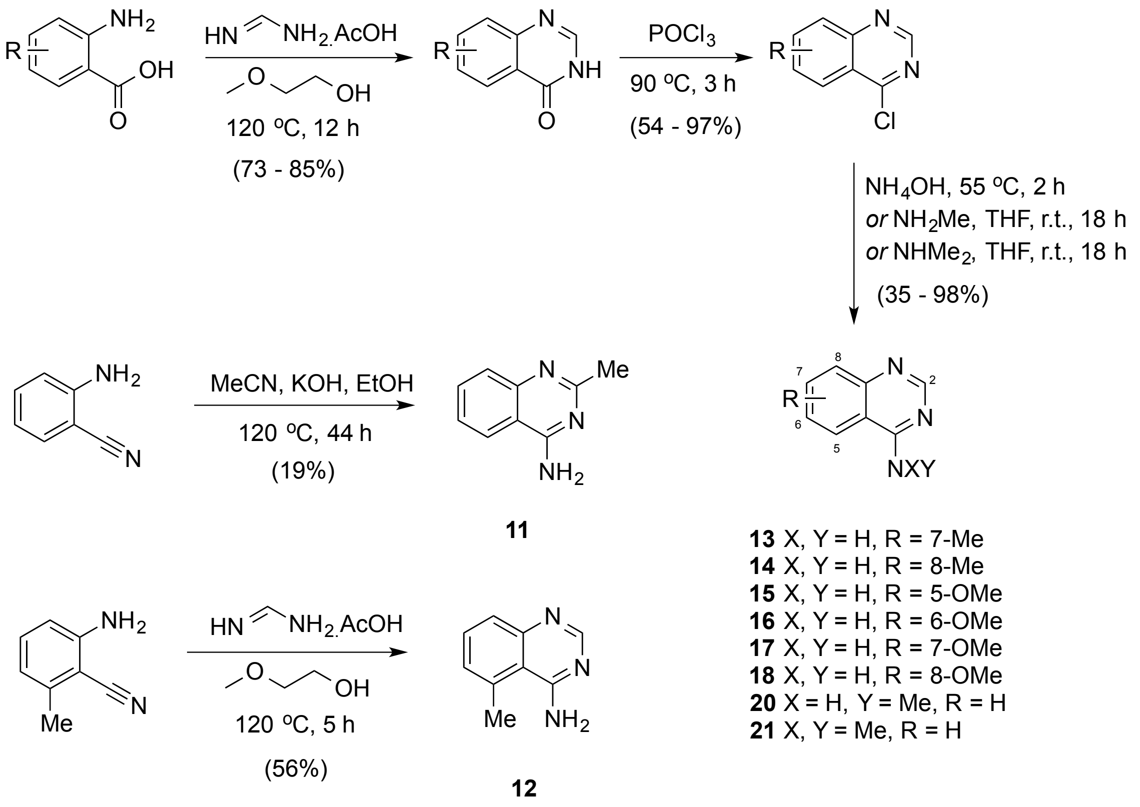

Figure S5. Synthesis of 4-aminoquinazoline analogues.

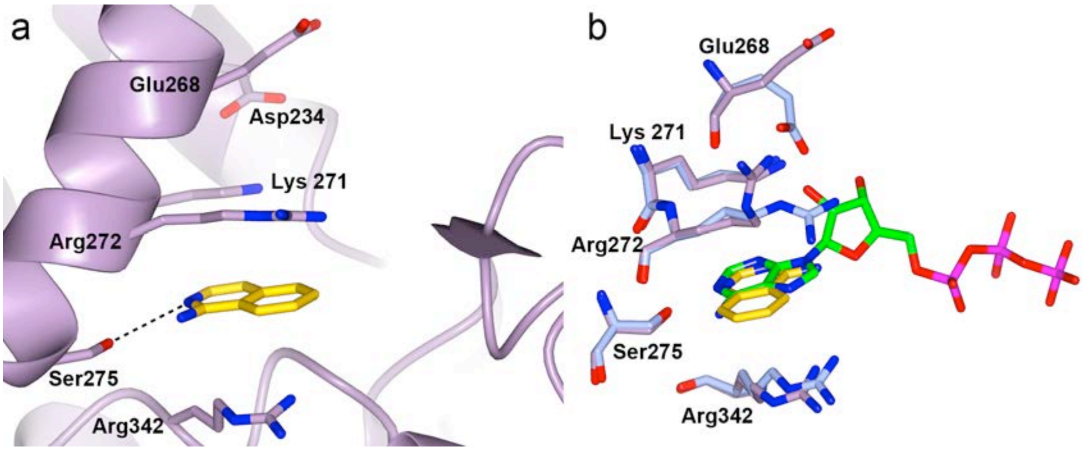

Figure S6. Fragment binding mode stability and growing vector. a) HSC70-NBD/BAG1 complex bound to compound **22** showing the different binding mode as compared to the aminoquinazoline **8**. HSC70 is shown in purple with key residues displayed in cylinder representation. Compound **22** is shown with carbon atoms displayed in yellow. b) Superposition of the HSC70-NBD/BAG1 complex (purple) bound to the unsubstituted aminoquinazoline **10** (yellow) with the HSC70-NBD/BAG1 complex (PDB ID 3FZF, light blue) bound to ATP (green), suggesting the vector to grow into the ribose pocket.

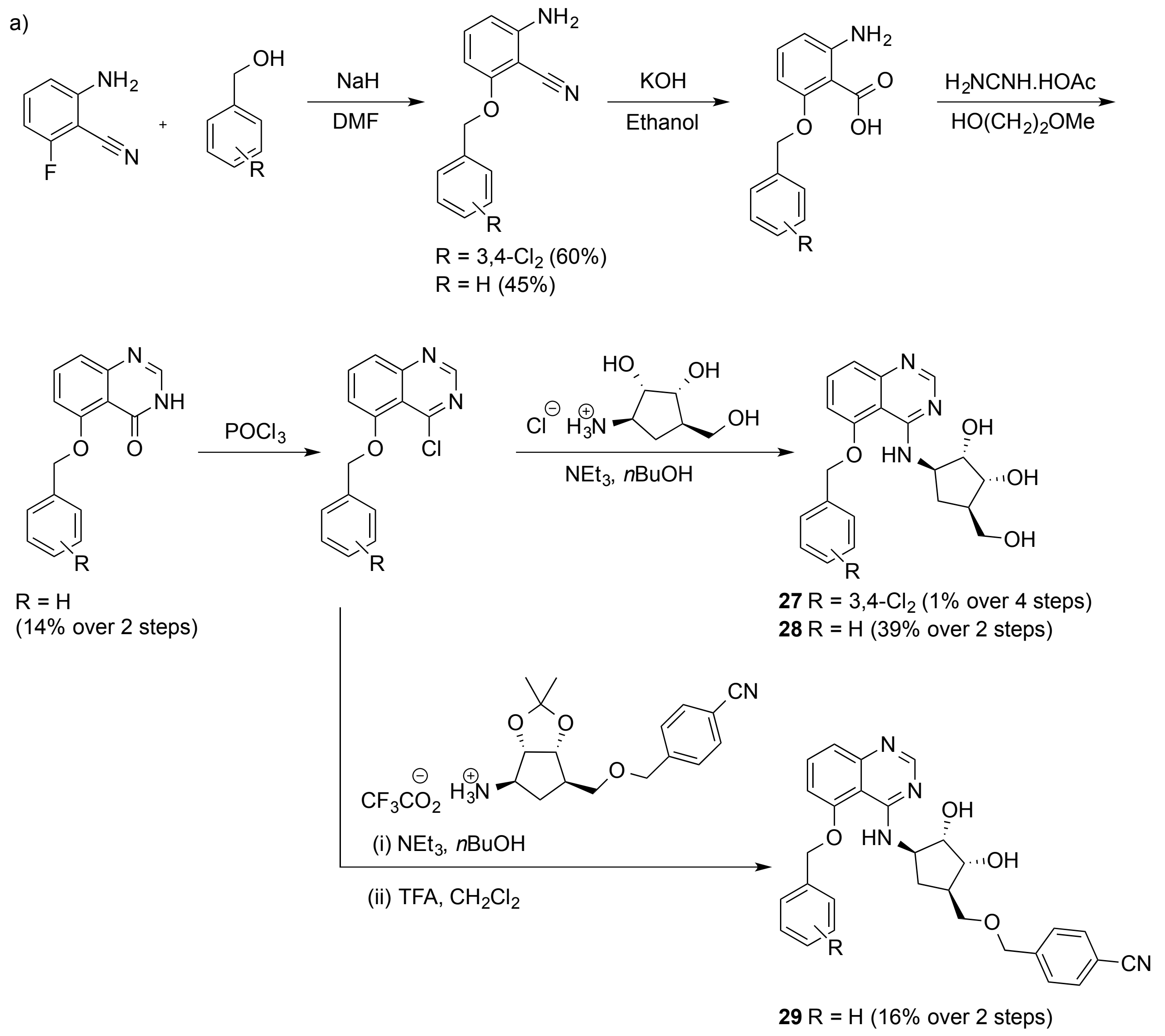

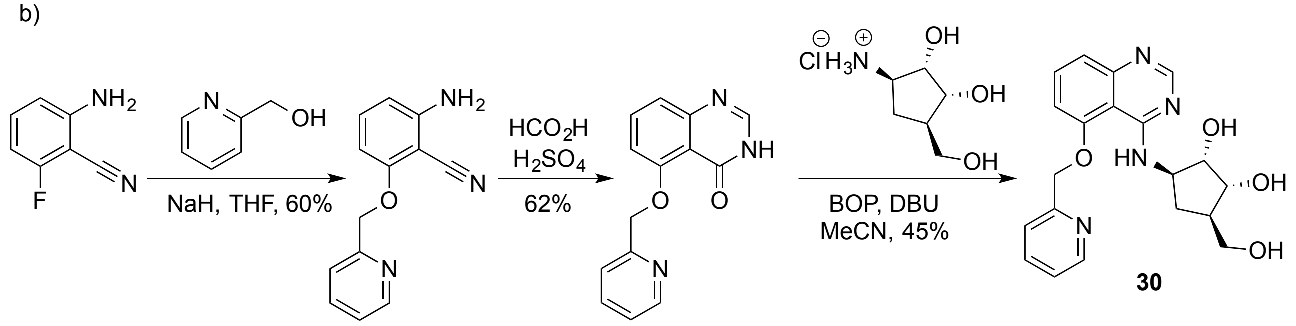

Figure S7. a) Synthetic route to 5-*O*-substituted 4-aminoquinazolines, b) Alternative synthetic route to pyridine **30**.

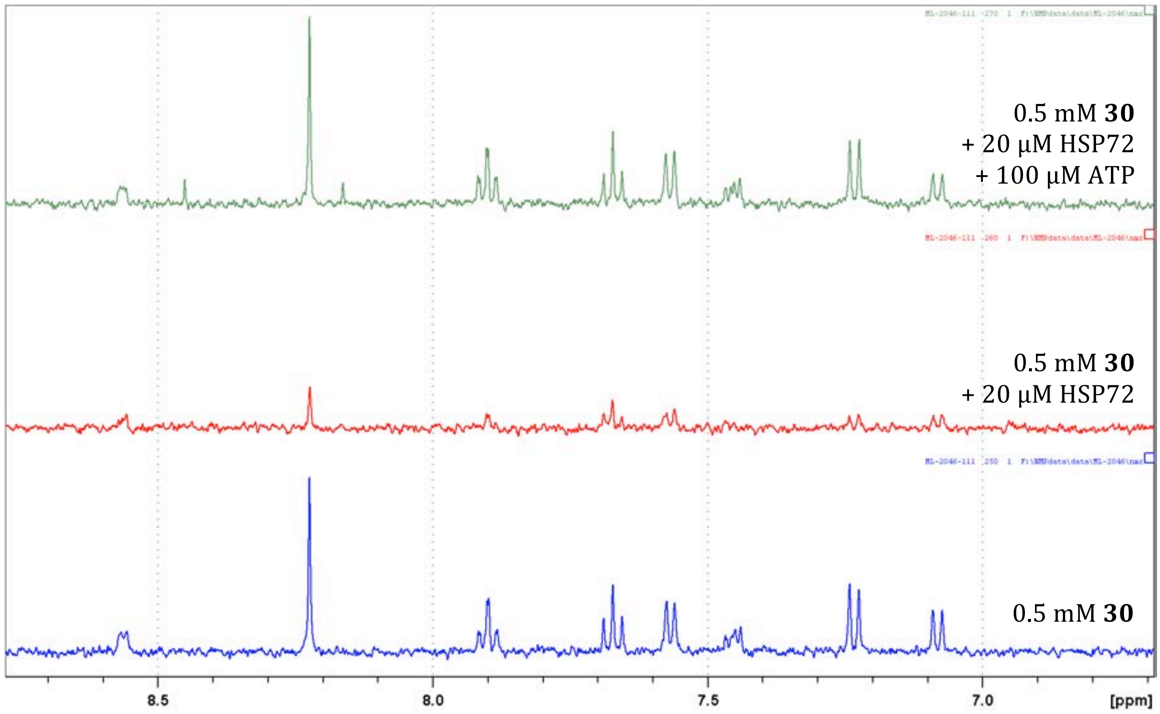

Figure S8. Ligand observed NMR data for compound binding of **30** to HSP72. 1D NMR spectrum of **30** in buffer is shown in blue. 1D CPMG NMR spectrum of **30** in the presence of HSP72 is shown in red. The reduction in peak size indicates binding of **30** to HSP72. The 1D CMPG NMR spectrum of **30** in the presence of HSP72 and ATP is shown in green. The increase in peak size as compared to the **30** in the presence of HSP72 and ATP indicates that **30** is competed off by ATP and binds in the ATP binding site as has been confirmed by X-ray crystallography for other compounds in this series.

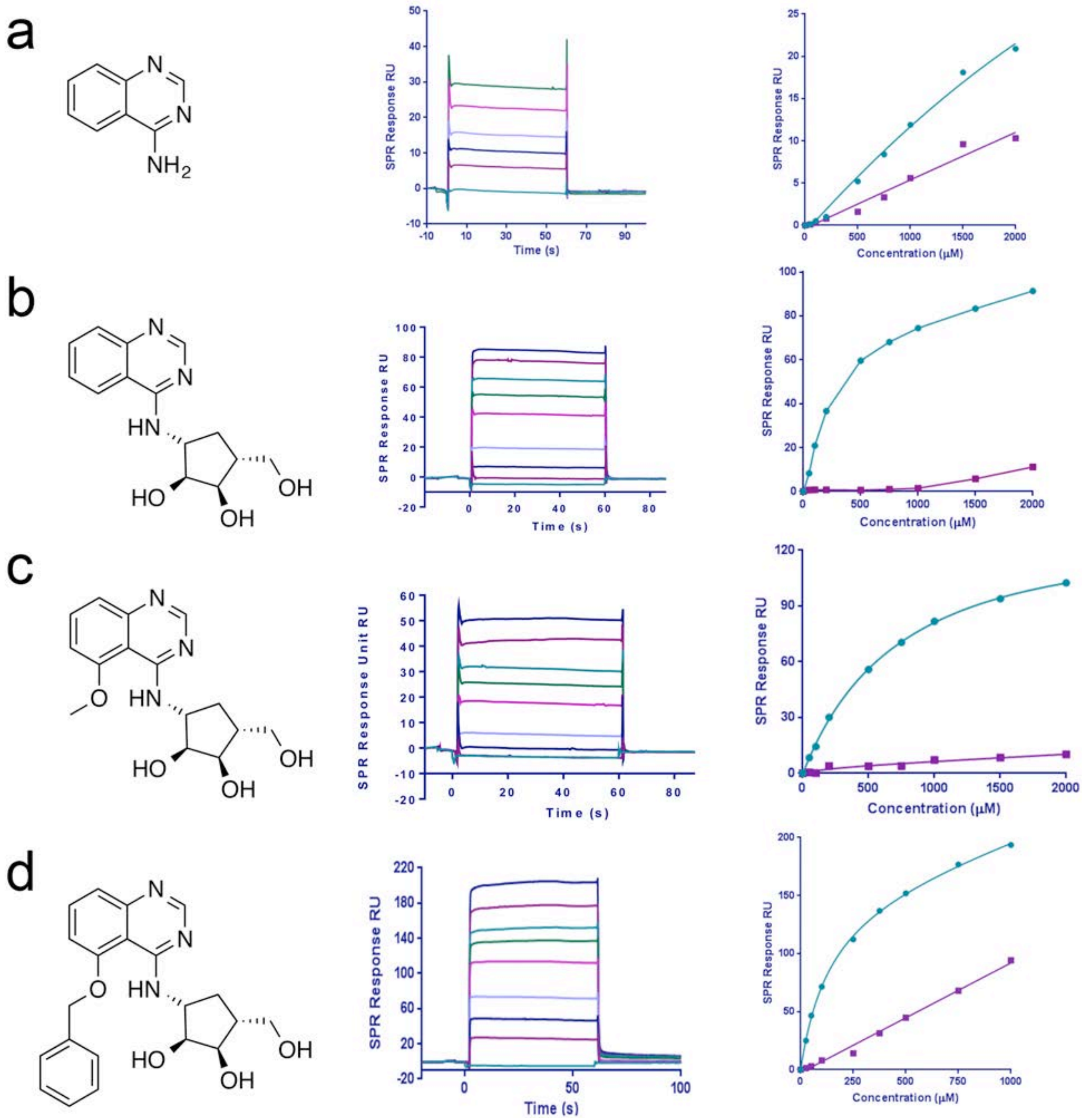

Figure S9. SPR data for key compounds. a) The unsubstituted aminoquinazoline **10**. b) The aminoquinazoline-cyclopentylaminotriol **23**. c) **26**, substitution at the quinazoline 5-position. d) **28,** further elaboration at the quinazoline 5-position. All panels show the chemical structure of the ligand, the SPR traces showing binding to wild-type HSC70NBD and the binding curves showing differential binding to wild-type HSC70-NBD and the S275W HSC70-NBD mutant.

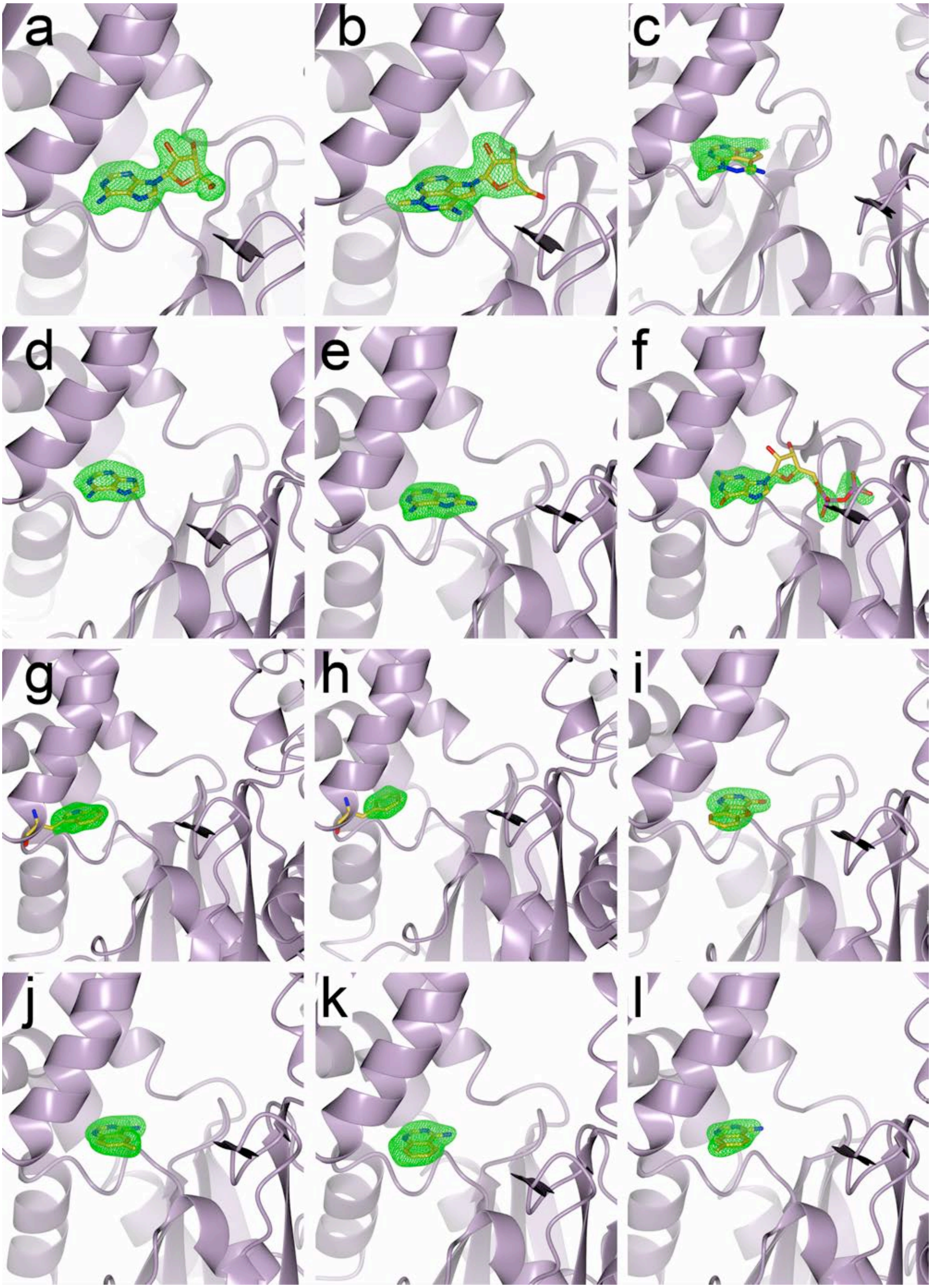
a
b
c
d
e
f
g
h
i
j
k
l

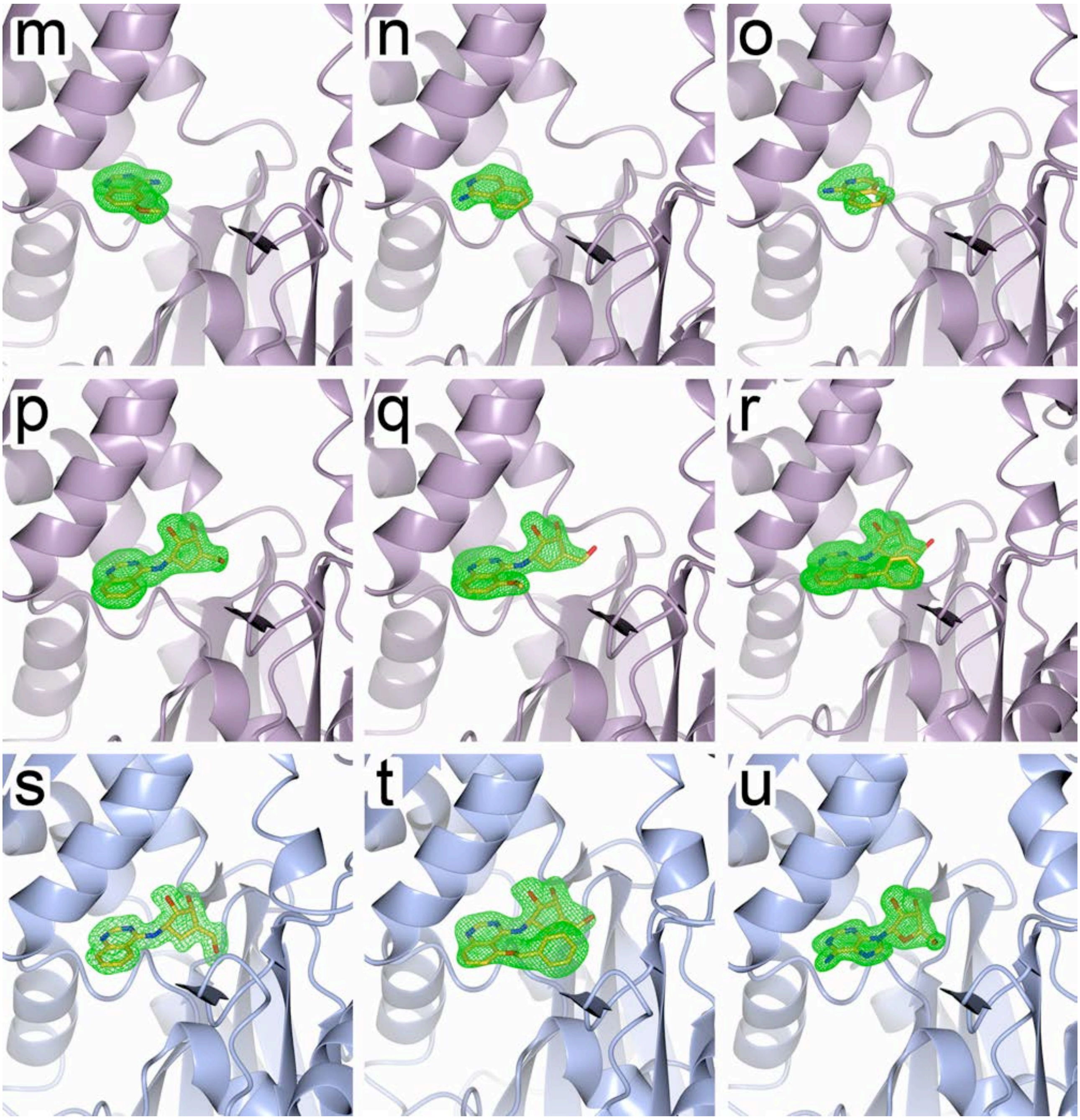

Figure S10. Fo-Fc electron density figures for ligand-bound structures and S275W/S275F HSC70-NBD mutants Fo-Fc electron density maps for HSC70/BAG1 ligand bound structures and ATP-site blocking mutants: a) adenosine, b) **2**, c) **3** d) adenine e) 8-aminoadenine f) GDP, g) S275W mutant h) S275F mutant i) **7**, j) **8**, k) **10**, l) **13**, m) **16**, n) **22** binding mode 1, o) **22** binding mode 2, p) **23**, q) **26**, r) **28**. Fo-Fc electron density maps for HSP72 ligand bound structures s) **23**, t) **28**, u) adenosine. All Fo-Fc maps are contoured at 3σ, except the Fo-Fc density for the triciribine fragment **3**, which was contoured at 2σ, reflecting its weak binding. Both proteins are shown in a ribbon representation with HSC70 in purple and HSP72 in blue.

Materials and Methods

Chemistry

General Experimental

Reactions were carried out under argon. Organic solutions were dried over Na_2SO_4 or $MgSO_4$. Starting materials and solvents were purchased from commercial suppliers and were used without further purification. Microwave reactions were carried out using a Biotage Initiator$^+$ microwave reactor. Flash silica chromatography was performed using Merck silica gel 60 (0.025-0.04 mm) or using Biotage SP4 automated column chromatographic equipment. ^{1}H NMR spectra were recorded on a Bruker AMX500 instrument at 500 MHz using internal deuterium locks. Chemical shifts (δ) are reported relative to TMS (δ = 0) and/or referenced to the solvent in which they were measured. Combined HPLC-MS analyses were recorded using an Agilent 6210 TOF HPLC-MS with a Phenomenex Gemini 3 µm C18 (3 cm x 4.6 mm i.d.) column, run at a temperature of 22 °C with gradient elution of 10-90% MeOH/0.1% aqueous formic acid at a flow rate of 1 mL/min and a run time of 3.5 min. UV detection was at 254 nm and ionisation was by positive or negative ion electrospray. Molecular weight scan range was 150-1000 amu. LC-MS CHROMASOLV solvents, formic acid, or alternative eluent modifiers were purchased from Sigma Aldrich (Poole, UK) unless otherwise stated. 500µL standard injections (with needle wash) of the sample were made onto a Phenomenex Gemini column (5µm, C18, 250 x 21.2 mm, Phenomenex, Torrance, USA). Chromatographic separation at room temperature was carried out using a Gilson GX-281 Liquid Handler system combined with a Gilson 322 HPLC pump (Gilson, Middleton, USA) over a 15 minute gradient elution from 60:40 to 70:30 methanol:water (both modified with 0.1% formic acid) at a flow rate of 20 mL/min. UV-Vis spectra were acquired at 254nm on a Gilson 156 UV-Vis detector (Gilson, Middleton, USA). Collection was triggered by UV signal, and collected using a Gilson GX-281 Liquid Handler system (Gilson, Middleton, USA). Raw data was processed using Gilson Trilution Software.

Compounds **8**, **10**, **19** and **22** were sourced commercially and used as supplied.

Preparation of compounds 3, 9, 11-18, 20, 21 and 23-30.

General Procedure A: Preparation of quinazoline-4(3*H*)-ones from anthranilic acids

A mixture of formamidine acetate (2.00 mmol) and the anthranilic acid (1.00 mmol) in 2-methoxyethanol (2.0 mL) was stirred at 120 °C for 12 h. The reaction mixture was cooled to room temperature and stirred for a further 30 min and the resulting solid was collected

by filtration. The solid was washed with 0.01 M aqueous ammonia (2 mL) and water (2 mL). The precipitate was dried under high vacuum to afford the quinazoline-4(3*H*)-one.

General Procedure B: Preparation of 4-chloroquinazolines from quinazoline-4(3*H*)-ones

To the quinazolin-4(3*H*)-one (0.57 mmol) was added phosphorus oxychloride (12.0 mmol) and triethylamine (2.02 mmol) and the reaction mixture was heated to reflux for 3 h. The solution was cooled to room temperature, excess reagents were removed by evaporation and the residue was dissolved in ethyl acetate (5 mL), washed with $NaHCO_3$ (aq.) (10 mL) and extracted with ethyl acetate (3 x 5 mL). The combined organic layers were combined, dried, filtered and evaporated to afford the 4-chloroquinazoline.

General Procedure C: Preparation of 4-aminoquinazolines from 4-chloroquinazolines

A sealed reaction vial, charged with a 4-chloroquinazoline derivative (0.36 mmol) and ammonium hydroxide (2.0 mL, 56.5 mmol), was heated to 115 ^oC for 30 min in a microwave reactor. The reaction mixture was cooled to 0 ^oC and the precipitate that formed was collected and washed with water (2 x 2 mL). The powder obtained was dried under high vacuum to afford the 4-aminoquinazoline. **CAUTION:** Gradual heating of the reaction mixture is advised to control pressure in the sealed system.

General Procedure D: Preparation of 4-aminoquinazolines from 2-aminobenzonitriles

Formamidine acetate (6.00 mmol) was added to the 2-aminobenzonitrile (1.00 mmol) in ethylene glycol monomethyl ether (2.0 mL) and the reaction mixture was refluxed under nitrogen for 5 h. The reaction mixture was cooled and water (1.0 mL) was added. The precipitate that formed was collected and washed with water (2.0 mL) and 0.01 M aqueous ammonia (2 mL). The material was dried under high vacuum to afford the 4-aminoquinazoline.

General Procedure E: Preparation of (1S,2R,3R,5R)-3-(hydroxymethyl)-5-(quinazolin-4-ylamino)cyclopentane-1,2-diols from 4-chloroquinazolines

Triethylamine (0.76 mmol) was added to a solution of (1*R*, 2*S*, 3*R*, 5*R*)-3-amino-5-(hydroxymethyl)cyclopentane-1,2-diol hydrochloride (0.30 mmol) in 1-butanol (1.5 mL) at room temperature and the solution was stirred for 5 min. The appropriate 4-chloroquinazoline derivative (0.30 mmol) was added and the reaction mixture was heated to 125 ^oC in a sealed vial in a microwave reactor for 35 min. The reaction mixture was

evaporated and the residue was purified by flash column chromatography on silica gel eluting with 0-20% methanol – dichloromethane. The product containing fractions were combined, concentrated and redissolved in methanol (10 mL). Ion exchange chromatography on SCX-2 acidic resin, eluting with 2M ammonia-methanol (15 mL), gave the corresponding substituted (1S,2R,3R,5R)-3-(hydroxymethyl)-5-(quinazolin-4-ylamino)cyclopentane-1,2-diol after evaporation of solvent.

General Procedure F: Preparation of 2-amino-6-alkoxybenzonitriles from 2-amino-6-fluorobenzonitrile

Sodium hydride (60% w/w dispersion in oil, 4.4 mmol) was added portion-wise over 10 min to the appropriate alcohol (4.4 mmol) in tetrahydrofuran (10 mL) at 0 ^{o}C. The solution was stirred at 0 ^{o}C for a further 1 h and then 2-amino-6-fluorobenzonitrile (0.40 g, 2.90 mmol) was added in one portion and the reaction mixture was heated at 85 ^{o}C for 2 h. The reaction mixture was cooled to room temperature, quenched with water (5 mL) and solvents were evaporated. The residue was dissolved in ethyl acetate (20 mL) and washed with water (2 x 20 mL) and brine (20 mL). The organic layer was dried, filtered and evaporated. The crude product was purified by flash column chromatography, eluting with a gradient of 0–10% 7M ammonia in methanol – dichloromethane, to give the corresponding 2-amino-6-alkoxybenzonitrile.

General Procedure G: Preparation of 2-amino-6-alkoxybenzoic acids from 2-amino-6-alkoxybenzonitriles

Potassium hydroxide (16 mmol) was added in one portion to the appropriate 2-amino-6-alkoxybenzonitrile (2.35 mmol) in ethanol (8 mL). The mixture was heated at reflux for 48 h, after which additional potassium hydroxide (16 mmol) was added and heating at reflux continued for a further 48 h. The mixture was cooled to room temperature, diluted with water (50 mL), and acidified to pH 1 with 2M hydrochloric acid. The product was extracted into ethyl acetate (3 x 50 mL) and the organic extracts were combined, dried, filtered and evaporated to afford the crude 2-amino-6-alkoxybenzoic acids which were used directly in the next step.

5-Methyl-1,5-dihydro-1,4,5,6,8-pentaazaacenaphthylen-3-amine (**3**)

Sodium hydride (15 mg, 0.67 mmol, 60%w/v dispersion in oil) was added to a solution of 4-chloro-7*H*-pyrrolo[2,3-*d*]pyrimidine-5-carbonitrile (0.10 g, 0.56 mmol) in anhydrous degassed DMF (5.6 mL) and the reaction mixture was stirred at room temperature for 15

min. SEM-chloride (0.12 mL, 0.67 mmol) was added dropwise and the reaction mixture was stirred at room temperature for 18 h. The reaction was quenched by addition of brine (10 mL) and extracted with ethyl acetate (3 x 30 mL). The combined organic extracts were dried, filtered and concentrated. Flash column chromatography on silica, eluting with a gradient of 0 – 20% ethyl acetate – petroleum ether, gave 4-chloro-7-((2-(trimethylsilyl)ethoxy)methyl)-7*H*-pyrrolo[2,3-*d*]pyrimidine-5-carbonitrile[1] (104 mg, 0.34 mmol, 60%) as a white solid. M.p. 259-260 °C; ^{1}H NMR (500 MHz, $CDCl_3$) δ 8.79 (s, 1H), 7.97 (s, 1H), 3.63 – 3.53 (m, 2H), 1.00 – 0.84 (m, 2H), -0.02 (s, 9H); ^{13}C NMR (125 MHz, $CDCl_3$) δ 153.3, 153.1, 151.2, 136.6, 115.9, 113.0, 86.8, 74.0, 67.8, 17.7, -1.46; LCMS *m/z* 309 (M+H). Methyl hydrazine (8.0 μL, 0.16 mmol) was added to a solution of 4-chloro-7-((2-(trimethylsilyl)ethoxy)methyl)-7*H*-pyrrolo[2,3-*d*]pyrimidine-5-carbonitrile (40 mg, 0.13 mmol) in ethanol (5 mL) and chloroform (2.5 mL) at room temperature and the reaction mixture stirred for 2 h. Solvent was removed by evaporation and the residue was dissolved in ethanol (5 mL). Concentrated hydrochloric acid (20 μL) was added and the reaction mixture was heated at reflux for 5 h. The reaction mixture was concentrated. Flash column chromatography on silica, eluting with a gradient of 4 – 20% methanol – chloroform, gave 5-methyl-1-((2-(trimethylsilyl)ethoxy)methyl)-1,5-dihydro-1,4,5,6,8-pentaazaacenaphthylen-3-amine (68 mg, 0.21 mmol, 73%) as a brown solid M.p. 89-90 °C; ^{1}H NMR (500 MHz, Methanol-d_4) δ 8.05 (s, 1H), 6.98 (s, 1H), 5.52 (s, 2H), 3.71 – 3.52 (m, 2H), 3.48 (s, 3H), 0.98 – 0.79 (m, 2H), -0.05 (s, 9H); ^{13}C NMR (126 MHz, MeOD) δ 155.6, 151.0, 146.7, 145.7, 111.3, 108.9, 103.8, 73.7, 66.2, 35.0, 17.2, -2.8; LC-MS (TOF, 4.0 min) Rt = 2.66 min; *m/z* (ESI) 319 (M+H); Hi-Res LC-MS (ESI) *m/z* calcd for $C_{14}H_{23}N_6OSi$ (M+H) 319.1697, found 319.1694 (-1.05 ppm). A mixture of 5-methyl-1-((2-(trimethylsilyl)ethoxy)methyl)-1,5-dihydro-1,4,5,6,8-pentaazaacenaphthylen-3-amine (34 mg, 0.11 mmol), tetrabutyl ammonium fluoride (1 M in THF, 0.90 mL, 0.89 mmol) and ethylene diamine (32 μL, 0.11 mmol) in DMF (2.4 mL) was stirred at 60 °C for 16 h. Brine (12 mL) was added and the mixture was washed with ethyl acetate (15 mL x 2). The aqueous layer was evaporated to dryness and the residue was purified by flash column chromatography on silica, eluting with a gradient of 10 – 20% methanol – chloroform, MeOH:$CHCl_3$, then by ion exchange chromatography on SCX-2 acidic resin, eluting with 0.2 M ammonia in methanol, to give (**3**) (9 mg, 0.05 mmol, 45%) as a pale grey solid M.p. 252-253 °C; ^{1}H NMR (500 MHz, DMSO-d_6) δ 11.77 (s, 1H), 7.98 (s, 1H), 6.85 (s, 1H), 6.13 (s, 2H), 3.37 (s, 3H); ^{13}C NMR (125 MHz, DMSO) δ 156.3, 151.9, 146.9, 146.7, 108.9, 108.4, 103.8, 35.5. LCMS *m/z* 189 (M+H); HRMS *m/z* calcd for $C_8H_9N_6$ (M+H) 189.0883, found 189.0884.

Benzofuro[3,2-*d*]pyrimidin-4-amine[2] (**9**)

Using general procedure C starting from benzofuro[3,2-d]pyrimidin-4(3H)-one afforded 4-chlorobenzofuro[3,2-*d*]pyrimidine[3] (0.39 g, 1.89 mmol, 94%) as a pink powder. M.p. 144-145 ^{o}C (lit. 135-138 ^{o}C, cyclohexane)[4]; ^{1}H NMR (500 MHz, $CDCl_3$) δ 9.01 (s, 1H), 8.27 (ddd, *J* = 7.9, 1.0, 1.0 Hz, 1H), 7.80-7.74 (m, 2H), 7.56 (ddd, *J* = 7.9, 6.7, 1.4 Hz, 1H); ^{13}C NMR (125 MHz, $CDCl_3$) δ 158.0, 153.0, 151.2, 144.4, 142.7, 132.6, 124.9, 122.7, 121.5, 113.1; LCMS *m/z* 205 $[M+H]^+$. Following general procedure D starting from 4-chlorobenzofuro[3,2-*d*]pyrimidine gave (**9**) (24 mg, 0.13 mmol, 53%) as a white powder. M.p. 267-268 ^{o}C (lit. 268-269 ^{o}C)[4]; ^{1}H NMR (500 MHz, DMSO-d_6) δ 8.41 (s, 1H), 8.09 – 8.07 (m, 1H), 7.80 – 7.77 (m, 1H), 7.69 (ddd, *J* = 8.3, 7.2, 1.4 Hz, 1H), 7.56 (br s, 2H), 7.49 (ddd, *J* = 8.3, 7.2, 1.4 Hz, 1H); ^{13}C NMR (125 MHz, DMSO-d_6) δ 156.1, 154.0, 150.8, 145.8, 134.8, 130.6, 124.4, 122.9, 121.8, 113.4; LCMS *m/z* 186 $[M+H]^+$.

2-Methylquinazolin-4-amine (**11**)

A saturated solution of KOH (0.119 g, 2.12 mmol) in ethanol (0.1 mL) was added to a mixture of anthranilonitrile (0.25 g, 2.12 mmol), acetonitrile (0.57 mL, 11 mmol) and ethanol (0.57 mL). The mixture was heated at 120 ^{o}C in a sealed glass tube for 44 h. The mixture was cooled to 0 ^{o}C and the resulting precipitate was collected and washed with 0.01 M aqueous ammonia, then with water. The solid was dried in high vacuum. Flash column chromatography on silica, eluting with a gradient of 1 – 10% MeOH – dichloromethane gave (**11**) (65 mg, 0.41 mmol, 19%). ^{1}H NMR (500 MHz, DMSO-d_6) δ 8.15 (dd, *J* = 8.2, 1.0 Hz, 1H), 7.71 – 7.65 (m, 3H), 7.57 (d, *J* = 8.2 Hz), 7.39 (1H, ddd, *J* = 8.2, 7.0, 1.0 Hz), 2.41 (s, 3H); ^{13}C NMR (125 MHz, DMSO-d_6) δ 164.1, 162.2, 150.7, 133.1, 127.2, 124.9, 123.9, 113.0, 26.3; LCMS *m/z* 160 $[M+H]^+$.

5-Methylquinazolin-4-amine (**12**)

Following general procedure D starting from 2-amino-6-methylbenzonitrile, gave (**12**) as a light brown solid (89 mg, 0.56 mmol, 56%). M.p. >270 ^{o}C; ^{1}H NMR (500 MHz, DMSO-d_6) δ 8.31 (s, 1H), 7.59 (dd, *J* = 8.3, 7.1 Hz, 1H), 7.49 (d, *J* = 8.3 Hz, 1H), 7.25 (d, *J* = 7.1 Hz, 1H), 7.18 (br s, 2H), 2.82 (s, 3H); ^{13}C NMR (125 MHz, DMSO-d_6) δ 162.9, 154.9, 152.2, 135.7, 132.5, 128.5, 126.1, 115.0, 23.8; LCMS *m/z* 161 $[M+H]^+$.

7-Methylquinazolin-4-amine (**13**)[5]

Following general procedure A starting from 4-methylanthranilic acid gave 7-methylquinazolin-4(3*H*)-one (73%). ^{1}H NMR (500 MHz, DMSO-d_6) δ 12.16 (br s, 1H), 8.06 (s, 1H), 8.01 (d, *J* = 8.0 Hz, 1H), 7.49 – 7.47 (m, 1H), 7.35 (dd, *J* = 8.0, 1.2 Hz, 1H), 2.26 (s, 3H); ^{13}C NMR (125 MHz, DMSO-d_6) δ 161.1, 149.3, 145.9, 145.3, 128.6, 127.3, 126.2, 120.7, 21.8; LCMS *m/z* 161 $[M+H]^+$. Applying general procedure B to 7-methylquinazolin-4(3*H*)-one gave 4-chloro-7-methylquinazoline (86%). ^{1}H NMR (500 MHz, $CDCl_3$) δ 9.02 (s, 1H), 8.17 (d, *J* = 8.6 Hz, 1H), 7.87 – 7.85 (m, 1H), 7.58 (dd, *J* = 8.6, 1,6 Hz, 1H), 2.64 (s, 3H); LCMS *m/z* 179 $[M+H]^+$. Applying general procedure C to 4-chloro-7-methylquinazoline gave (**13**) (0.11 g, 0.68 mmol, 68%) as a grey powder. M.p. 284-285 ^{o}C (Lit. 286-289 ^{o}C)[6]; ^{1}H NMR (500 MHz, DMSO-d_6) δ 8.33 (s, 1H), 8.09 (d, *J* = 8.4 Hz, 1H), 7.65 (s, 2H), 7.50 – 7.40 (m, 1H), 7.31 (dd, *J* = 8.5, 1.7 Hz, 1H), 2.45 (d, *J* = 0.9 Hz, 3H); ^{13}C NMR (125 MHz, DMSO-d_6) δ 162.0, 156.0, 150.3, 143.3, 127.6, 126.9, 123.8, 112.7, 21.8; LCMS *m/z* 161 $[M+H]^+$.

8-Methylquinazolin-4-amine (**14**)

Following general procedure A starting from 3-methylanthranilic acid gave 8-methylquinazolin-4(3*H*)-one (81%) as a brown powder. ^{1}H NMR (500 MHz, DMSO-d_6) δ 12.2 (br s, 1H), 8.11 (s, 1H), 7.96 (dd, *J* = 8.0, 1.0 Hz, 1H), 7.67 (ddd, *J* = 8, 1.5, 1.0 Hz, 1H), 7.40 (dd, *J* = 8, 8 Hz, 1H), 2.53 (s, 3H); ^{13}C NMR (125 MHz, DMSO-d_6) δ 161.5, 147.7, 144.9, 135.8, 135.2, 126.7, 124.0, 123.0, 17.7; LCMS *m/z* 161 $[M+H]^+$. Applying general procedure B to 8-methylquinazolin-4(3*H*)-one gave 4-chloro-8-methylquinazoline (81%). ^{1}H NMR (500 MHz, $CDCl_3$) δ 9.08 (s, 1H), 8.14 (dd, *J* = 8.5, 1.0 Hz, 1H), 7.82 (dd, *J* = 7.2, 1.0 Hz, 1H), 7.63 (dd, *J* = 8.5, 7.2 Hz, 1H), 2.80 (s, 3H); ^{13}C NMR (125 MHz, $CDCl_3$) δ 162.7, 152.7, 150.3, 137.4, 134.9, 128.6, 124.1, 123.6, 17.6; LCMS *m/z* 179 $[M+H]^+$. Applying general procedure C to 4-chloro-8-methylquinazoline gave (**14**) (28 mg, 0.18 mmol, 63%) as an off-white powder. ^{1}H NMR (500 MHz, DMSO-d_6) δ 8.41 (s, 1H), 8.03 (d, *J* = 8 Hz, 1H), 7.70 – 7.60 (m, 3H), 7.35 (dd, *J* = 8.2, 7.2 Hz, 1H), 2.55 (s, 3H); ^{13}C NMR (125 MHz, DMSO-d_6) δ 162.5, 155.1, 148.9, 135.6, 133.1, 125.2, 121.8, 114.5, 17.9; LCMS *m/z* 160 $[M+H]^+$.

5-Methoxyquinazolin-4-amine (**15**)

Following general procedure A starting from 6-methoxyanthranilic acid gave 5-methoxyquinazolin-4(3*H*)-one[7] (77%) as a yellow powder. M.p. 210-211 °C (lit. 208-209 °C)[8]; ^{1}H NMR (500 MHz, DMSO-d_6) δ 11.91 (br s, 1H), 7.95 (d, *J* = 3.2 Hz, 1H), 7.67 (dd, *J* = 8.2, 8.2 Hz, 1H), 7.16 (dd, *J* = 8.2, 1.2 Hz, 1H), 7.01 (d, *J* = 8.2 Hz, 1H), 3.85 (s, 3H); ^{13}C NMR (125 MHz, DMSO-d_6) δ 160.1, 159.3, 151.8, 146.2, 135.0, 119.5, 112.8, 108.8, 56.4; LCMS *m/z* 177 $[M+H]^+$. Applying general procedure B to 5-methoxyquinazolin-4(3*H*)-one gave 4-chloro-5-methoxyquinazoline[8] as a yellow powder (86%). M.p. 165-166 °C (lit. 113-114 °C)[8]; ^{1}H NMR (500 MHz, DMSO-d_6) δ 8.58 (s, 1H), 7.79 (dd, *J* = 8.4, 8.4 Hz, 1H), 7.27 (dd, *J* = 8.4, 1.0 Hz, 1H), 7.14 (d, *J* = 8.4 Hz, 1H), 3.89 (s, 3H); ^{13}C NMR (125 MHz, DMSO-d_6) δ 160.4, 158.2, 147.9, 136.0, 135.2, 115.7, 111.7, 110.0, 56.7; LCMS *m/z* 195 $[M+H]^+$. Applying general procedure C to 4-chloro-5-methoxyquinazoline gave (**15**) (22 mg, 0.13 mmol, 35%) as a yellow powder. M.p. 230-231 °C; ^{1}H NMR (500 MHz, DMSO) δ 8.28 (s, 1H), 7.76 (br s, 1H), 7.69 (br s, 1H), 7.63 (dd, *J* = 8.2 Hz, 1H), 7.20 (dd, *J* = 8.3, 1.0 Hz, 1H), 6.95 (dd, *J* = 8.2, 1.0 Hz, 1H), 3.97 (s, 3H); ^{13}C NMR (125 MHz, DMSO) δ 161.5, 157.4, 156.0, 152.5, 133.4, 119.8, 106.1, 105.8, 56.6; LCMS *m/z* 176 $[M+H]^+$.

6-Methoxyquinazoline-4-amine (**16**)

Following general procedure A starting from 5-methoxyanthranilic acid gave 6-methoxyquinazolin-4(3*H*)-one[9] (77%) as a white powder. M.p. 247-248 °C (dichloromethane), (lit. 238-240 °C, methanol)[10]; ^{1}H NMR (500 MHz, DMSO-d_6) δ 12.18 (s, 1H), 7.98 (s, 1H), 7.62 (d, *J* = 8.8 Hz, 1H), 7.50 (d, *J* = 3.0 Hz, 1H), 7.41 (dd, *J* = 8.8, 3.0 Hz, 1H), 3.87 (s, 3H); ^{13}C NMR (125 MHz, DMSO-d_6) δ 161.0, 158.2, 143.6, 129.4, 124.6, 124.2, 123.9, 106.3, 56.1; LCMS *m/z* 177 $[M+H]^+$. Applying general procedure B to 6-methoxyquinazolin-4(3*H*)-one gave 4-chloro-6-methoxyquinazoline[11] (92%) as a yellow powder. M.p. 202-203 °C (lit. 107.5-108.0 °C)[12]; ^{1}H NMR (500 MHz, $CDCl_3$) δ 8.95 (s, 1H), 7.99 (d, *J* = 9.1 Hz, 1H), 7.61 (dd, *J* = 9.1, 2.8 Hz, 1H), 7.45 (d, *J* = 2.8 Hz, 1H), 4.01 (s, 3H); ^{13}C NMR (125 MHz, $CDCl_3$) δ 160.6, 159.5, 151.6, 147.2, 130.3, 128.1, 125.2, 120.6, 56.0; LCMS *m/z* 195 $[M+H]^+$. Applying general procedure C to 4-chloro-6-methoxyquinazoline gave (**16**) (0.02 g, 0.11 mmol, 44%) as a white powder. M.p. 246-247 °C; ^{1}H NMR (500 MHz, DMSO-d_6) δ 8.28 (s, 1H), 7.63-7.56 (m, 4H), 7.39 (dd, *J* = 9.1, 2.8 Hz, 1H), 3.86 (s, 3H); ^{13}C NMR (125 MHz, DMSO-d_6) δ 161.5, 157.1, 153.8, 145.4, 129.4, 124.3, 115.3, 103.3, 56.2; LCMS *m/z* 176 $[M+H]^+$.

7-Methoxyquinazoline-4-amine (**17**)[13]

Following general procedure A starting from 4-methoxyanthranilic acid gave 7-methoxyquinazolin-4(3*H*)-one[9] (85%) as a white powder. M.p. 255-256 °C (lit. 257-258 °C)[14]; ^{1}H NMR (500 MHz, DMSO-d_6) δ 12.20 (s, 1H), 7.99 (s, 1H), 7.63 (d, *J* = 8.9 Hz, 1H), 7.51 (d, *J* = 3.0 Hz, 1H), 7.42 (dd, *J* = 8.9, 3.0 Hz, 1H), 3.88 (s, 3H); ^{13}C NMR (125 MHz, DMSO-d_6) δ 161.0, 158.2, 143.7, 143.6, 129.4, 124.2, 123.9, 106.3, 56.1; LCMS *m/z* 177 $[M+H]^+$. Applying general procedure B to 7-methoxyquinazolin-4(3*H*)-one gave 4-chloro-7-methoxyquinazoline[15] (97%) as a yellow powder. M.p. 255-256 °C (lit. 141-142 °C)[14]; ^{1}H NMR (500 MHz, $CDCl_3$) δ 8.96 (s, 1H), 8.17 (d, *J* = 9.1 Hz, 1H), 7.49 – 7.31 (m, 2H), 4.02 (s, 3H); ^{13}C NMR (125 MHz, $CDCl_3$) δ 165.1, 161.6, 154.1, 153.2, 127.2, 122.4, 119.2, 106.1, 56.1; LCMS *m/z* 195 $[M+H]^+$. Applying general procedure C to 4-chloro-7-methoxyquinazoline gave (**17**) (29 mg, 0.17 mmol, 64%) as a white powder. M.p. 267-268 °C; ^{1}H NMR (500 MHz, DMSO-d_6) δ 8.31 (s, 1H), 8.11 (d, *J* = 9.0 Hz, 1H), 7.57 (br s, 2H), 7.08 (dd, *J* = 9.0, 2.5 Hz, 1H), 7.05 (d, *J* = 2.5 Hz, 1H), 3.88 (s, 3H); ^{13}C NMR (125 MHz, DMSO-d_6) δ 161.8, 156.4, 152.4, 138.8, 134.7, 125.6, 117.1, 109.1, 55.9; LCMS *m/z* 176 $[M+H]^+$.

8-Methoxyquinazoline-4-amine (**18**)

Following general procedure A starting from 3-methoxyanthranilic acid gave 8-methoxyquinazolin-4(3*H*)-one (83%) as a brown powder. ^{1}H NMR (500 MHz, DMSO-d_6) δ 12.3 (br s, 1H), 8.03 (s, 1H), 7.66 (dd, *J* = 8, 1.4 Hz, 1H), 7.44 (dd, *J* = 8, 8 Hz, 1H), 7.35 (dd, *J* = 8, 1.2 Hz, 1H), 3.89 (s, 3H); ^{13}C NMR (125 MHz, DMSO-d_6) δ 161.1, 155.0, 144.4, 139.7, 127.5, 124.2, 117.3, 115.4, 56.4; LCMS *m/z* 177 $[M+H]^+$. Applying general procedure B to 8-methoxyquinazolin-4(3*H*)-one gave 4-chloro-8-methoxyquinazoline (54%). ^{1}H NMR (500 MHz, $CDCl_3$) δ 9.03 (s, 1H), 7.86 (dd, *J* = 8, 1 Hz, 1H), 7.68 (dd, *J* = 8, 8 Hz, 1H), 7.32 (dd, *J* = 8, 1 Hz, 1H), 4.13 (s, 3H); LCMS *m/z* 195 $[M+H]^+$. Applying general procedure C to 4-chloro-8-methoxyquinazoline gave (**18**) (21 mg, 0.12 mmol, 47%). ^{1}H NMR (500 MHz, DMSO-d_6) δ 8.34 (s, 1H), 7.71 (d, *J* = 8 Hz, 1H), 7.65 (br s, 2H), 7.38 (dd, *J* = 8, 8 Hz, 1H), 7.22 (d, *J* = 8 Hz, 1H), 3.89 (s, 3H); ^{13}C NMR (125 MHz, DMSO-d_6) δ 162.0, 154.9, 154.7, 141.9, 125.8, 115.4, 115.1, 112.3, 56.1; LCMS *m/z* 176 $[M+H]^+$.

N-Methylquinazolin-4-amine (**20**)

A solution of methylamine in THF (2M, 0.18 mL, 0.36 mmol) was added to a solution of 4-chloroquinazoline (30 mg, 0.18 mmol) in THF (2 mL). The mixture was stirred at r.t. for 18

h then concentrated. The residue was subject to ion exchange chromatography on acidic SCX-2 resin, eluting with 2M ammonia in MeOH, followed by flash column chromatography on silica, eluting with 5% methanol – dichloromethane, to give (**20**) (27 mg, 0.17 mmol, 93%)

^{1}H NMR (500 MHz, DMSO-d_6) δ 8.48 (s, 1H), 8.32 (br s, 1H), 8.17 (dd, *J* = 8.3, 1 Hz, 1H), 7.75 (ddd, J = 8.3, 7, 1 Hz, 1H), 7.67 (dd, *J* = 8.3, 1 Hz), 7.50 (ddd, *J* = 8.3, 7, 1 Hz, 1H), 3.00 (s, 3H); ^{13}C NMR (125 MHz, DMSO-d_6) δ 160.3, 155.6, 149.2, 132.9, 127.8, 126.0, 123.0, 115.5, 28.2; LCMS *m/z* 160 [M+H]$^+$.

N,N-Dimethylquinazolin-4-amine (**21**)

A solution of dimethylamine in THF (2M, 0.18 mL, 0.36 mmol) was added to a solution of 4-chloroquinazoline (30 mg, 0.18 mmol) in THF (2 mL). The mixture was stirred at r.t. for 18 h then concentrated. The residue was subject to ion exchange chromatography on acidic SCX-2 resin, eluting with 2M ammonia in MeOH, followed by flash column chromatography on silica, eluting with a gradient of 3 – 4% methanol – dichloromethane, to give (**21**) (31 mg, 0.18 mmol, 98%).

^{1}H NMR (500 MHz, $CDCl_3$) δ 8.66 (s, 1H), 8.05 (d, *J* = 8 Hz, 1H), 7.90 (d, *J* = 8 Hz, 1H), 7.73 (dd, *J* =8, 8 Hz, 1H), 7.43 (dd, *J* = 8, 8 Hz, 1H), 3.40 (s, 6H); ^{13}C NMR (125 MHz, $CDCl_3$) δ 153.7, 132.3, 128.1, 125.6, 124.7, 116.0, 41.9 (2 quaternary carbon not observed); LCMS *m/z* 174 [M+H]$^+$.

(1*S*,2*R*,3*R*,5*R*)-3-(Hydroxymethyl)-5-(quinazolin-4-ylamino)cyclopentane-1,2-diol (**23**)

Following general procedure A starting from anthranilic acid gave, quinazolin-4(3*H*)-one[16] as a white powder (74%). M.p. 215-216 °C (lit. 225-227 °C)[17]; ^{1}H NMR (500 MHz, DMSO-d_6) δ 7.43 (dd, *J* = 7.9, 1.3 Hz, 1H), 7.30 (s, 1H), 7.06-7.02 (m, 1H), 6.91 (d, *J* = 8.3 Hz, 1H), 6.78 – 6.74 (m, 1H); ^{13}C NMR (125 MHz, DMSO-d_6) δ 161.2, 147.5, 144.2, 133.7, 126.2, 125.6, 125.1, 121.6; LCMS *m/z* 147 [M+H]$^+$. Applying general procedure B to quinazolin-4(3*H*)-one gave 4-chloroquinazoline[18] as a yellow powder (64%). M.p. 256-257 °C (lit. 97-98 °C)[19]. ^{1}H NMR (500 MHz, $CDCl_3$) δ 9.06 (s, 1H), 8.29 – 8.26 (m, 1H), 8.09 – 8.07 (m, 1H), 7.98 (ddd, *J* = 8.6, 7.0, 1.3 Hz, 1H), 7.75 (ddd, *J* = 8.6, 7.0, 1.3 Hz, 1H); ^{13}C NMR (125 MHz, $CDCl_3$) δ 162.5, 153.7, 151.1, 135.0, 129.1, 128.8, 125.8, 124.0; LCMS *m/z* 165 [M+H]$^+$. Applying general procedure E to 4-chloroquinazoline gave (**23**) (29 mg, 0.11 mmol, 35%) as a white powder. M.p. 237-238 °C; ^{1}H NMR (500 MHz, DMSO-d_6) δ

8.44 (s, 1H), 8.27 (dd, *J* = 8.3, 1.3 Hz, 1H), 8.05 (d, *J* = 7.4 Hz, 1H), 7.75 (ddd, *J* = 8.3, 6.9, 1.3 Hz, 1H), 7.67 (dd, *J* = 8.4, 1.2 Hz, 1H), 7.50 (ddd, *J* = 8.3, 6.9, 1.3 Hz, 1H), 4.72 (t, *J* = 5.0 Hz, 1H), 4.69 (d, *J* = 5.6 Hz, 1H), 4.56 (qt, *J* = 7.7 Hz, 1H), 4.44 (d, *J* = 4.8 Hz, 1H), 3.89 (dt, *J* = 7.1, 5.3 Hz, 1H), 3.77 (q, *J* = 4.8 Hz, 1H), 3.44 (qt, *J* = 10.5, 5.4 Hz, 2H), 2.22 (dt, *J* = 13.0, 8.4 Hz, 1H), 2.08 – 1.82 (m, 1H), 1.23 (dt, *J* = 13.0, 8.4 Hz, 1H); ^{13}C NMR (125 MHz, DMSO) δ 159.9, 155.5, 149.6, 132.9, 127.9, 125.9, 123.2, 115.4, 76.2, 72.6, 63.4, 56.1, 45.7, 30.4; LCMS *m/z* 276 $[M+H]^+$; HRMS *m/z* calcd for $C_{14}H_{18}N_3O_3$ (M+H) 276.1343, found 276.1346.

(1*S*,2*R*,3*R*,5*R*)-3-(Hydroxymethyl)-5-(6,7-dimethoxyquinazolin-4-ylamino)cyclopentane-1,2-diol (**24**)

Following general procedure E starting from 4-chloro-6,7-dimethoxyquinazoline gave (**24**) (68 mg, 0.20 mmol, 75%) as a white powder M.p. 131-132 °C; ^{1}H NMR (500 MHz, DMSO-*d*$_6$) δ 8.34 (s, 1H), 7.82 (s, 1H), 7.62 (s, 1H), 7.08 (s, 1H), 4.82 – 4.69 (m, 2H), 4.54 (q, *J* = 8.1 Hz, 1H), 4.45 (d, *J* = 4.7 Hz, 1H), 3.92 – 3.85 (m, 7H), 3.77 (q, *J* = 4.8 Hz, 1H), 3.50 – 3.40 (m, 2H), 2.21 (dt, *J* = 12.8, 8.4 Hz, 1H), 2.02 – 1.87 (m, 1H), 1.31 – 1.21 (m, 1H); ^{13}C NMR (125 MHz, DMSO-*d*$_6$) δ 158.9, 153.6, 108.8, 107.4, 102.6, 76.3, 72.5, 63.4, 56.6, 56.2, 45.9, 30.4 (3 quaternary C not observed); LCMS *m/z* 336 $[M+H]^+$; HRMS *m/z* calcd for $C_{16}H_{22}N_3O_5$ (M+H) 337.1584, found 337.1582.

(1*S*,2*R*,3*R*,5*R*)-3-(Hydroxymethyl)-5-(methyl(quinazolin-4-yl)amino)cyclopentane-1,2-diol (**25**)

A mixture of 4-chloroquinazoline (0.80 g, 4.86 mmol), ((1*S*, 4*R*)-4-aminocyclopent-2-enyl)methanol (0.50 g, 4.42 mmol) and triethylamine (0.93 mL, 6.63 mmol) in 1-butanol (4.4 mL) was heated in a microwave reactor at 125 °C for 1 h. The reaction mixture was cooled to room temperature, concentrated and subject to flash column chromatography on silica, eluting with 10% ethanol - dichloromethane, to give crude ((1S,4R)-4-(quinazolin-4-ylamino)cyclopent-2-en-1-yl)methanol (0.86 g) as a yellow powder used directly in the next step. LCMS *m/z* 242 $[M+H]^+$. *tert*-Butyldimethylsilyl chloride (1.26 mL, 7.29 mmol) was added to a stirred solution of ((1*S*,4*R*)-4-(quinazolin-4-ylamino)cyclopent-2-en-1-yl)methanol (0.80 g, 3.32 mmol), imidazole (1.13 mL, 8.29 mmol) and DMAP (4.0 mg, 0.03 mmol) in anhydrous DMF (13 mL). The reaction mixture was stirred at room temperature for 12 h. The reaction was quenched with water (30 mL) and extracted with diethyl ether

(15 mL x 3). The combined extracts were washed with brine (15 mL), dried, filtered and concentrated. Flash column chromatography on silica, eluting with 20 – 50% ethyl acetate – petroleum ether, gave *N*-((*1R*,*4S*)-4-(((*tert*-butyldimethylsilyl)oxy)methyl)cyclopent-2-en-1-yl)quinazolin-4-amine (0.42 g, 1.18 mmol, 29% over 2 steps) as a brown oil. ^{1}H NMR (500 MHz, $CDCl_3$) δ 8.64 (s, 1H), 7.89 – 7.75 (m, 1H), 7.75 – 7.59 (m, 2H), 7.39 (ddd, *J* = 8.2, 7.0, 1.2 Hz, 1H), 6.07 (d, *J* = 8.2 Hz, 1H), 5.95 – 5.83 (m, 2H), 5.47 (tdq, *J* = 8.2, 5.0, 1.7 Hz, 1H), 3.68 – 3.53 (m, 2H), 2.89 (ddtq, *J* = 8.7, 5.2, 3.3, 1.7 Hz, 1H), 2.65 (dt, *J* = 13.7, 8.5 Hz, 1H), 1.46 (dt, *J* = 13.7, 5.0 Hz, 1H), 0.87 (s, 9H), 0.03 (d, *J* = 7.3 Hz, 6H); ^{13}C NMR (125 MHz, $CDCl_3$) δ 158.7, 155.4, 149.4, 136.2, 132.4, 132.0, 128.3, 125.7, 120.8, 114.9, 66.4, 56.4, 47.4, 34.7, 26.0, 18.5; LCMS *m/z* 356 $[M+H]^+$. Sodium hydride (60% w/w in oil; 34 mg, 0.90 mmol) was added to a solution of *N*-((*1R*,*4S*)-4-(((*tert*-butyldimethylsilyl)oxy)methyl)cyclopent-2-en-1-yl)quinazolin-4-amine (0.16 g, 0.45 mmol) in THF (6.4 mL) and DMF (1.6 mL) and the reaction mixture was stirred at room temperature for 15 min. Methyl iodide (0.056 mL, 0.90 mmol) was added and the resulting mixture was stirred at room temperature for 12 h. The reaction was quenched with saturated aqueous NH_4Cl (10 mL) and stirred for 30 min. The mixture was extracted with ethyl acetate (20 mL x 3). The combined extracts were washed with brine (10 mL), dried, filtered and concentrated. Flash column chromatography on KP-NH silica, eluting with 10 – 100% ethyl acetate – cyclohexane, gave *N*-((*1R*,*4S*)-4-(((*tert*-butyldimethylsilyl)oxy)methyl)cyclopent-2-en-1-yl)-N-methylquinazolin-4-amine (60 mg, 0.16 mmol, 36%) as a brown oil ^{1}H NMR (500 MHz, $CDCl_3$) δ 8.67 (s, 1H), 8.02 (dd, J = 8.5, 1.3 Hz, 1H), 7.92 – 7.79 (m, 1H), 7.70 (ddd, J = 8.3, 6.9, 1.4 Hz, 1H), 7.41 (ddd, J = 8.4, 7.0, 1.4 Hz, 1H), 5.96 (dt, J = 5.7, 2.2 Hz, 1H), 5.81 (dt, J = 5.7, 2.2 Hz, 1H), 5.70 (ddq, J = 9.3, 6.9, 2.2 Hz, 1H), 3.74 (dd, J = 9.9, 5.5 Hz, 1H), 3.70 – 3.64 (m, 1H), 3.17 (s, 3H), 2.96 – 2.87 (m, 1H), 2.62 (dt, J = 13.7, 8.5 Hz, 1H), 1.71 (dt, J = 13.6, 7.3 Hz, 1H), 0.92 (s, 9H), 0.08 (s, 6H); ^{13}C NMR (125 MHz, CDCl3) δ 163.8, 154.0, 152.0, 136.5, 132.0 (x2), 128.3, 125.4, 124.5, 116.4, 66.9, 66.0, 47.3, 34.3, 30.3, 29.7, 25.9, (Me_3Si signal off scale); LCMS *m/z* 370 $[M+H]^+$. *N*-((1*R*,4*S*)-4-(((*tert*-butyldimethylsilyl)oxy)methyl)cyclopent-2-en-1-yl)-*N*-methylquinazolin-4-amine (30 mg, 0.08 mmol) was dissolved in water (2 mL) and acetone (2 mL). Potassium osmate (0.9 mg, 2.4 μmol) pre-dissolved in water (1 mL), citric acid (12 mg, 0.06 mmol) and *N*-methylmorpholine *N*-oxide (19 mg, 0.16 mmol) were added and the reaction was stirred for 15 h. Sodium sulphite (0.5 g) was added and the mixture was stirred for a further 30 min. Water (10 mL) was added and the mixture was extracted with ethyl acetate (3 x 10 mL). The combined extracts were dried, filtered and concentrated to give crude (1*S*,2*R*,3*R*,5*R*)-3-(((*tert*-butyldimethylsilyl)oxy)methyl)-5-

(methyl(quinazolin-4-yl)amino)cyclopentane-1,2-diol as a yellow oil which was used in the next step without purification. LCMS *m/z* 404 $[M+H]^+$. Hydrochloric acid (30% in water, 3 mL) was added dropwise to a solution of crude (1*S*,2*R*,3*R*,5*R*)-3-(((*tert*-butyldimethylsilyl)oxy)methyl)-5-(methyl(quinazolin-4-yl)amino)cyclopentane-1,2-diol (30 mg, 0.07 mmol) in methanol (3 mL). The mixture was stirred at room temperature for 3 h then concentrated. The residue was partitioned between water (10 mL) and ethyl acetate (10 mL x 3). The aqueous layer was concentrated, then re-dissolved in methanol (10 mL) and subject to ion exchange chromatography on SCX-2 acidic resin, eluting with 2M ammonia in methanol, to give (**25**) (8 mg, 0.03 mmol, 37% over 2 steps) as a colourless oil. ^{1}H NMR (500 MHz, CD_3OD) δ 8.60 (s, 1H), 8.42 – 8.34 (m, 1H), 7.93 (ddd, *J* = 8.4, 7.1, 1.2 Hz, 1H), 7.81 (dd, *J* = 8.5, 1.2 Hz, 1H), 7.67 (ddd, *J* = 8.5, 7.1, 1.3 Hz, 1H), 5.27 – 5.09 (m, 1H), 4.31 (dd, *J* = 9.3, 5.5 Hz, 1H), 3.94 (dd, *J* = 5.5, 2.7 Hz, 1H), 3.66 (h, *J* = 5.5 Hz, 2H), 3.47 (s, 3H), 2.31 (dt, *J* = 13.0, 8.5 Hz, 1H), 2.19 (ttd, *J* = 8.6, 5.7, 2.8 Hz, 1H), 1.71 (ddd, *J* = 13.1, 10.8, 7.9 Hz, 1H); ^{13}C NMR (125 MHz, CD_3OD) δ 164.5, 150.0, 134.2, 126.8, 126.1, 122.2, 114.3, 73.0, 72.3, 65.4, 63.1, 44.6, 33.9, 24.9, one quaternary carbon not observed; LCMS *m/z* 290 $[M+H]^+$; HRMS *m/z* calcd for $C_{15}H_{20}N_3O_3$ (M+H) 290.1499, found 290.1497.

(1*S*,2*R*,3*R*,5*R*)-3-(Hydroxymethyl)-5-(5-methoxyquinazolin-4-ylamino)cyclopentane-1,2-diol (**26**)

Following general procedure E starting from 5-methoxy-4-chloroquinazoline gave (**26**) (5.0 mg, 0.016 mmol, 3%) as a colourless film. ^{1}H NMR (500 MHz, CD_3OD) δ 8.39 (s, 1H), 7.72 (dd, *J* = 8.2, 8.2 Hz, 1H), 7.27 (dd, *J* = 8.2, 0.6 Hz, 1H), 7.08 (d, *J* = 8.2 Hz, 1H), 4.60 – 4.55 (m, 1H), 4.08 (s, 3H), 4.03 (t, *J* = 5.3 Hz, 1H), 3.99 (t, *J* = 5.3 Hz, 1H), 3.73 – 3.65 (m, 2H), 2.60 – 2.53 (m, 1H), 2.24 – 2.17 (m, 1H), 1.47 (qt, *J* = 6.7 Hz, 1H); ^{13}C NMR (125 MHz, CD_3OD) δ 159.9, 157.3, 154.0, 148.7, 133.7, 117.2, 106.5, 105.9, 77.2, 73.0, 62.0, 56.4, 55.7, 45.0, 29.5; LCMS *m/z* 306 $[M+H]^+$; HRMS *m/z* calcd for $C_{15}H_{20}N_3O_4$ (M+H) 307.1478, found 307.1471.

(1*R*,2*S*,3*R*,5*R*)-3-(5-(3,4-Dichlorobenzyloxy)quinazolin-4-ylamino)-5-(hydroxymethyl)cyclopentane-1,2-diol (**27**)

Following general procedure F starting from 3,4-dichlorobenzyl alcohol gave 2-amino-6-((3,4-dichlorobenzyl)oxy)benzonitrile (0.77 g, 2.6 mmol, 60%) as a white powder M.p. 139-

140 °C; ^{1}H NMR (500 MHz, $CDCl_3$) δ 7.54 (dd, *J* = 1.9, 0.9 Hz, 1H), 7.51 – 7.48 (m, 1H), 7.36 – 7.31 (m, 1H), 7.25 – 7.20 (m, 1H), 6.37 (dd, *J* = 8.4, 0.7 Hz, 1H), 6.24 (dd, *J* = 8.3, 0.7 Hz, 1H), 5.11 (t, *J* = 0.8 Hz, 2H); LCMS *m/z* 293 $[M+H]^+$. Using general procedure G, 2-amino-6-((3,4-dichlorobenzyl)oxy)benzonitrile (0.69 g, 2.35 mmol) was converted to crude 2-amino-6-((3,4-dichlorobenzyl)oxy)benzoic acid as a black powder (0.70 g) which was used directly in the next step. LCMS *m/z* 310 $[M+H]^+$. Following general procedure A, 5-((3,4-dichlorobenzyl)oxy)quinazolin-4(3H)-one was afforded as a black powder (0.52 g) used directly in the next step. Following general procedure C, 4-chloro-5-((3,4-dichlorobenzyl)oxy)quinazoline was afforded as a black powder (0.37 g) used directly in the following step. LCMS *m/z* 339 $[M+H]^+$. Following general procedure E, (**27**) (4.0 mg, 0.009 mmol, 1% over 4 steps) was obtained as a clear oil after semi-preparative HPLC purification. ^{1}H NMR (500 MHz, CD_3OD) δ 8.42 (br s, 1H), 8.40 (s, 1H), 7.80 (d, *J* = 2.0 Hz, 1H), 7.72 (dd, *J* = 8.3, 8.3 Hz, 1H), 7.66 (d, *J* = 8.3 Hz, 1H), 7.54 (dd, *J* = 8.3, 2.0 Hz, 1H), 7.33 (d, *J* = 8.4 Hz, 1H), 7.16 (d, *J* = 8.2 Hz, 1H), 5.36 (s, 2H), 4.57 – 4.51 (m, 1H), 3.86 (t, *J* = 4.9, 4.9 Hz, 1H), 3.72 – 3.69 (m, 1H), 3.52 (d, *J* = 5.8 Hz, 1H), 2.46 – 2.38 (m, 1H), 2.19-2.12 (m, 1H), 1.05 – 0.98 (m, 1H); ^{13}C NMR (125 MHz, CD_3OD) δ 160.3, 155.8, 154.3, 149.8, 136.4, 133.4, 132.52, 132.50, 131.0, 130.2, 128.0, 118.4, 107.9, 106.1, 77.5, 73.0, 70.0, 63.1, 56.3, 45.2, 29.9; LCMS *m/z* 450 $[M+H]^+$; HRMS *m/z* calcd for $C_{21}H_{22}Cl_2N_3O_4$ (M+H) 450.0982, found 450.0975.

(1*R*,2*S*,3*R*,5*R*)-3-(5-(Benzyloxy)quinazolin-4-ylamino)-5-(hydroxymethyl)-cyclopentane-1,2-diol (**28**)

Following general procedure F starting from benzyl alcohol gave 2-amino-6-(benzyloxy)benzonitrile[20] as yellow crystals (1.39 g, 6.18 mmol, 67%). M.p. 115-116 °C (lit. 99-100 °C, EtOH)[20]; ^{1}H NMR (500 MHz, $CDCl_3$) δ 7.49 – 7.45 (m, 2H), 7.43 – 7.39 (m, 2H), 7.37 – 7.33 (m, 1H), 7.21 (dd, *J* = 8.3, 8.3 Hz, 1H), 6.35 (dd, *J* = 8.3, 0.8 Hz, 1H), 6.30 (d, *J* = 8.3 Hz, 1H), 5.17 (s, 2H), 4.30 (br s, 2H); ^{13}C NMR (125 MHz, $CDCl_3$) δ 161.0, 151.1, 136.1, 134.9, 128.6, 128.1, 127.0, 115.4, 107.7, 101.1, 87.2, 70.4; LCMS *m/z* 225 $[M+H]^+$; HRMS *m/z* calcd for $C_{14}H_{13}N_2O$ (M+H) 225.1022, found 225.1015. Applying general procedure G to 2-amino-6-(benzyloxy)benzonitrile gave crude 2-amino-6-(benzyloxy)benzoic acid as a yellow powder used directly without further purification. LCMS *m/z* 226 $[M+H]^+$. Following general procedure A gave 5-(benzyloxy)quinazolin-4(3*H*)-one as a white powder (0.14 g, 0.56 mmol, 14% over 2 steps). M.p. 246-247 °C; ^{1}H NMR (500 MHz, DMSO-d_6) δ 11.92 (s, 1H), 7.99 (d, *J* = 2.9 Hz, 1H), 7.68 (dd, *J* = 8.2, 8.2

Hz, 1H), 7.64 – 7.59 (m, 2H), 7.43 – 7.39 (m, 2H), 7.34 – 7.30 (m, 1H), 7.20 (dd, *J* = 8.1, 1.0 Hz, 1H), 7.12 – 7.09 (m, 1H), 5.26 (s, 1H); ^{13}C NMR (125 MHz, DMSO-d_6) δ 159.3, 138.9, 151.8, 146.3, 137.5, 135.0, 128.8, 127.9, 127.4, 119.8, 113.3, 110.3, 70.3; LCMS *m/z* 252 [M+H]$^+$; HRMS *m/z* calcd for $C_{15}H_{13}N_2O_2$ (M+H) 253.0972, found 253.0970. Applying general procedure **C** to 5-(benzyloxy)quinazolin-4(3*H*)-one gave 5-(benzyloxy)-4-chloroquinazoline as a yellow powder (0.12 g) used directly in the next step. LCMS *m/z* 271 [M+H]$^+$. Applying general procedure E to 5-(benzyloxy)-4-chloroquinazoline gave (**28**) (31 mg, 0.08 mmol, 39% over 2 steps) as a yellow oil. ^{1}H NMR (500 MHz, DMSO-d_6) δ 8.36 (s, 1H), 8.15 (d, *J* = 7.2 Hz, 1 H), 7.65 (dd, *J* = 8.1, 8.1 Hz, 1H), 7.59 – 7.56 (m, 2H), 7.48 – 7.44 (m, 2H), 7.43 – 7.39 (m, 1H), 7.25 (dd, *J* = 8.4, 1.0 Hz, 1H), 7.13 (dd, *J* = 8.1, 1.0 Hz, 1H), 5.37 (d, *J* = 2.5 Hz, 2H), 4.77 (d, *J* = 5.5 Hz, 1H), 4.63 (t, *J* = 5.2, 5.2 Hz, 1H), 4.45 (d, *J* = 4.4 Hz, 1H), 4.41 (t, *J* = 7.3 Hz, 1H), 3.63 (q, *J* = 4.8 Hz, 1H), 3.44 (q, *J* = 5.5 Hz, 1H), 3.34 – 3.25 (m, 2H), 2.29 – 2.22 (m, 1H), 1.97-1.90 (m, 1H), 0.92 – 0.85 (m, 1H); ^{13}C NMR (125 MHz, DMSO-d_6) δ 159.8, 155.9, 155.6, 151.9, 136.3, 133.1, 129.3, 129.0, 128.6, 120.4, 107.8, 106.6, 77.2, 72.7, 71.3, 63.2, 55.8, 45.5, 30.8; LCMS *m/z* 382 [M+H]$^+$; HRMS *m/z* calcd for $C_{21}H_{24}N_3O_4$ (M+H) 383.1792, found 383.1787.

4-((((1*R*,2*R*,3*S*,4*R*)-4-(5-(Benzyloxy)quinazolin-4-ylamino)-2,3-dihydroxycyclopentyl)-methoxy)methyl)benzonitrile (**29**)

(1*R*, 2*S*, 3*R*, 5*R*)-3-Amino-5-(hydroxymethyl)cyclopentane-1,2-diol hydrochloride (1.00 g, 5.45 mmol) was combined with acetone (15.0 mL) to afford a white precipitate. *p*-Toluenesulfonic acid monohydrate (1.04 g, 5.45 mmol) was added, followed by 2,2-dimethoxypropane (1.34 mL, 10.9 mmol), and the reaction mixture was stirred at room temperature for 1 h. An additional portion of *p*-toluenesulfonic acid monohydrate (1.04 g, 5.45 mmol) was added and the reaction mixture was stirred at room temperature for a further 1 h. An additional portion of 2,2-dimethoxypropane (1.34 mL, 10.9 mmol) was added and the reaction mixture was stirred at room temperature for 1 h. The homogeneous brown reaction mixture was diluted with water (3 mL), neutralised to pH 7 with the minimum saturated aqueous $NaHCO_3$ and concentrated *in vacuo* to give ((3a*R*, 4*R*, 6*R*, 6a*S*)-6-amino-2,2-dimethyltetrahydro-3*H*-cyclopenta[*d*][1,3]dioxol-4-yl)methanol[21] as a colourless oil which was used directly without further purification (1.9 g, 5.30 mmol, 97%). ^{1}H NMR (500 MHz, $CDCl_3$) δ 4.80 (d, *J* = 5.5 Hz, 1H), 4.28 (d, *J* = 5.5 Hz, 1H); 3.75 (ddd, *J* = 11.2, 3.9 Hz, 1H), 3.58 – 3.55 (m, 2H), 2.54 – 2.43 (m, 2H), 1.45 (s, 3H), 1.30 (s, 3H); LCMS *m/z* 187 [M+H]$^+$. To a solution of crude ((a3*R*, 4*R*, 6*R*, 6a*S*)-6-amino-2,2-

dimethyltetrahydro-3*H*-cyclopenta[*d*][1,3]dioxol-4-yl)methanol (2.00 g, 5.56 mmol) in methanol (20 mL) was added triethylamine (0.94 mL, 6.68 mmol), di-*tert*-butyl dicarbonate (1.36 mL, 5.84 mmol) and DMAP (7 mg, 0.06 mmol). The reaction mixture was stirred at room temperature for 16 h. Solvents were removed *in vacuo* and the residue was treated with saturated aqueous $NaHCO_3$ (20 mL) and extracted with ethyl acetate (3 x 20 mL). The combined organic layers were dried, filtered and concentrated to give *tert*-butyl (3a*S*,4*R*,6*R*,6a*R*)-6-(hydroxymethyl)-2,2-dimethyltetrahydro-3*H*-cyclopenta[*d*][1,3]dioxol-4-ylcarbamate[22] as a pale yellow oil (1.37 g, 4.77 mmol, 85%). ^1H NMR (500 MHz, $CDCl_3$) δ 5.80 (br s, 1H), 4.49 (dd, *J* = 5.9, 2.3 Hz, 1H), 4.34-4.31 (m, 1H), 3.97 – 3.90 (m, 1H), 3.71 (dd, *J* = 10.3, 4.4 Hz, 1H), 3.57 (dd, *J* = 10.3, 4.4 Hz, 1H), 2.42 – 2.34 (m, 1H), 2.24 – 2.18 (m, 1H), 1.47-1.35 (m, 13H), 1.22 (s, 3H); ^{13}C NMR (125 MHz, $CDCl_3$) δ 155.6, 110.9, 87.1, 83.3, 79.5, 63.8, 46.8, 33.4, 28.4, 27.0, 24.6; LCMS *m/z* 188 $[M+H-^tBuOCO]^+$; HRMS *m/z* calcd for $C_{14}H_{25}NNaO_5$ (M+Na) 310.1625, found 310.1623. Sodium hydride (60% wt. in oil, 0.24 g, 6.20 mmol) was added to a solution of *tert*-butyl (3a*S*,4*R*,6*R*,6a*R*)-6-(hydroxymethyl)-2,2-dimethyltetrahydro-3*H*-cyclopenta[*d*][1,3]-dioxol-4-ylcarbamate (1.37 g, 4.77 mmol) in anhydrous tetrahydrofuran (16 mL) and the mixture was stirred at room temperature for 15 min. 4-(Bromomethyl)-benzonitrile (1.03 g, 5.24 mmol) was added and the reaction mixture was stirred for 2 h at room temperature. The reaction was quenched by dropwise addition of water (5 mL). The mixture was concentrated and the residue was dissolved in ethyl acetate (20 mL) and washed with water (2 x 20 mL) and brine (20 mL). The organic layer was dried, filtered and concentrated. Flash column chromatography on silica, eluting with a gradient of 20 – 40% ethyl acetate – petroleum ether, gave *tert*-butyl (3a*S*, 4*R*, 6*R*, 6a*R*)-6-((4-cyanobenzyloxy)methyl)-2,2-dimethyltetrahydro-3*H*-cyclopenta[*d*][1,3]dioxol-4-ylcarbamate (1.32 g, 3.28 mmol, 69%) as yellow crystals. M.p. 82-83 ^oC; ^1H NMR (500 MHz, $CDCl_3$) δ 7.60 (d, *J* =8.2 Hz, 2H), 7.41 (d, *J* = 8.2 Hz, 2H), 5.28 (br s, 1H), 4.56 (s, 2H), 4.49 – 4 .46 (m, 1H), 4.36 – 4.33 (m, 1H), 4.00 – 3.94 (m, 1H), 3.58 – 3.47 (m, 2H), 2.46-2.37 (m, 1H), 2.34 – 2.28 (m, 1H), 1.48 – 1.43 (m, 1H), 1.42 (s, 3H), 1.34 (s, 9H), 1.24 (s, 3H); ^{13}C NMR (125 MHz, $CDCl_3$) δ 155.2, 143.2, 132.3, 128.0, 118.7, 111.5, 111.3, 86.8, 83.0, 79.0, 72.6, 72.5, 45.2, 33.9, 28.4, 27.2, 24.8; LRMS *m/z* 403 $[M+H]^+$; HRMS *m/z* calcd for $C_{22}H_{30}N_2NaO_5$ (M+Na) 426.2079, found 426.2066. Trifluoroacetic acid (0.023 mL) was added dropwise to a solution of (3*S*,4*R*,6*R*,6*R*)-6-((4-cyanobenzyloxy)methyl)-2,2-dimethyltetrahydro-3*H*-cyclopenta[*d*][1,3]dioxol-4-ylcarbamate (0.12 g, 0.30 mmol) in anhydrous dichloromethane (0.023 mL) and the reaction was stirred at room temperature for 2 h. The reaction mixture was azeotroped with toluene (3 x 1 mL) to afford 4-((((3a*R*, 4*R*, 6*R*, 6a*S*)-6-amino-2,2-dimethyltetrahydro-3*H*-

cyclopenta[*d*][1,3]dioxol-4-yl)methoxy)methyl)benzonitrile trifluoroacetate salt as a white powder (0.12 g, 0.29 mmol, 95%) which was used directly in the next step. ^{1}H NMR (500 MHz, DMSO) δ 8.13 (br s, 2H), 7.86 (d, *J* =8.2 Hz, 2H), 7.54 (d, *J* = 8.2 Hz, 2H), 4.62 (s, 2H), 4.47 (d, *J* = 2.9 Hz, 2H), 3.52 (s, 1H), 3.50 (s, 1H), 2.31-2.20 (m, 2H), 1.64-1.56 (m, 1H), 1.44 (s, 3H), 1.26 (s, 3H); ^{13}C NMR (126 MHz, DMSO) δ 132.8, 128.7, 128.5, 125.8, 113.1, 104.2, 83.1, 81.9, 71.7, 71.1, 43.8, 32.8, 27.7, 25.5. A reaction vial was charged with 4-((((3a*R*,4*R*,6*R*,6a*S*)-6-amino-2,2-dimethyltetrahydro-3*H*-cyclopenta[*d*][1,3]dioxol-4-yl)methoxy)methyl)benzonitrile trifluoroacetate (96 mg, 0.24 mmol), 5-(benzyloxy)-4-chloroquinazoline (65 mg, 0.24 mmol), triethylamine (67 µL, 0.48 mmol) and 1-butanol (0.48 mL) and heated in a microwave reactor at 125 ^{o}C for 1 h. The reaction mixture was cooled to room temperature, solvents were removed by evaporation and the residue was purified by flash column chromatography on silica, eluting with a gradient of 0–25% ethanol–dichloromethane, to give 4-((((3a*R*, 4*R*, 6*R*, 6a*S*)-6-(5-(benzyloxy)quinazolin-4-ylamino)-2,2-dimethyltetrahydro-3*H*-cyclopenta[*d*][1,3]dioxol-4-yl)methoxy)methyl)benzonitrile (21 mg, 0.039 mmol, 16%) as a yellow oil. ^{1}H NMR (500 MHz, CD_3OD) δ 8.38 (s, 1H), 7.68 (dd, *J* = 8.3, 8.3 Hz, 1H), 7.58 – 7.54 (m, 2H), 7.52 – 7.45 (m, 3H) , 7.45 – 7.42 (m, 2 H), 7.36 – 7.31 (m, 3H), 7.02 (d, *J* = 8.2 Hz, 1H), 5.10 (d, *J* = 3.7 Hz, 2H), 4.43 (s, 2H), 4.37 – 4.34 (m, 1H), 4.24 (dd, *J* = 6.2, 2.2 Hz, 1H), 4.11 (dd, *J* = 6.2, 2.2 Hz, 1H), 3.24 – 3.16 (m, 2H), 2.47 – 2.39 (m, 1H), 2.35 – 2.28 (m, 1H), 1.52 – 1.47 (m, 1H), 1.46 (s, 3H), 1.27 (s, 3H); ^{13}C NMR (125 MHz, CD_3OD) δ 159.1, 155.9, 154.8, 150.4, 144.1, 135.6, 133.1, 131.7, 128.85, 128.82, 128.79, 128.77, 128.7, 127.4, 127.3, 118.8, 118.2, 110.9, 110.6, 107.1, 86.1, 83.1, 71.7, 71.63, 71.61, 58.1, 45.0, 32.3, 25.8, 23.4; LCMS *m/z* 537 [M+H]$^{+}$; HRMS *m/z* calcd for $C_{32}H_{33}N_4O_4$ (M+H) 537.2496, found 537.2474. Trifluoroacetic acid (0.6 mL, 7.8 mmol) was added slowly over 5 min to a suspension of 4-((((3a*R*, 4*R*, 6*R*, 6a*S*)-6-(5-(benzyloxy)quinazolin-4-ylamino)-2,2-dimethyltetrahydro-3*H*-cyclopenta[*d*][1,3]-dioxol-4-yl)methoxy)methyl)benzonitrile (20 mg, 0.037 mmol) in water (0.6 mL). The reaction mixture was stirred at room temperature for 3 h. The mixture was concentrated and then azeotroped with toluene (3 x 3 mL) to give (**29**) (19 mg, 0.038 mmol, 99%) as a pale yellow oil. ^{1}H NMR (500 MHz, CD_3OD) δ 8.66 (s, 1H), 7.98 (dd, *J* = 8.3, 8.3 Hz, 1H), 7.70 – 7.67 (m, 2H), 7.60-7.57 (m, 2H), 7.53 – 7.50 (m, 2 H), 7.48 – 7.38 (m, 4H), 7.35 (d, *J* = 8.3 Hz, 1H), 5.38 (d, *J* = 3.8 Hz, 2H), 4.82 – 4.75 (m, 1H), 4.63 – 4.56 (m, 2H) 3.77 (dd, *J* = 4.6, 4.6 Hz, 1H), 3.63 – 3.59 (m, 1H), 3.42 (d, *J* = 5.5 Hz, 1H), 2.45 – 2.37 (m, 1H), 2.29 – 2.22 (m, 1H), 1.10 – 1.03 (m, 1H); ^{13}C NMR (125 MHz, CD_3OD) δ 161.3, 157.0, 150.6, 144.4, 139.4, 136.9, 134.8, 131.9, 129.1, 128.9, 128.5, 127.7, 127.0, 118.3, 110.9, 110.8, 110.3, 76.9, 72.8, 72.5, 71.8, 71.7, 57.0, 43.3,

29.7; LCMS *m/z* 497 $[M+H]^+$; HRMS *m/z* calcd for $C_{29}H_{29}N_4O_4$ (M+H) 497.2183, found 497.2161.

(1*S*,2*R*,3*R*,5*R*)-3-(Hydroxymethyl)-5-(5-(pyridin-2-ylmethoxy)quinazolin-4-ylamino)cyclopentane-1,2-diol (**30**)

Sodium hydride (60% in oil, 0.18 g, 4.6 mmol) was added portion-wise to 2-pyridyl benzylalcohol (0.48 ml, 4.6 mmol) in anhydrous THF (15 mL) at 0 °C over a period of 10 min under a nitrogen atmosphere. The resulting solution was stirred at 0 °C for 1 h and then 2-amino-6-fluorobenzonitrile (0.56 g, 4.1 mmol) was added in one portion and the reaction mixture heated at 85 °C for 2 h. The reaction was cooled, quenched with water and solvents were evaporated. The heterogeneous aqueous mixture was extracted with ethyl acetate (3 x 15 mL). The combined organic layers were dried, filtered and concentrated. Flash column chromatography on silica, eluting with 2.5% methanol – dichloromethane, gave 2-amino-6-(pyridin-2-ylmethoxy)benzonitrile (0.62 g, 2.8 mmol, 60%) as a white powder. M.p. 145-146 °C; ^{1}H NMR (500 MHz, $CDCl_3$) δ 8.59 (ddd, *J* = 4.9, 1.8, 0.9 Hz, 1H), 7.79 (td, *J* = 7.7, 1.8 Hz, 1H), 7.68 (dp, *J* = 7.9, 0.9 Hz, 1H), 7.30 – 7.17 (m, 2H), 6.36 (dd, *J* = 8.3, 0.7 Hz, 1H), 6.31 (dd, *J* = 8.3, 0.7 Hz, 1H), 5.28 (s, 2H), 4.47 (s, 2H); ^{13}C NMR (125 MHz, $CDCl_3$) δ 160.6, 156.3, 151.1, 149.0, 137.2, 134.9, 122.8, 121.1, 115.4, 107.9, 100.9, 87.0, 70.8; LCMS *m/z* 226 $[M+H]^+$; HRMS *m/z* calcd for $C_{13}H_{12}N_3O$ (M+H) 226.0975, found 226.0973. 2-Amino-6-(pyridin-2-ylmethoxy)benzonitrile (0.17 g, 0.76 mmol) was added in portions over 1 h to a refluxing mixture of formic acid (3.0 mL, 78 mmol) and concentrated sulfuric acid (0.18 mL, 3.4 mmol). After 30 min the reaction mixture was cooled to 0 °C and poured into ice-water (50 mL). The solution was basified with saturated aqueous $NaHCO_3$ and extracted with ethyl acetate (3 x 10 mL). The combined organic layers were dried, filtered and concentrated to give 5-(pyridin-2-ylmethoxy)quinazolin-4(3*H*)-one (0.12 g, 0.47 mmol, 62%) as a pale grey powder. M.p. 209-210 °C; ^{1}H NMR (500 MHz, DMSO) δ 11.96 (s, 1H), 8.58 (ddd, *J* = 4.8, 1.8, 0.9 Hz, 1H), 8.01 (s, 1H), 8.00 (s, 1H), 7.89 (td, *J* = 7.7, 1.8 Hz, 1H), 7.70 (t, *J* = 8.2 Hz, 1H), 7.35 (ddd, *J* = 7.5, 4.8, 1.2 Hz, 1H), 7.22 (dd, *J* = 8.1, 1.0 Hz, 1H), 7.13 (dd, *J* = 8.3, 1.0 Hz, 1H), 5.30 (s, 2H); ^{13}C NMR (125 MHz, DMSO) δ 169.7, 158.5, 157.5, 151.9, 149.2, 146.3, 137.4, 135.1, 130.5, 123.1, 121.6, 120.0, 110.2, 71.1; LCMS *m/z* 254 $[M+H]^+$; HRMS *m/z* calcd for $C_{14}H_{12}N_3O_2$ (M+H) 254.0924, found 254.0927. 5-(Pyridin-2-ylmethoxy)quinazolin-4(3*H*)-one (0.05 g, 0.20 mmol) and BOP (0.11 g, 0.24 mmol) were added to a nitrogen-flushed flask, to which was added acetonitrile (13.5 mL) and DBU (0.068 mL, 0.45 mmol).

The solution was stirred at room temperature for 5 min, then (1*R*, 2*S*, 3*R*, 5*R*)-3-amino-5-(hydroxymethyl)cyclopentane-1,2-diol hydrochloride (0.036 g, 0.197 mmol) was added and the reaction mixture was stirred at room temperature for 16 h. The mixture was concentrated and the residue purified by flash column chromatography on silica gel, eluting with a gradient of 0 – 40% ethanol – dichloromethane, followed by ion exchange chromatography on SCX-II acidic resin, eluting with 0.1 M ammonia in methanol, to give (**30**) (34 mg, 0.089 mmol, 45%) as a white solid. M.p. 150 °C (sublimes); ^{1}H NMR (500 MHz, DMSO) δ 8.82 (d, *J* = 7.3 Hz, 1H), 8.70 (ddd, *J* = 4.8, 1.8, 0.8 Hz, 1H), 8.36 (s, 1H), 7.89 (td, *J* = 7.7, 1.8 Hz, 1H), 7.66 (t, *J* = 8.2 Hz, 1H), 7.54 (dt, *J* = 7.7, 1.1 Hz, 1H), 7.42 (ddd, *J* = 7.7, 4.9, 1.2 Hz, 1H), 7.26 (dd, *J* = 8.4, 0.9 Hz, 1H), 7.10 (dd, *J* = 8.0, 1.0 Hz, 1H), 5.51 – 5.41 (m, 2H), 4.77 (d, *J* = 4.7 Hz, 1H), 4.67 (t, *J* = 5.1 Hz, 1H), 4.61 – 4.51 (m, 1H), 4.49 (s, 1H), 3.77 (dt, *J* = 7.3, 4.0 Hz, 2H), 3.41 (qt, *J* = 14.8, 7.2 Hz, 2H), 2.25 (dt, *J* = 13.0, 8.4 Hz, 1H), 1.99 (tdd, *J* = 8.8, 6.5, 4.1 Hz, 1H), 1.19 (dt, *J* = 13.1, 8.7 Hz, 1H); ^{13}C NMR (125 MHz, DMSO) δ 159.8, 155.75, 155.7, 155.6, 152.0, 149.8, 137.6, 133.1, 123.8, 122.3, 120.5, 107.5, 106.7, 76.9, 72.6, 71.2, 63.1, 56.1, 45.7, 30.7; LRMS *m/z* 383 $[M+H]^+$; HRMS *m/z* calcd for $C_{20}H_{23}N_4O_4$ (M+H) 383.1714, found 383.1725.

LC-MS compound quality control methodology

LC-MS CHROMASOLV solvents, formic acid, or alternative eluent modifiers were purchased from Sigma Aldrich (Poole, UK) unless otherwise stated. Standard 5 μL sample injections (with needle wash) were made onto a Purospher STAR RP-18 endcapped column (3μm, 30 x 4 mm, encased in LiChroCART assembly, Merck KGaA, Darmstadt, Germany). Chromatographic separation was carried out at 30 °C using a 1200 Series HPLC (Agilent, Santa Clara, USA) over a 4 minute gradient elution (Fast4mins.m) from 90:10 to 10:90 water:methanol (both modified with 0.1% formic acid) at a flow rate of 1.5 mL/min. UV-Vis spectra were acquired at 254nm on a 1200 Series diode array detector (Agilent, Santa Clara, USA).

The post column eluent flow from the diode array detector was split, with 90% sent to waste. The remainder was infused into a 6520 Series Q-ToF mass spectrometer fitted with an ESI/APCI MultiMode ionisation source (Agilent, Santa Clara, USA). LC eluent and nebulising gas were introduced into the grounded nebuliser with spray direction orthogonal to the capillary axis. 2 kV was applied to the charging electrode to generate a charged aerosol. The aerosol was dried by infrared emitters (200 °C) and heated drying gas (8 L/min of nitrogen at 300 °C, 15 psi), producing ions by ESI. Aerosol and ions were

transferred by nebulising gas to the APCI zone where infrared emitters vaporized solvent and analyte. A corona discharge was produced between the corona needle and APCI counter electrode by applying a current of 4μA, ionizing the solvent to transfer charge to analyte molecules, producing ions by APCI. ESI and APCI ions simultaneously entered the transfer capillary along which a potential difference of 2.5kV was applied. The fragmentor voltage was set at 175V and skimmer at 65V.

Data was acquired in positive ionisation mode over a scan range of m/z 130-950 (scan rate 1.0) with reference mass correction at m/z 622.02896 (Hexakis (2,2-difluroethoxy)phosphazene), and 922.0098 ((1H, 1H, 3H-tetrafluoropentoxy)phosphazene). Raw data was processed using Agilent MassHunter Qualitative Analysis B.04.00.

Protein Production

Purification of full-length HSP72

Full length HSPA1A (HSP72) was amplified by PCR from the IMAGE clone 3345864 using forward primer 5'-GATCGACCATATGGCCAAAGCCGCGGCGA-3' and its reverse complement and cloned into the NdeI & EcoRI sites of a pTWOE vector, a modified version of pET-17b (Merck Chemicals Ltd, Nottingham, UK) that encodes a *N*-terminal 6x-His-tag followed by a human rhinovirus 3C protease recognition site. BL21-AI™ cells (Invitrogen, Paisley, UK) transformed with the vector containing the HSPA1A gene were grown in Luria-Bertani (LB) medium to an optical density at 600 nm (OD_{600}) of 0.6 and induced with 0.5 mM isopropyl-β-D-1-thiogalactopyranoside (IPTG) and 0.2% (*w/v*) arabinose at 20 ^{o}C for 16 hours. Cells were harvested by centrifugation for 40 minutes at 6,000 RPM and 4°C using an Avanti centrifuge J-26XP (Beckman Coulter™, High Wycombe, UK) with a JLA 8.100 rotor. Cell pellets were resuspended in 3 volumes of Lysis buffer consisting of 25 mM Tris, 50 mM NaCl, 5% (*v/v*) glycerol, 1 x cOmplete™ EDTA-free protease inhibitors (Roche, Basel, Switzerland), 25 U/mL Benzonase ® nuclease (Merck Chemicals Ltd) at pH 7.5. Cells were lysed by sonication using a Vibra-Cell™ VCX500 (Sonics & Materials Inc, Newtown, USA) with a 13 mm solid probe for 24 cycles of 5 second on, 55 second off, at 50% amplitude. Lysate was clarified by centrifugation for 30 minutes at 20,000 RPM and 4°C using an Avanti centrifuge J-26XP (Beckman Coulter™) with a JA 25.50 rotor.

The supernatant was filtered using a 1.2 μm syringe filter (Sartorius Stedim, Germany),

loaded onto a 5 mL Histrap FF column (GE Healthcare, Chalfront St. Giles, UK) equilibrated in Buffer A, comprising 25 mM Tris, 50 mM NaCl, pH 7.5, and eluted with a gradient of 0-100% Buffer B (Buffer A + 250 mM imidazole) over 10 column volumes (CV). Fractions containing full length HSP72 (FL-HSP72) were collected, concentrated, and further purified using a Superdex 200 (16/60) size exclusion column (GE Healthcare) equilibrated in 25 mM Tris, 400 mM NaCl, 2 mM EDTA 5% (*v/v*) glycerol, pH 7.5. Fractions containing FL-HSP72 were pooled and further purified to remove contaminating nucleotides using a 6 mL Resource^{TM} Q column (GE Healthcare) equilibrated in 20 mM Tris, 2 mM EDTA, 5% (*v/v*) glycerol, pH 7.5. Following a 10 CV wash with the same buffer, the protein was eluted in a gradient from 0 to 500 mM NaCl over 6 CV and subsequently loaded onto a Superdex 200 16/60 column (GE Healthcare) equilibrated in a buffer containing 25 mM Tris, 400 mM NaCl, 15 mM EDTA, 5% (*v/v*) glycerol, pH 7.5. The removal of contaminating nucleotides was monitored by measuring the ratio of absorbance at 260 nm and 280 nm (A_{260}/A_{280}) using a NanoDrop ND-1000 UV spectrophotometer (Thermofisher,Wilmington, USA). Samples with A_{260}/A_{280} below 0.6 were considered nucleotide free.

Purification of the HSP72-NBD

The coding sequence for residues 1 to 380 HSPA1A (HSP72) was amplified by PCR from the IMAGE clone 3345864 using forward primer 5'-GATCGACATATGGCCAAAGCCGCGGCGATC-3' and its reverse complement and cloned into the NdeI & BamHI sites of the pTWOE vector described above. The HSP72 NBD was expressed and the lysate was prepared as described for FL-HSP72. Lysate was loaded onto to a 5 mL Histrap FF column equilibrated in Buffer A, consisting of 25 mM Tris, 50 mM NaCl, pH 7.5, and subsequently eluted with a gradient of 0-100% Buffer B (Buffer A + 250 mM imidazole) over 10 CV. Fractions containing the HSP72 NBD were pooled, concentrated and further purified using a Superdex 200 16/60 size exclusion column equilibrated in 25 mM Tris, 400 mM NaCl, 2 mM EDTA, 5% (*v/v*) glycerol, pH 7.5. Fractions containing the HSP72 NBD were pooled and further purified by binding contaminants to a 6 mL Resource^{TM} Q column equilibrated in 20 mM Tris, 2 mM EDTA, 5% (*v/v*) glycerol, pH 7.5 and collecting the eluent containing the HSP72 NBD domain. Purified HSP72-NBD samples were checked for nucleotide contamination by measuring their A_{260}/A_{280} ratio.

Purification of the WT HSC70-NBD and S275W, S275F and S275A mutants.

The coding sequence for WT HSPA8 (HSC70) residues 1 to 381 was amplified by PCR from the IMAGE clone 2899894 using forward primer 5'-GATCGAGGATCCATGTCCAAGGGACCTGCAGTTG-3' and its reverse complement. PCR products were inserted into the BamHI & SalI sites of a pGEX-6P-1 vector (GE Healthcare), also encoding a glutathione-*S*-transferase (GST) tag cleavable with PreScission™ protease (GE Healthcare). The inactive S275W and S275F HSC70-NBD mutants were generated by site directed mutagenesis using respective forward primers 5'-CTAAGCGTACCCTCTGGTCCAGCACCCAG-3' and 5'-GCTAAGCGTACCCTCTTCTCCAGCACCCAG-3' and their reverse complements. The S275A mutant was generated by site directed mutagenesis using forward primer 5'-CTGGGTGCTGGAAGCGAGGGTACGCTTAG-3' and its reverse complement.

All HSC70-NBD variants were expressed and the cell lysates were prepared as described for FL-HSP72. The lysate was incubated with 5 mL Superglu Superflow resin (Generon, Maidenhead, UK) for 1 hour and washed sequentially with 10 or 20 CV of Binding buffer containing 25 mM Tris, 500 mM NaCl, 2 mM EDTA, 1 mM DTT, 5% (*v/v*) glycerol, pH7.5 and 10 CV of Cleavage buffer comprising 25 mM Tris, 50 mM NaCl, 5% (*v/v*) glycerol, 2 mM EDTA, 1 mM DTT, pH 7.5 in order to reduce the salt concentration. The resin was resuspended in 1 CV of cleavage buffer, followed by the addition of 50 U PreScission™ protease (GE Healthcare) per litre cell culture and left rotating at 4 ^{o}C overnight to cleave the GST-tag. Subsequently, the sample was loaded onto a filter column and the supernatant containing the HSC70-NBD was collected, concentrated, and applied to Superdex 75 16/60 column (GE Healthcare) equilibrated in 25 mM Tris, 250 mM NaCl, 2 mM EDTA, 1 mM DTT, 5% (*v/v*) glycerol, pH 7.5. Fractions containing protein were pooled and diluted with 25 mM Tris, 2 mM EDTA, 1 mM DTT, pH 7.5 and further purified by binding contaminants to a 6 mL Resource^{TM} Q column and collecting the flow through containing the respective HSC70-NBD variant. The protein sample was subsequently concentrated and dialysed into 10 mM HEPES, 150 mM NaCl, pH 7.4. Purified HSC70-NBD samples were checked for nucleotide contamination by measuring their A_{260}/A_{280} ratio. The successful introduction of the S275A, S275F and S275W mutations was confirmed at the protein level by mass spectrometry (for details of the methods see Supporting Information).

Purification of truncated BAG1 (trBAG1)

The coding sequence for a minimal version of BAG1 (residues 222 to 334) was amplified by PCR from a construct of full-length *BAG-1S*, kindly provided by Chris Prodromou (University of Sussex), using forward primers 5'-GATCGAGGATCCAACAGTCCACAGGAAGAGGTTG-3' and its reverse complement and inserted into the BamHI & SalI sites of the pGEX-6P-1, vector described above. BAG1 was expressed and lysate prepared as described for FL-HSP72. Clarified lysate was incubated with 5 mL Superglu Superflow resin for 1 hour and then washed sequentially with 40 CV of a wash buffer containing 25 mM Tris, 500 mM NaCl, 2 mM EDTA, 1 mM DTT, 5% (*v/v*) glycerol, pH7.5 and 20 CV of lysis buffer. The total sample volume was adjusted to 10 mL with lysis buffer, followed by incubation of the resin with 50 U PreScission™ protease per litre of cell culture at 4 °C overnight. Subsequently, the sample was loaded onto a filter column, the supernatant containing BAG1 was collected and the resin washed with 2 CV of lysis buffer. The eluent and wash fractions were pooled and diluted 5-fold with 25 mM Tris, 2 mM EDTA, 1 mM DTT, 5% (*v/v*) glycerol, pH 7.5 before being applied to a 6 mL Resource™ Q equilibrated in the same buffer. Following a 10 CV wash with the same buffer, BAG1 was eluted from the column in a gradient from 0 to 500 mM NaCl over 10 CV. Peak fractions were pooled and further purified over a Superdex 75 HR 16/60 column equilibrated in 25 mM HEPES, 100 mM NaCl, 1 mM DTT, 2 mM EDTA, 5% (*v/v*) glycerol, pH 7.5.

Purification of the HSC70-NBD/BAG1 complex

Purified HSC70-NBD and BAG1 were mixed in a 1:1 ratio and diluted 10 fold with Buffer A consisting of 25 mM Tris, 2 mM EDTA, 1 mM DTT, 5% glycerol (*v/v*), pH 7.5. Following incubation on ice for 20 min, the complex was applied to a 6 mL Resource™ Q equilibrated in a 99:1 (*v/v*) mixture of Buffer A:Buffer B (Buffer A + 1 M NaCl). After a wash step of 7 CV with 1% (*v/v*) Buffer B, the complex was eluted in a gradient from 1 to 50% (*v/v*) Buffer B over 30 CV. Fractions containing the HSC70-NBD/BAG1 complex were pooled and further purified using a Superdex 75 HR 16/60 column equilibrated in 25 mM HEPES, 100 mM NaCl, 1 mM DTT, 2 mM EDTA, 5% (*v/v*) glycerol, pH7.5.

Protein intact mass spectrometry

LC-MS CHROMASOLV solvents, formic acid, or alternative eluent modifiers were purchased from Sigma Aldrich (Poole, UK) unless otherwise stated. Sample injections of 0.2 µL injections (8 step custom injection program with water, methanol and acetonitrile washes) were made onto a Security Guard C8 column cartridge (4 x 3 mm, AJO-4290, Phenomenex, Torrence, USA). The sample was refrigerated at 4 ^{0}C in a G1367B auto-sampler with G1330B thermostat module prior to injection.

Chromatographic separation was carried at 60 °C out using a 1200 Series HPLC (Agilent, Santa Clara, USA) over a 1 minute gradient elution (Protein010713QuickShot.m). Sample was loaded onto the column cartridge using a G1312A binary pump dispensing a gradient from 95:5 to 10:90 water and acetonitrile (both modified with 0.1% formic acid) at a flow rate of 3 mL/min. Between 0.3 and 0.6 minutes a ten port column selection valve (G1316A column module) was used to reverse eluent flow through the column cartridge. During this stage, a second binary pump (G1312B SL) was used to elute protein off the cartridge using a gradient from 60:40 to 10:90 water and acetonitrile (both modified with 0.1% formic acid) at a flow rate of 0.5 mL/min.

The post column eluent flow was infused into a 6520 Series qToF mass spectrometer (G6520A) fitted with a dual ESI ionisation source (Agilent, Santa Clara, USA). LC eluent and nebulising gas was introduced into the grounded nebuliser with spray direction orthogonal to the capillary axis. The aerosol was dried by heated gas (10 L/min of nitrogen at 350°C, 50 psi), producing ions by ESI. Ions entered the transfer capillary along which a potential difference of 4kV was applied. The fragmentor voltage was set at 190V and skimmer at 65V.

Data was acquired in positive ionisation mode over a scan range of m/z 650-2000 (scan rate 1.0) with reference mass correction at m/z 922.009798 hexakis(1H,1H,3H-perfluoropropoxy)phosphazene. Raw data was processed using Agilent MassHunter Qualitative Analysis B.06.00.

Ligand observed NMR experiments

NMR experiments were conducted at a ^{1}H frequency of 500 MHz using a Bruker Avance 500 spectrometer (Bruker, city, country) equipped with a 1.7 mm TXI probe. All data were acquired and processed using Bruker Topspin 3.2. The Relaxation edited ^{1}H-NMR spectrum was acquired at 295K using the CPMG sequence with a spin-lock time of 600 ms. The water signal was suppressed using presaturation during relaxation delay (2 s) and by using the Watergate sequence subsequent to the CPMG sequence. For each spectrum 64 transients were acquired. The three samples for which spectra were collected consisted of 50 mM phosphate buffer at pH 7.5, 10% (v/v) D_2O and 0.5 mM compound **30**, 0.5 mM compound **30** in the presence of 20 µM HSP72, and 0.5 mM compound **30** in the presence of 20 µM HSP72 and 100 µM ATP respectively (see Figure S8).

Table S2: Data collection and refinement statistics for crystal structures of trHSC70/BAG1 complex or trHSP72 ATPases with ligands.

Protein	**HSC70/BAG1**	**HSC70/BAG1**	**HSC70/BAG1**
Ligand	**Adenosine**	**Compound 2**	**Compound 3**
PDB ID	**5AQF**	**5AQG**	**5AQH**
Method	5 mM co-crystal, 0% DMSO	50 mM soak 16h, 10% DMSO	20 mM co-crystal
Crystals			
Space group	C2	C2	C2
Lattice constants			
a (Å)	230.73	232.86	115.31
b (Å)	40.74	40.77	40.84
c (Å)	116.69	205.79	129.60
α (°)	90	90	90
β (°)	91.16	122.48	113.72
γ (°)	90	90	90
Data collection			
Beamline	Diamond I02	Diamond I24	Rigaku FRX/Pilatus300K
Date of data collection	21-05-2009	29-07-2012	13-03-2012
Wavelength (Å)	0.9796	0.9686	1.5418
Resolution range (Å)	82.87-1.88	47.59-2.24	42.32-2.00
(highest-resolution shell values)	(1.92-1.88)	(2.28-2.24)	(2.05-2.00)
Observations	260929 (5503)	256308 (7840)	368542 (13368)
Unique reflections	76667 (2352)	78546 (3770)	37928 (2812)
Completeness (%) - Inner shell	98.6	98.7	99.3
Average	86.2	98.4	100.0
Outer shell	45.8	84.3	100.0
Multiplicity	3.4 (2.3)	3.3 (2.1)	9.7 (4.8)
R_{merge} (%)	9.8 (71.7)	10.9 (95.8)	15.9 (120)
I/σ(I)	5.6 (0.7)	4.3 (0.4)	3.9 (0.6)
Mean I/σ(I)	6.2 (0.8)	5.1 (1.0)	9.7 (1.2)
$CC_{1/2}$[a]	0.994 (0.376)	0.990 (0.265)	0.995 (0.384)
Average Mosaicity (°)	0.56	0.26	0.41
Structure Solution and Refinement			
No. of copies in ASU	2	3	1
No. of amino acids	989	1481	492
No. of water molecules	930	465	216
No. of chloride ions	0	2	0
No. of magnesium ions	0	0	0
No. of ethylene glycol molecules	0	0	0
No. of glycerol molecules	12	12	7
No. of DMSO molecules	0	0	1
No. of phosphate ions	0	0	0
No. of tris molecules	2	2	0
R-factor (%)	17.0	19.2	18.4
R_{free} (%)	20.8	22.2	22.8
Ramachandran plot			
Favored (%)	99.2	99.3	99.4
Outliers (%)	0.0	0.0	0.0
RMSD bonds (Å)	0.010	0.010	0.010
RMSD angles (°)	1.01	1.06	1.07

[a] Half-dataset correlation coefficient, see: Karplus, P. A.; Diederichs, K. Linking Crystallographic Model and Data Quality. *Science* **2012,** *336,* 1030-1033.

Table S2 continued

Protein	**HSC70/BAG1**	**HSC70/BAG1**	**HSC70/BAG1**
Ligand	**Compound 5**	**Compound 6**	**GDP**
PDB ID	**5AQI**	**5AQJ**	**5AQK**
Method	100 mM soak 16h, 20% DMSO	50 mM soak 16h, 20% DMSO	100 mM co-crystal, 0% DMSO
Crystals			
Space group	C2	C2	C2
Lattice constants			
a (Å)	229.20	233.98	115.00
b (Å)	41.12	40.94	39.89
c (Å)	115.97	206.08	127.51
α (°)	90	90	90
β (°)	90.58	123.32	115.05
γ (°)	90	90	90
Data collection			
Beamline	ESRF BM16	Diamond I24	Rigaku FRX/Pilatus 300K
Date of data collection	19-02-2011	07-05-2012	24-04-2015
Wavelength (Å)	0.9775	0.9686	1.5418
Resolution range (Å)	40.96-1.98	38.99-1.96	41.38-2.09
(highest-resolution shell values)	(2.03-1.98)	(1.99-1.96)	(2.15-2.09)
Observations	78942 (859)	443465 (19662)	177055 (7278)
Unique reflections	34725 (676)	118741 (5770)	31046 (2236)
Completeness (%) - Inner shell	97.8	98.1	99.2
Average	45.7	100.0	98.3
Outer shell	13.0	99.9	93.4
Multiplicity	2.3 (1.3)	3.7 (3.4)	5.7 (3.3)
R_{merge} (%)	7.4 (66.7)	6.3 (112)	14.7 (118)
I/σ(I)	7.2 (0.8)	8.8 (0.6)	4.7 (0.6)
Mean I/σ(I)	6.4 (0.5)	10.0 (1.0)	9.5 (1.0)
$CC_{1/2}$	0.990 (0.332)	0.999 (0.333)	0.993 (0.354)
Average Mosaicity (º)	1.36	0.11	0.41
Structure Solution and Refinement			
No. of copies in ASU	2	3	1
No. of amino acids	985	1481	488
No. of water molecules	255	876	124
No. of chloride ions	0	3	0
No. of magnesium ions	0	0	0
No. of ethylene glycol molecules	0	0	0
No. of glycerol molecules	11	16	3
No. of DMSO molecules	3	3	1
No. of phosphate ions	0	0	0
No. of tris molecules	0	2	1
R-factor (%)	17.2	19.0	18.6
R_{free} (%)	24.2	22.1	23.2
Ramachandran plot			
Favored (%)	98.7	99.4	97.9
Outliers (%)	0.0	0.0	0.0
RMSD bonds (Å)	0.010	0.010	0.010
RMSD angles (°)	1.15	1.04	1.12

Table S2 continued

Protein	**HSC70/BAG1 S275W**	**HSC70/BAG1 S275F**	**HSC70/BAG1**
Ligand			**Compound 7**
PDB ID	**5AQL**	**5AQM**	**5AQN**
Method			50 mM soak 4h, 5% DMSO
Crystals			
Space group	C2	C2	C2
Lattice constants			
a (Å)	232.03	230.83	233.29
b (Å)	40.93	40.85	41.03
c (Å)	116.55	115.78	205.25
α (°)	90	90	90
β (°)	90.58	90.52	123.13
γ (°)	90	90	90
Data collection			
Beamline	ESRF ID14-4	ESRF ID14-4	Diamond I24
Date of data collection	23-09-2011	23-09-2011	07-05-2012
Wavelength (Å)	0.9393	0.9393	0.9686
Resolution range (Å)	58.27-1.69	57.89-1.63	48.83-2.45
(highest-resolution shell values)	(1.72-1.69)	(1.66-1.63)	(2.51-2.45)
Observations	461438 (11971)	682639 (14603)	203823 (15137)
Unique reflections	120610 (4784)	124835 (3783)	61173 (4478)
Completeness (%) - Inner shell	99.7	99.7	98.9
Average	97.6	92.2	99.9
Outer shell	72.9	58.1	100.0
Multiplicity	3.8 (2.5)	5.5 (3.9)	3.3 (3.4)
R_{merge} (%)	9.1 (131)	8.7 (139)	11.7 (116)
I/σ(I)	4.9 (0.5)	6.1 (0.5)	5.2 (0.6)
Mean I/σ(I)	6.3 (0.8)	10.0 (0.9)	6.1 (0.9)
$CC_{1/2}$	0.995 (0.349)	0.998 (0.319)	0.993 (0.358)
Average Mosaicity (°)	0.24	0.24	0.31
Structure Solution and Refinement			
No. of copies in ASU	2	2	3
No. of amino acids	988	986	1480
No. of water molecules	908	1016	235
No. of chloride ions	0	0	1
No. of magnesium ions	0	0	0
No. of ethylene glycol molecules	0	0	0
No. of glycerol molecules	5	6	9
No. of DMSO molecules	0	0	3
No. of phosphate ions	0	0	0
No. of tris molecules	4	4	3
R-factor (%)	16.9	16.8	19.3
R_{free} (%)	19.7	19.5	22.8
Ramachandran plot			
Favored (%)	99.1	99.2	99.1
Outliers (%)	0.0	0.0	0.0
RMSD bonds (Å)	0.010	0.010	0.010
RMSD angles (°)	0.95	0.97	1.10

Table S2 continued

Protein	**HSC70/BAG1**	**HSC70/BAG1**	**HSC70/BAG1**
Ligand	**Compound 8**	**Compound 10**	**Compound 13**
PDB ID	**5AQO**	**5AQP**	**5AQQ**
Method	50 mM soak 4h, 5% DMSO	50 mM soak 16h, 10% DMSO	50 mM soak 16h, 10% DMSO
Crystals			
Space group	C2	C2	C2
Lattice constants			
a (Å)	233.61	232.95	233.43
b (Å)	40.68	40.98	41.33
c (Å)	205.58	206.42	205.51
α (°)	90	90	90
β (°)	122.72	123.19	123.75
γ (°)	90	90	90
Data collection			
Beamline	Diamond I24	Diamond I24	Diamond I24
Date of data collection	07-05-2012	29-07-2012	29-07-2012
Wavelength (Å)	0.9686	0.9686	0.9686
Resolution range (Å)	98.27-2.12	48.74-2.08	47.86-2.72
(highest-resolution shell values)	(2.16-2.12)	(2.12-2.08)	(2.82-2.72)
Observations	313158 (15219)	296599 (5045)	155544 (14330)
Unique reflections	93349 (4495)	90334 (2594)	44876 (4612)
Completeness (%) - Inner shell	99.7	98.8	99.1
Average	99.5	90.7	99.8
Outer shell	97.5	53.9	99.0
Multiplicity	3.4 (3.4)	3.3 (1.9)	3.5 (3.1)
R_{merge} (%)	8.6 (96.5)	9.1 (128)	25.4 (134)
I/σ(I)	3.5 (0.7)	5.4 (0.5)	1.8 (0.4)
Mean I/σ(I)	8.0 (1.2)	6.2 (0.8)	2.8 (1.0)
$CC_{1/2}$	0.977 (0.400)	0.994 0.295)	0.945 (0.344)
Average Mosaicity (º)	0.20	0.22	0.45
Structure Solution and Refinement			
No. of copies in ASU	3	3	3
No. of amino acids	1480	1478	1462
No. of water molecules	671	588	58
No. of chloride ions	3	0	0
No. of magnesium ions	0	0	0
No. of ethylene glycol molecules	0	0	0
No. of glycerol molecules	21	14	3
No. of DMSO molecules	3	5	3
No. of phosphate ions	0	0	0
No. of tris molecules	3	2	1
R-factor (%)	18.0	18.1	19.5
R_{free} (%)	21.0	20.9	24.1
Ramachandran plot			
Favored (%)	99.3	99.7	98.3
Outliers (%)	0.0	0.0	0.0
RMSD bonds (Å)	0.010	0.010	0.010
RMSD angles (°)	1/04	1.05	1.11

Table S2 continued

Protein	**HSC70/BAG1**	**HSC70/BAG1**	**HSC70/BAG1**
Ligand	**Compound 16**	**Compound 22**	**Compound 23**
PDB ID	**5AQR**	**5AQS**	**5AQT**
Method	100 mM soak 16h, 10% DMSO	400 mM soak 16h, 20% DMSO	100 mM soak 16h, 10% DMSO
Crystals			
Space group	C2	C2	C2
Lattice constants			
a (Å)	232.60	230.37	115.24
b (Å)	40.99	40.54	40.84
c (Å)	207.27	116.62	131.96
α (°)	90	90	90
β (°)	122.92	90.51	117.99
γ (°)	90	90	90
Data collection			
Beamline	Diamond I04-1	Microstar/Pt135	Diamond I04-1
Date of data collection	29-09-2012	08-02-2011	29-09-2012
Wavelength (Å)	0.9200	1.5418	0.9200
Resolution range (Å)	47.88-1.91	58.31-2.00	58.26-1.90
(highest-resolution shell values)	(1.94-1.91)	(2.05-2.00)	(1.94-1.90)
Observations	430494 (15396)	124331 (965)	109820 (6260)
Unique reflections	121547 (4977)	47003 (950)	36309 (1982)
Completeness (%) - Inner shell	99.0	99.6	99.7
Average	94.5	63.7	83.9
Outer shell	77.9	17.9	70.6
Multiplicity	3.5 (3.1)	2.6 (1.0)	3.0 (3.2)
R_{merge} (%)	7.9 (144)	18.6 (82.5)	5.8 (61.0)
I/σ(I)	5.3 (0.5)	3.0 (3.8)	4.0 (0.7)
Mean I/σ(I)	6.5 (0.8)	3.6 (0.6)	9.3 (1.7)
$CC_{1/2}$	0.996 (0.314)	0.917 (0.506)	0.968 (0.491)
Average Mosaicity (º)	0.28	0.45	1.25
Structure Solution and Refinement			
No. of copies in ASU	3	2	1
No. of amino acids	1480	983	495
No. of water molecules	650	496	210
No. of chloride ions	1	0	0
No. of magnesium ions	0	0	0
No. of ethylene glycol molecules	0	0	0
No. of glycerol molecules	18	8	6
No. of DMSO molecules	7	0	2
No. of phosphate ions	0	0	0
No. of tris molecules	3	0	1
R-factor (%)	20.8	20.8	18.0
R_{free} (%)	23.7	26.1	22.2
Ramachandran plot			
Favored (%)	99.1	98.2	99.0
Outliers (%)	0.0	0.0	0.0
RMSD bonds (Å)	0.010	0.009	0.010
RMSD angles (°)	1.02	1.08	1.04

Table S2 continued

Protein	**HSC70/BAG1**	**HSC70/BAG1**	**HSP72**
Ligand	**Compound 26**	**Compound 28**	**Compound 23**
PDB ID	**5AQU**	**5AQV**	**5AQW**
Method	40 mM soak 24h, 20% DMSO	100 mM soak 16h, 20% DMSO	100 mM backsoak for 16h from 5 mM adenosine co-crystal, 20% DMSO
Crystals			
Space group	C2	C2	$P2_12_12_1$
Lattice constants			
a (Å)	114.02	115.05	68.94
b (Å)	40.70	40.63	70.21
c (Å)	127.42	127.92	84.77
α (°)	90	90	90
β (°)	114.26	114.65	90
γ (°)	90	90	90
Data collection			
Beamline	Diamond I24	Diamond I02	Diamond I04-1
Date of data collection	08-12-2012	17-05-2013	29-09-2012
Wavelength (Å)	0.9686	0.9795	0.9200
Resolution range (Å)	41.51-1.92	50.81-1.75	21.78-1.53
(highest-resolution shell values)	(1.97-1.92)	(1.78-1.75)	(1.56-1.53)
Observations	111163 (2677)	160895 (4935)	261697 (2719)
Unique reflections	36129 (1215)	53666 (2444)	51475 (1007)
Completeness (%) - Inner shell	99.2	98.4	99.6
Average	87.8	98.1	82.3
Outer shell	45.0	82.8	33.8
Multiplicity	3.1 (2.2)	3.0 (2.0)	5.1 (2.7)
R_{merge} (%)	5.2 (104)	3.6 (91.7)	6.7 (118)
I/σ(I)	9.1 (0.7)	10.0 (0.8)	7.4 (0.6)
Mean I/σ(I)	9.0 (0.8)	12.1 (0.9)	10.7 (1.1)
$CC_{1/2}$	0.998 (0.408)	0.999 (0.327)	0.998 (0.240)
Average Mosaicity (º)	0.23	0.23	0.41
Structure Solution and Refinement			
No. of copies in ASU	1	1	1
No. of amino acids	496	493	379
No. of water molecules	217	269	423
No. of chloride ions	0	0	0
No. of magnesium ions	1	0	1
No. of ethylene glycol molecules	0	0	12
No. of glycerol molecules	9	7	0
No. of DMSO molecules	1	3	0
No. of phosphate ions	0	0	1
No. of tris molecules	1	1	0
R-factor (%)	17.8	17.9	16.1
R_{free} (%)	20.1	19.7	18.5
Ramachandran plot			
Favored (%)	99.0	99.4	99.0
Outliers (%)	0.0	0.0	0.0
RMSD bonds (Å)	0.010	0.010	0.010
RMSD angles (°)	1.04	1.00	0.92

Table S2 continued

Protein	**HSP72**	**HSP72**
Ligand	**Compound 28**	**Adenosine**
PDB ID	**5AQX**	**5AQY**
Method	100 mM backsoak for 16h from 5 mM adenosine co-crystal, 20% DMSO	5 mM co-crystal, 0% DMSO
Crystals		
Space group	$P2_12_12_1$	$P2_12_12_1$
Lattice constants		
a (Å)	51.85	47.52
b (Å)	82.02	89.05
c (Å)	94.42	95.94
α (°)	90	90
β (°)	90	90
γ (°)	90	90
Data collection		
Beamline	Diamond I02	Diamond I02
Date of data collection	17-05-2013	17-12-08
Wavelength (Å)	0.9795	0.9728
Resolution range (Å)	47.21-2.12	47.97-1.56
(highest-resolution shell values)	(2.18-2.12)	(1.59-1.56)
Observations	153812 (758)	286172 (10432)
Unique reflections	17625 (336)	58145 (2811)
Completeness (%) - Inner shell	99.7	99.7
Average	74.9	98.9
Outer shell	17.9	97.0
Multiplicity	8.7 (2.3)	4.9 (3.7)
R_{merge} (%)	7.1 (66.9)	7.9 (130)
I/σ(I)	8.8 (1.0)	5.2 (0.5)
Mean I/σ(I)	17.3 (1.2)	9.3 (1.0)
$CC_{1/2}$	0.999 (0.347)	0.997 (0.385)
Average Mosaicity (º)	0.86	0.22
Structure Solution and Refinement		
No. of copies in ASU	1	1
No. of amino acids	387	387
No. of water molecules	107	320
No. of chloride ions	1	0
No. of magnesium ions	0	1
No. of ethylene glycol molecules	0	21
No. of glycerol molecules	0	0
No. of DMSO molecules	2	1
No. of phosphate ions	0	0
No. of tris molecules	0	0
R-factor (%)	19.4	17.4
R_{free} (%)	22.7	20.4
Ramachandran plot		
Favored (%)	98.2	99.5
Outliers (%)	0.0	0.0
RMSD bonds (Å)	0.010	0.010
RMSD angles (°)	1.08	0.97

Supplementary references

1 Witty, D. R. *et al.* (2012) ASK1 inhibiting pyrrolopyrimidine derivatives. Intl. Pat. Appl. WO2012080735

2 Sleebs, B. E. *et al.* Identification of 3-aminothieno[2,3-b]pyridine-2-carboxamides and 4-aminobenzothieno[3,2-d]pyrimidines as LIMK1 inhibitors. *Med. Chem. Comm.* **2**, 977-981 (2011).

3 Showalter, H. D. *et al.* Tyrosine kinase inhibitors. 16. 6,5,6-tricyclic benzothieno[3, 2-d]pyrimidines and pyrimido[5,4-b-] and -[4,5-b]indoles as potent inhibitors of the epidermal growth factor receptor tyrosine kinase. *J. Med. Chem.* **42**, 5464-5474 (1999).

4 Hirota, T., Saskai, K., Tashmia, Y. & Nakayama, T. Polycyclic N-hetero compounds. XXXIV. Syntheses and evaluation of antidepressive activity of benzofuro-[2,3-e]imidazo[1,2-c]pyrimidines and their precursors. *J. Het. Chem.* **28**, 263-267 (1991).

5 Okuda, K., Muroyama, H. & Hirota, T. Polycyclic N-heterocyclic compounds, part 67: Reaction of 6,7-substituted N-(quinazolin-4-yl)amidine derivatives with hydroxylamine hydrochloride: Formation of in vitro inhibitors of pentosidine. *J. Het. Chem.* **48**, 1407–1413 (2011).

6 Gewald, K., Schäfer, H. & Mauersberger, K. 4-Aminochinazoline durch intramolekulare Friedel-Crafts-Reaktion. *Z. Chem.* **17**, 223–224 (1977).

7 Hennequin, L. F. *et al.* N-(5-chloro-1,3-benzodioxol-4-yl)-7-[2-(4-methylpiperazin-1-yl)ethoxy]-5- (tetrahydro-2H-pyran-4-yloxy)quinazolin-4-amine, a novel, highly selective, orally available, dual-specific c-Src/Abl kinase inhibitor. *J. Med. Chem.* **49**, 6465-6488 (2006).

8 Baker, B. R., Schaub, R. E., Joseph, J. P., McEvoy, F. J. & Williams, J. H. An antimalarial alkaloid from Hydrangea. XIV. Synthesis of 5-, 6-, 7-, and 8-monosubstituted derivatives. *J. Org. Chem.* **17**, 141-148 (1952).

9 Beckers, T. *et al.* Chimerically designed HDAC- and tyrosine kinase inhibitors. A series of erlotinib hybrids as dual-selective inhibitors of EGFR, HER2 and histone deacetylases. *Med. Chem. Comm.* **3**, 829-835 (2012).

10 LeMahieu, R. A. *et al.* (E)-3-(4-Oxo-4H-quinazolin-3-yl)-2-propenoic acids, a new series of antiallergy agents. *J. Med. Chem.* **26**, 420-425 (1983).

11 Lippa, B. *et al.* The discovery of highly selective erbB2 (Her2) inhibitors for the treatment of cancer. *Bioorg. Med. Chem. Lett.* **17**, 3081-3086 (2007).

12 Smith, M. E., Elisberg, E. & Sherrill, M. L. Quinazoline derivatives; synthesis of 4-(4'-diethylamino-1'-methylbutylamino)-6-methoxyquinazoline (SN 12,253). *J. Am. Chem. Soc.* **68**, 1301-1303 (1946).

13 Ueda, I., Kato, M. & Nagano, M. (1981) Quinazoline derivatives, processes for their preparation and pharmaceutical compositions containing them. . EU Pat. Appl. EP19800304335

14 Chapman, N. B., Gibson, G. M. & Mann, F. G. Synthetic antimalarials 4-dialkylaminoalkylaminoquinazolines; variation of substituents in the 6- and 7-positions. *J. Chem. Soc.*, 890-899 (1947).

15 Kummerle, A. E. *et al.* Design, synthesis and analgesic properties of novel conformationally-restricted N-acylhydrazones (NAH). *Bioorg. Med. Chem. Lett.* **19**, 4963-4966 (2009).

16 Mertens, M. D., Pietsch, M., Schnakenburg, G. & Gutschow, M. Regioselective sulfonylation and N- to O-sulfonyl migration of quinazolin-4(3H)-ones and analogous thienopyrimidin-4(3H)-ones. *J. Org. Chem.* **78**, 8966-8979 (2013).

17 Nathubhai, A. *et al.* N3-alkylation during formation of quinazolin-4-ones from condensation of anthranilamides and orthoamides. *Org. Biomol. Chem.* **9**, 6089-6099 (2011).

18 Kummerle, A. E. *et al.* Design, synthesis, and pharmacological evaluation of N-acylhydrazones and novel conformationally constrained compounds as selective and potent

orally active phosphodiesterase-4 inhibitors. *J. Med. Chem.* **55**, 7525-7545 (2012).
19 Endicott, M. M., Wick, E. & et al. Quinazoline derivatives; the synthesis of 4-(4'-diethylamino-1'-methylbutylamino)-quinazoline (SN 11,534) and the corresponding 2-phenylquinazoline (SN 11,535). *J. Am. Chem. Soc.* **68**, 1299-1301 (1946).
20 Harris, N. V., Smith, C. & Bowden, K. Antifolate and antibacterial activities of 5-substituted 2,4-diaminoquinazolines. *J. Med. Chem.* **33**, 434-444 (1990).
21 Tokoro, Y. & Kobayashi, Y. Realisation of highly stereoselective dihydroxylation of a cyclopentene in the synthesis of (–)-aristeromycin. *Chem. Commun.* **9**, 807-809 (1999).
22 Kudoh, T. *et al*. Synthesis of stable and cell-type selective analogues of cyclic ADP-ribose, a Ca(2+)-mobilizing second messenger. Structure-activity relationship of the N1-ribose moiety. *J. Am. Chem. Soc.* **127**, 8846-8855 (2005).
